# Experimental Isolation of Single Impact, Cumulative Energy, Aging, and Liquid‐Assisted Grinding Effects in Mechanochemical Reactions

**DOI:** 10.1002/anie.202523191

**Published:** 2026-01-15

**Authors:** Johanna Templ, Lars Borchardt

**Affiliations:** ^1^ Inorganic chemistry II Ruhr Universität Bochum Universitätsstraße 150 44801 Bochum Germany

**Keywords:** Grinding, Mechanochemistry, Milling, Solid‐state kinetics, Surface activation

## Abstract

Mechanochemical reactivity arises from a complex interplay between single impact energy, cumulative energy input, and secondary effects such as aging or liquid‐assisted grinding (LAG). Here, we experimentally isolate these factors across two fundamentally different all‐solid reactions–the Finkelstein halogen exchange and the Wittig olefination–using a standardized single‐ball mixer mill setup. Systematic variation of milling parameters revealed that both reactions are impact‐driven but differ in how mechanical energy translates into chemical conversion. The halogen exchange exhibited pronounced aging, where the transformation continues after mechanical activation, highlighting the contribution of post‐impact reactivity. In contrast, the Wittig olefination, free of such secondary effects, showed an almost ideal linear correlation between yield and cumulative energy (*E*_total_). LAG additives were found to modulate but not replace the impact‐driven regime, providing mechanistic insight into the frequently observed *sweet spot* in LAG‐assisted reactions.

## Graphical abstract

An experimental framework is introduced to isolate single‐impact and cumulative energy within a mechanochemical system. The guided, stepwise approach highlights the fundamental roles of impact events, post‐impact reactivity, and liquid‐assisted grinding additives in all‐solid mechanochemical reactions.

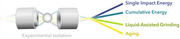

## Introduction

As a response to rising environmental concerns and the pressing need for a shift toward greener syntheses, mechanochemistry has become firmly established in modern chemistry, showing potential to redefine traditional approaches in catalytic transformations,^[^ ^1^, ^2^, ^3^, ^4^, ^5^, ^6^, ^7^, ^8^ ^]^ pharmaceutical syntheses,^[^ ^9^, ^10^, ^11^, ^12^, ^13^ ^]^ or polymer synthesis and degradation.^[^ ^14^, ^15^, ^16^, ^17^, ^18^ ^]^ Although ball milling strategies have made rapid headway in applied syntheses, a fundamental understanding of the underlying principles regarding kinetic energy provision and utilization, as well as how exactly impact forces trigger chemical transformations, is still emerging and remains a topic of ongoing research, guided by key fundamental works.^[^ ^19^, ^20^, ^21^, ^22^, ^23^, ^24^, ^25^, ^26^, ^27^, ^28^, ^29^, ^30^, ^31^ ^]^ Ideally, in the near future, we could predict the outcome of mechanochemical reactions, ensure their reproducibility and have parameters that allow for their reliable transfer between mechanochemical devices that operate on different modes of mechanical energy provision (e.g., planetary mills, mixer mills, resonant acoustic mixers, extruders).^[^ ^32^, ^33^, ^34^, ^35^ ^]^ However, this involves more than the precise quantification of the dose of kinetic energy delivered to the system.^[^ ^21^, ^27^ ^]^ We also need to gain a deeper understanding of how exactly solid reactants interact under mechanical stress, how specific ball milling parameters influence reactivity and selectivity, and how secondary effects such as aging triggered by surface activation or enhanced diffusion enabled by liquid‐assisted grinding (LAG) affect the reaction outcome.^[^ ^30^, ^36^, ^37^, ^38^, ^39^, ^40^, ^41^, ^42^ ^]^ Several groups have made considerable efforts to develop models that quantify the kinetic energy of the system or shine light on specific ball milling parameters.^[^ ^25^, ^26^, ^27^, ^28^, ^29^, ^30^, ^31^, ^32^, ^33^, ^36^, ^43^, ^44^, ^45^, ^46^, ^47^, ^48^, ^49^, ^50^, ^51^ ^]^ Typically, these studies have either applied newly developed models to a single representative reaction or examined specific milling parameters using one selected transformation. Such focused investigations are extremely valuable to gain deeper insight into the underlying principles of mechanochemical reactions. However, the narrow experimental scope of single‐reaction studies can limit the ability to identify interdependent effects or uncover unexpected correlations between parameters that influence the global reaction outcome. Furthermore, since mechanochemical transformations can vary substantially in their intrinsic kinetics, individual reactions may respond differently to specific milling parameters, thereby limiting the general validity of conclusions derived from a single‐reaction system.

With the herein presented study, we aimed to switch perspective on how kinetic energy and milling parameters influence mechanochemical reactivity by applying a systematic, top‐down experimental approach. Rather than focusing on a single model transformation, we selected fundamentally different reactions that have not previously been used for mechanochemical kinetic studies and first examined their overall transformation behavior. Building on these results, we then aimed to disentangle the individual contributions of single impact energy, cumulative energy, aging, and liquid‐assisted grinding, answering the following questions:

‐ How well does the cumulative energy predict reaction yield across different milling conditions?
‐ Is reaction progress driven primarily by impact energy?
‐ Does the addition of a liquid‐assisted grinding (LAG) agent enhance diffusion enough to outweigh the role of impact energy?

## Results and Discussion

The reactions selected for this kinetic study were chosen based on well‐defined criteria rather than preconceived expectations of specific outcomes, ensuring a representative range of mechanochemical systems. We focused on systems that meet several essential criteria for mechanochemical kinetic investigations in an all‐solid reactant protocol. First, the reaction should ideally reach full conversion without side reactions but be interruptible at defined timepoints before completion. This is crucial to observe potential effects that may enhance reaction yield; if full conversion is reached too early, further energy input will not affect the yield, possibly evoking misleading conclusions in studies based on constant cumulative energy (*E*_total_).^[^ ^32^, ^33^ ^]^ Second, the reaction must be exclusively triggered by mechanical energy input and not by solvent addition during subsequent analysis (verified through control experiments, see Supporting Information). Third, we prioritized all‐solid reactions, avoiding systems that involve liquid or low‐melting reactants, products, or by‐products. This allows the controlled and deliberate addition of liquids only when studying LAG effects and prevents the reaction from proceeding through solution‐like pathways within liquid microenvironments. Finally, reactions exhibiting auto‐catalysis, self‐acceleration, or self‐limitation were avoided to ensure clean kinetic evaluation. After screening various candidates, we identified two fundamentally different transformations that fulfill these criteria:

The **Finkelstein halogen‐exchange reaction** between 4‐(bromomethyl)‐1,1′‐biphenyl **(1)** and sodium iodide (NaI), and the **Wittig olefination** of biphenyl‐4‐carboxaldehyde **(3)** using methyltriphenylphosphonium bromide (PPh_3_MeBr) and cesium carbonate (Cs_2_CO_3_) as base. In addition, we briefly examined the **KMnO_4_‐mediated oxidation** of 1‐(4‐biphenyl)ethanol. Although this reaction was gradually inhibited by the accumulation of MnO_2_, it provided valuable insight into all‐solid transformations influenced by mechanically induced energy. For conciseness, the detailed results of this oxidation study are given exclusively in the Supporting Information.

In the following, we present our kinetic findings for each reaction separately, beginning with the halogen‐exchange reaction, before comparing the observed mechanistic trends to those of the Wittig olefination. All experiments were performed in a horizontal mixer mill using a simple, well‐defined milling setup with a perfluoroalkoxy (PFA) milling jar of fixed dimensions (14 mL volume, 20 mm inner diameter). The jar material was kept constant to ensure that non‐linear effects arising from powder compaction, elastic deformation of the jar, and energy dissipation during collisions remain as consistent as possible across experiments.^[^ ^26^, ^27^, ^31^, ^46^ ^]^ A single milling ball of 15 mm diameter was employed in each reaction, with the ball material varied between experiments. This near‐fit between ball and inner jar diameter minimizes complex trajectories and ensures primarily linear back‐and‐forth movement, simplifying energy considerations.^[^ ^52^ ^]^ The single impact energy (*E*_impact_) and cumulative energy (*E*_total_) were calculated according to Lungerich.^[^ ^32^ ^]^ For the presented single‐ball system, *E*_impact_ scales proportionally with the ball mass, and hence directly with the ball material density. Although absolute energy values are debated in the literature,^[^ ^32^, ^33^ ^]^ this proportionality allows valid *relative* energy comparisons within the same system. Thus, even if calculated values deviate slightly from absolute experimental energy measurements, the *relative trends* between conditions remain robust. All data points represent triplicate reactions to ensure reproducibility and precision; observed deviations fall within typical human and instrumental error margins (for detailed results including standard deviations see Supporting Information).

### The Finkelstein Halogen‐Exchange Reaction

**Figure 1:**
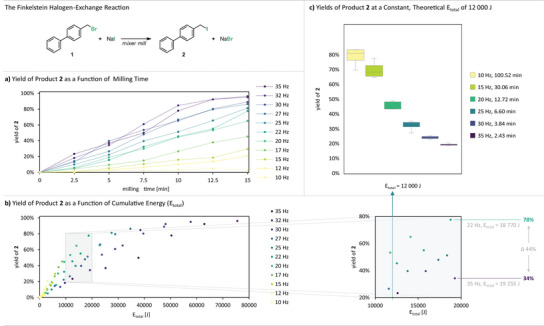
Results from the halogen‐exchange reaction at different frequencies and milling times. Yields of **2** were determined via quant. ^1^H‐NMR using benzyl benzoate as an internal standard; datapoints represent triplicate reactions. a) Yields plotted against the milling time. b) Yields plotted against the cumulative energy (*E*_total_) (left) and section of the plot showing significant yield differences at comparable *E*_total_ values (right). c) Yields of **2** for reactions performed at different milling frequencies with a constant, calculated *E*_total_ of 12 000 J.

We commenced our kinetic study with the halogen‐exchange reaction between 4‐(bromomethyl)‐1,1′‐biphenyl **(1)** and NaI, performed under solvent‐free conditions using a single 15 mm ZrO_2_ ball in a 14 mL PFA jar (for experimental details see Supporting Information). Milling frequencies were systematically varied from 10 to 35 Hz in increments of 2‐3 Hz, and reaction times were gradually increased in 2.5‐min steps (Figure 1a). As expected, longer milling times and higher frequencies consistently resulted in higher product yields of **2**. This trend reflects the increase in total mechanical energy input both through higher *E*_impact_ and longer energy accumulation times. In a simplified theoretical picture, one would expect the reaction yield to linearly correlate with *E*_total_, such that a plot of the reaction yield versus the cumulative energy would collapse into a single line.^[^ ^32^ ^]^ However, our experiments revealed significant deviations from this ideal behavior (Figure 1b). At comparable *E*_total_ values, reactions performed at lower frequencies but longer times often gave higher yields–in some cases up to 40% greater–than high‐frequency reactions of shorter duration (see Figure 1b, **right** and Supporting Information for full datasets incl. temperature measurements).

To probe this phenomenon further, reactions were performed at a constant cumulative energy (*E*_total_ = 12 000 J) by varying reaction time for each frequency to reach the target *E*_total_ (Figure 1c). If yield scaled solely with total energy input, these reactions should give comparable results. Instead, large yield differences for product **2** (up to 60%) were observed. Therefore, it becomes evident that, for this specific reaction, secondary effects indirectly related to the mechanical energy input exert a pronounced influence on the reaction yield. To disentangle and isolate these contributing factors step‐by‐step, we designed experimental series in which, for each set, one parameter was held constant or systematically controlled while the others were varied.

At constant *E*_total_, longer reactions at lower frequencies consistently gave higher yields than short high‐frequency reactions (e.g., 100.52 min at 10 Hz versus 2.43 min at 35 Hz; see Figure 1c) even though the corresponding macroscopic reaction temperatures differed by less than 7 °C. This observation prompted us to consider whether the kinetics might be dominated solely by the total number of impacts (N_i_), corresponding to how many times the ball travels back‐and‐forth during milling and triggers particle breakage. For example, a 10 Hz reaction run for 100 min involves vastly more impact events than a 35 Hz reaction run for only a few minutes, even if both deliver the same *E*_total_. Thus, if the transformation is governed primarily by the total number of impacts, which determine the maximum local degree of mixing achievable, the yield should remain independent of the milling ball weight, provided that the frequency, milling time, and ball diameter–and hence the total number of impacts (N_i_)–are kept constant.^[^ ^22^, ^23^, ^26^, ^53^ ^]^

**Figure 2:**
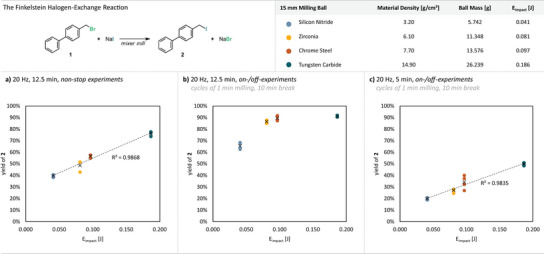
Results from the halogen‐exchange reaction performed at a constant milling frequency (20 Hz) and a constant active milling time (12.5 min) using a single 15 mm milling ball with varying material densities (top, right). The yield of product **2** was determined via quant. ^1^H‐NMR using benzyl benzoate as an internal standard and is given as a function of the single impact energy (*E*_impact_). Trendlines correlate to the average yield datapoints (black crosses) for each set of experiments. a) Reactions were performed *non‐stop* without intermittent “cooling” breaks. b) and c) Reactions were performed in repeating cycles of 1 min milling and 10 min static break to keep the reaction temperature low. The time given refers to the active milling time (12.5 min for b) and 5 min for c)).

To test this hypothesis, we conducted a set of experiments using single 15 mm milling balls of varying densities under otherwise identical conditions (20 Hz, 12.5 min; Figure 2). The balls were made of tungsten carbide (WC, 14.9 g cm^−3^), chrome steel (Fe‐Cr, 7.7 g cm^−3^), zirconia (ZrO_2_, 6.1 g cm^−3^), or silicon nitride (Si_3_N_4_, 3.2 g cm^−3^). When yield was plotted against *E*_impact_ (proportional to density), an almost perfectly linear correlation was obtained, clearly showing that the reaction is impact‐driven rather than dominated by the effective number of impacts alone (Figure 2a). Whether the reaction barrier is overcome directly by mechanical forces or indirectly via heat dissipation, resulting in transient microscopic temperature spikes at the point of impact, is extremely difficult to determine. From an energy‐conservation perspective, however, the magnitude of any such localized temperature increase would scale directly with the impact energy. Accordingly, in this study, we deliberately focused on the single impact energy as the primary driver of the reaction, without subdividing the contribution into direct mechanical versus indirect thermal effects. Macroscopic temperature effects were also considered, as heavier balls generate more overall heat during milling (see Supporting Information for detailed thermal data). Although the observed linearity suggested otherwise, we repeated the experiments in an *on/off*‐mode consisting of 1‐min milling intervals followed by 10‐min rest periods, allowing the reaction vessel to cool between impacts.

The total active milling time was maintained at 12.5 min, equivalent to the previous continuous milling experiments (Figure 2b). Interestingly, yields in the *on/off*‐experiments were generally higher, plateauing around 90% for WC, Fe–Cr, and ZrO_2_ balls, while the lighter Si_3_N_4_ ball gave slightly lower yields (around 65%) within this set of experiments. The enhanced yields despite reduced overall heating suggest that the macroscopic temperature does not fundamentally drive the reaction. However, since most reactions reached completion under these conditions, kinetic discrimination between different ball materials became difficult. When the active milling time was reduced to 5 min (with identical cooling pauses), temperature differences between light and heavy balls dropped below 5 °C (Figure 2c). Yet, the dependence of yield on *E*_impact_ persisted, reinforcing that the reaction is impact‐driven rather than governed by macroscopic temperature increases.

From these results, we conclude:

‐ The halogen‐exchange reaction is indeed impact‐driven, showing a linear dependence of yield on *E*_impact_.
‐ Neither the macroscopic temperature nor the number of net impacts alone influence the overall kinetics.
‐ The conversion of the all‐solid mixture continues after the mechanically induced activation has ended (aging). This explains the higher yields observed in prolonged, low‐frequency experiments at constant *E*_total_ and *on/off‐experiments*.

**Figure 3:**
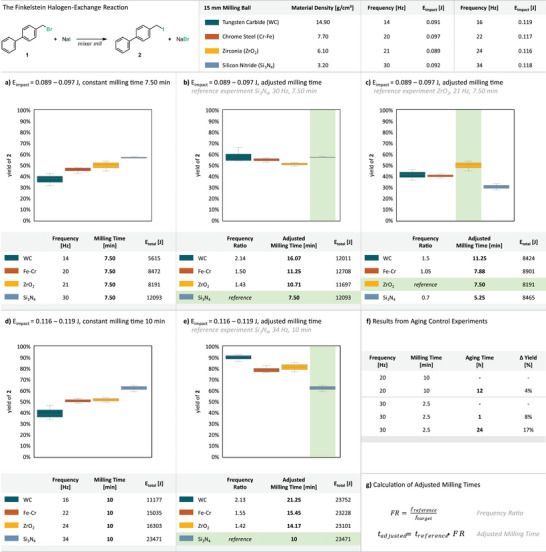
Results from the halogen‐exchange reaction performed within narrow *E*_impact_ ranges of 0.089–0.097 J a)–c) and 0.116–0.119 J d) and e) using a single 15 mm milling ball with varying densities at constant milling times a) and d) and adjusted milling times b), c), and e). Adjusted milling times were calculated to equalize the number of impacts (N_i_) and the cumulative energy (*E*_total_) with respect to a reference experiment (g). Results from aging control experiments are shown (f). Yields of **2** were determined via quant. ^1^H‐NMR using benzyl benzoate as an internal standard; Data obtained from triplicate reactions.

Having established that *E*_impact_ is a key determinant of yield, we next examined the inverse scenario: what happens when *E*_impact_ is held constant across systems with different ball materials? Since *E*_impact_ depends on both ball mass and velocity (frequency), we compensated changes in ball density by adjusting milling frequency, keeping *E*_impact_ within narrow ranges (0.089–0.097 J and 0.116–0.119 J, Figure 3; for details and additional experiments see Supporting Information). At fixed milling times, heavier balls (higher density) required lower frequencies to maintain a target *E*_impact_, whereas lighter balls necessitated higher frequencies. Consequently, low‐frequency experiments involved fewer net impacts (Nᵢ), explaining lower yields under otherwise comparable conditions (Figure 3a,d). Subsequently, the number of impacts and thus *E*_total_ were equalized across all experiments by proportionally adjusting the milling times relative to a defined reference experiment (Figure 3g; for details see Supporting Information). This strategy allowed us to isolate the effects of single impact energy from those of the cumulative number of impacts.

When comparing reactions equalized for both N_i_ and *E*_total_ within the 0.089–0.097 J range, the results were strongly dependent on the chosen reference. Using a high‐frequency reference (e.g., Si_3_N_4_ at 30 Hz), prolonged milling times for lower‐frequency reactions gave yields comparable to the reference (Figure 3b). In contrast, using a mid‐frequency reference (ZrO_2_, 21 Hz), adjusted reactions gave significantly lower yields (Figure 3c). These observations suggest that the duration of post‐impact processes (e.g., aging) contributes to conversion, with longer milling times allowing more aging to occur. This trend becomes even more pronounced at higher *E*_impact_ (0.116–0.119 J). When Si_3_N_4_ at 34 Hz was used as a reference, equalizing Nᵢ and *E*_total_ required significantly longer reaction times for heavier balls (Figure 3e). Rather than matching the reference yield, these prolonged reactions gave significantly higher yields of product **2**. We propose that the combination of extended activation times and higher individual impact energies accelerates post‐impact processes such as aging. Control experiments support this interpretation: after milling at 20 Hz for 10 min, the yield increased by 4% after 12 h of static aging (Figure 3f). Although seemingly minor, this demonstrates that small, but measurable conversion continues after milling stops. At 30 Hz, aging effects were more pronounced, with yields increasing by 8% after 1 h and 17% after 24 h of static aging.

**Figure 4:**
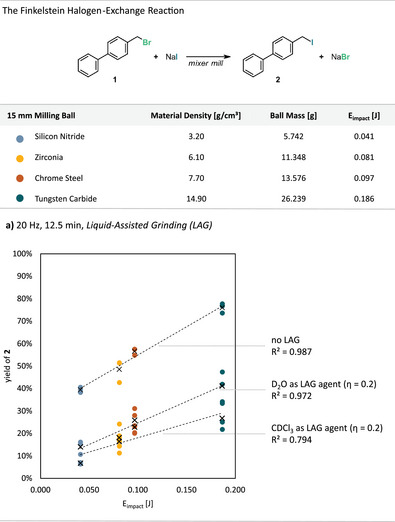
Results from the halogen‐exchange reaction performed at a constant milling frequency (20 Hz) and a constant milling time (12.5 min) using a single 15 mm milling ball with varying material densities. The yield of product **2** was determined via quant. ^1^H‐NMR using benzyl benzoate as an internal standard and is given as a function of the single impact energy (*E*_impact_). Trendlines correlate to the average yield datapoints (black crosses) for each set of experiments. a) Each set of reactions was performed either without LAG additives or adding D_2_O and CDCl_3_ (*η* = 0.2 µL·mg^−1^).

These observations suggest that both the extent and rate of aging likely correlate with *E*_impact_, explaining why equalizing *E*_total_ alone does not ensure equal yields in the halogen‐exchange reaction. To test whether surface‐modifying additives could suppress such effects, the reaction was performed under LAG conditions that potentially weaken surface interactions or disturb the all‐solid regime (Figure 4). Water or chloroform (*η* = 0.2 µL·mg^−1^) was chosen to selectively influence the solubility and surface characteristics of one reaction component, NaI or the organic substrate **(1)**, respectively. We hypothesized that introducing a small amount of solvent could diminish *E*_impact_ effects and promote diffusion. Using deuterated analogs avoided interference in NMR quantification. Each set of LAG reactions was conducted using a single 15 mm milling ball of varying densities at 20 Hz for 12.5 min. Under these conditions, overall yields were generally lower, and the regression slopes of yield versus *E*_impact_ were slightly flattened compared to neat all‐solid reactions (Figure 4a). This suggests that while the reaction remains primarily impact‐driven, the presence of a liquid phase likely perturbs surface activation and diminishes the effective energy transfer during collisions. However, since both water and chloroform are intrinsically poor solvents for this transformation, the reduced yields may also reflect slower intrinsic reaction kinetics rather than decreased impact efficiency.

Although the mechanistic interpretation should therefore be made with caution, these results underscore the high sensitivity of solid‐state reactivity to surface‐modifying additives and provide a valuable basis for future investigations into impact energy dissipation and surface deactivation under mechanochemical LAG conditions.

### The Wittig Olefination

**Figure 5:**
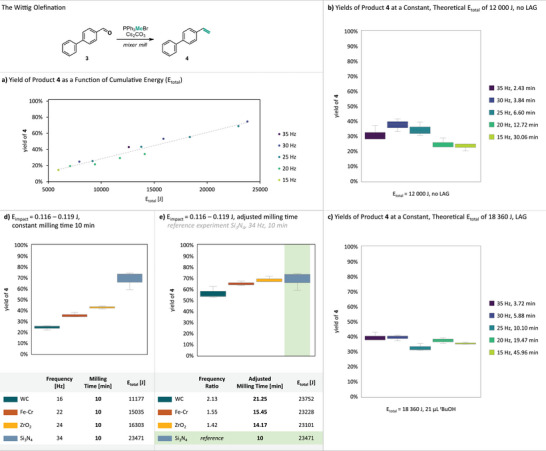
Results from the Wittig olefination at different frequencies and milling times. Yields of **4** were determined via quant. ^1^H‐NMR using dibromomethane as an internal standard; datapoints represent triplicate reactions. a) Yields plotted against the cumulative energy (*E*_total_) within a range of 5 000–25 000 J. b) Yields of reactions performed at different milling frequencies with a constant, calculated *E*_total_ of 12 000 J (b) and 18 360 J (c). And results from the Wittig olefination performed within narrow *E*_impact_ ranges of 0.116–0.119 J d) and e) using a single 15 mm milling ball with varying densities at constant milling times (d) and adjusted milling times (e).

With the all‐solid Wittig olefination of biphenyl‐4‐carboxaldehyde **(3)** to the vinylated product **4**, we identified an ideal complementary reaction to the previously studied Finkelstein halogen‐exchange.^[^ ^54^, ^55^ ^]^ All reactants and (by‐)products remain solid, and the reaction proceeds exclusively under mechanochemical conditions, allowing full conversion but also enabling sampling at intermediate timepoints to study sub‐completion kinetics. The reaction proceeds without LAG addition, although LAG agents can be purposefully introduced for mechanistic investigations. Unlike the halogen‐exchange reaction, no macroscopic aging is observed in the Wittig reaction, and conversion ceases entirely in the absence of mechanical impact. Therefore, yield variations attributed to aging or gradual self‐inhibition, as seen in the halogen‐exchange and KMnO_4_ oxidation reactions, are expected to be absent (details on the oxidation reaction and control experiments see Supporting Information). Following the approach used for the halogen‐exchange reaction (Figure 1b), Wittig olefination reactions were conducted at various frequencies and reaction times having cumulative energies (*E*_total_) between 5 000 and 25 000 J. In the halogen‐exchange reaction, significant deviations from a linear correlation between yield and *E*_total_ were observed, which we attributed to aging. In contrast, for the Wittig olefination, plotting the yield of **4** versus *E*_total_ revealed a clear linear trend. While halogen exchange yields varied by up to 40% for comparable *E*_total_ values, deviations for the Wittig reaction were below 10%, approaching the range of experimental error (Figure 5a). When *E*_total_ was kept constant at 12 000 J under neat conditions, yield differences remained below 14% (Figure 5b), compared to up to 60% for the halogen‐exchange reaction (cf. Figure 1c).

This yield‐equalization persisted under LAG conditions using *^t^* BuOH at *η* = 0.1 µL·mg^−1^ (Figure 5c). These results indicate that, in the absence of secondary effects such as aging or gradual self‐inhibition, the cumulative energy–as proposed by the Lungerich group–could serve as a valid parameter for adjusting milling conditions in simple single‐ball systems.^[^ ^32^ ^]^ Analogous to previous experiments, keeping *E*_impact_ in a narrow range of 0.116–0.119 J through variation of milling ball density and frequency while holding milling time constant again resulted in yield differences caused by unequal numbers of impacts (Figure 5d). Adjusting N_i_ to equalize *E*_total_ across experiments successfully levelled yields (Figure 5e), with slightly lower yields for the tungsten carbide system at 16 Hz, likely due to the heavy ball's sluggish movement reducing the number of effective impacts at low frequency.

**Figure 6:**
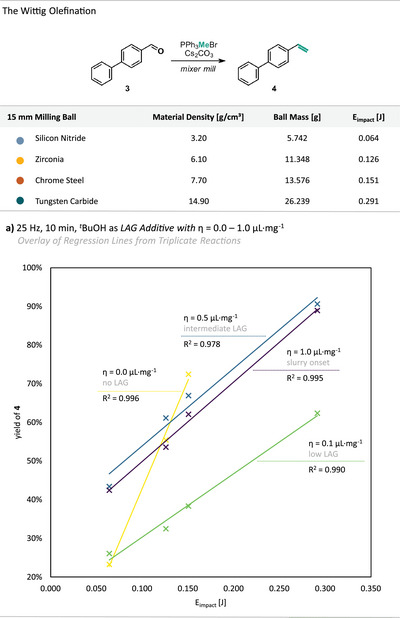
Results from the Wittig Olefination performed under different amounts of LAG additives (*^t^* BuOH) at a constant milling frequency (25 Hz) and a constant milling time (10 min) using a single 15 mm milling ball with varying material densities. The yield of product **4** was determined via quant. ^1^H‐NMR using dibromomethane as an internal standard and is given as a function of the single impact energy (*E*_impact_). a) Overlay of Regression lines with each line correlating to the average yield of triplicate reactions (crosses) for each set of experiments.

For the halogen‐exchange reaction, we observed that the addition of LAG agents, which can modify the solid‐state surface of reactants, did not change the impact‐driven nature of the transformation (Figure 4). To extend this understanding and probe the influence of LAG on energy dependence, we performed analogous experiments for the Wittig olefination, varying the amount of *^t^* BuOH from dry conditions up to the onset of the slurry regime (*η* = 0.0–1.0 µL mg^−1^, Figure 6). *^t^* BuOH was chosen as a suitable LAG agent because it can partially solubilize the highly polar reactants (Cs_2_CO_3_ and PPh_3_MeBr) without significantly accelerating the reaction, thereby allowing clear kinetic discrimination. Under neat conditions, the Wittig reaction proceeded rapidly and displayed a steep linear correlation between yield and *E*_impact_, confirming a purely impact‐driven regime. Upon addition of small LAG amounts (*η* = 0.1 µL mg^−1^), overall yields decreased, and the slope of the yield versus *E*_impact_ correlation flattened, indicating a partial damping of impact efficiency. At this stage, the liquid probably distributes unevenly, coating particle surfaces and acting as a lubricant that reduces effective energy transfer per collision. At intermediate LAG amounts (*η* = 0.5 µL mg^−1^), yields increased markedly, and the *E*_impact_ correlation steepened again slightly, consistent with more homogeneous wetting and enhanced diffusion at particle interfaces (see Supporting Information for full linear equations). Here, impact‐driven activation and diffusion‐based transport likely act synergistically, marking the *sweet‐spot* in reactivity. At higher *η*‐values (*η* = 1.0 µL mg^−1^), overall yields declined slightly as the system approached slurry‐like conditions, where excess liquid assumingly begins to absorb kinetic energy and reduce the frequency of effective impacts. These results clearly show that LAG modulates, but does not replace, the impact‐driven regime. The existence of an optimal LAG amount arises from a balance between efficient energy transfer and improved molecular mobility, providing a mechanistic rationale for the frequently observed *sweet spot* in LAG‐assisted reactions.

## Conclusion

This study provides an experimentally grounded framework for isolating the individual contributions of single impact energy, cumulative energy, and secondary processes–such as aging and liquid‐assisted grinding (LAG)–to mechanochemical reactivity. By systematically comparing fundamentally different all‐solid reactions under a unified single‐ball milling protocol, we conclude that while all investigated systems are indeed impact‐driven, they respond differently to cumulative energy input and post‐impact dynamics. This allows us to categorize the herein presented mechanochemical transformations into two types that define how mechanical energy translates into chemical reactivity:

- **Reactions completed under mechanical impact**, as represented by the Wittig olefination, in which conversion occurs exclusively during active milling and scales linearly with the cumulative energy (*E*_total_), provided that self‐accelerating or self‐inhibiting effects are absent. Within the defined single‐ball setup, this linear dependency enables precise control of reaction progress via *E*_total_, providing a robust reference system for energy scaling. Such reactions can therefore serve as reliable model transformations to identify, isolate, and refine the individual factors contributing to *E*_total_ in more complex systems, such as multi‐ball planetary mills, resonant acoustic mixers operating under *g*‐forces, or shear‐dominated extrusion systems. By maintaining constant yields under different modes of energy input, these reactions could offer a practical calibration benchmark for translating and comparing energy–reactivity relationships across different mechanochemical devices.
- **Reactions that continue via aging after mechanical activation**, as observed for the Finkelstein halogen‐exchange, in which impact events trigger static transformations that progress even in the absence of further mechanical input. Our findings suggest that the extent and the rate of this post‐impact reactivity correlate directly with the imparted single‐impact energy, indicating that aging is not a passive or undesired side effect but a continuation of the mechanochemical process itself. Recognizing and quantifying this two‐step mechanism–mechanical activation followed by aging–is crucial for accurately interpreting kinetic data and energy dependencies. While aging is often regarded as an unwanted or uncontrolled process, our results highlight its potential as a useful feature in mechanochemical reactions. Controlled exploitation of aging could allow high conversions under milder conditions and reduced energy input, offering new strategies for prolonged, low‐intensity mechanochemical syntheses. In the future, deliberate combination of both processes–direct impact reactivity and subsequent aging–may enable tailored two‐step approaches that exploit both instantaneous and time‐dependent reactivity for optimal efficiency and selectivity.

Furthermore, our results elucidate the effect of liquid‐assisted grinding (LAG) additives. Despite frequent debate, LAG reactions remain firmly impact‐driven, as product yields continue to scale linearly with *E*_impact_ across both reaction types. The presence of small liquid amounts modifies surface properties and influences energy dissipation but does not convert the transformation into a solution‐like process. Depending on the liquid content, LAG can either dampen effective impacts through lubrication or promote reactivity by enhancing diffusion at particle interfaces.

Although mechanochemical reactions can follow different pathways, we propose they are unified by their dependence on the single impact energy. Recognizing the duality between *impact‐complete* and *impact‐triggered* (aging) reactions, and understanding how parameters such as *E*_impact_, *E*_total_, and surface chemistry interact, will allow the field to progress from empirical optimization to mechanistically guided control of all‐solid reactivity. By isolating and quantifying individual contributing factors to mechanochemical reactivity, this study establishes a general experimental framework for kinetic energy investigations in ball‐milling reactions. The approach enables the evaluation of aging and other secondary effects, and precise correlation of energy input with chemical conversion. This marks an important step toward predictive mechanochemistry and hopefully advances both the understanding of established systems and the rational design of new transformations.

## Supporting Information

The authors have cited additional references within the Supporting Information.^[^ ^56^, ^57^ ^]^

## Conflict of Interests

The authors declare no conflict of interest.

## Supporting information



Supporting Information

## Data Availability

The data that support the findings of this study are available in the Supporting Information of this article.

## References

[1] K. R. Floyd , L. S. Mella , R. W. Kwok , M. Gray , E. J. Broker , M. Marianski , T. Friščić , J. D. Batteas , Chem. Sci. 2025, 16, 19830–19842, 10.1039/D5SC04099J. 41019653 PMC12464735

[2] M. Mayer , M. Wohlgemuth , A. Salomé Straub , S. Grätz , L. Borchardt , Angew. Chem. Int. Ed. 2025, 64, e202424139, 10.1002/anie.202424139. 39912800

[3] W. Pickhardt , C. Beaković , M. Mayer , M. Wohlgemuth , F. J. L. Kraus , M. Etter , S. Grätz , L. Borchardt , Angew. Chem. Int. Ed. 2022, 61, e202205003, 10.1002/anie.202205003. PMC9543434 35638133

[4] W. Pickhardt , E. Siegfried , S. Fabig , M. F. Rappen , M. Etter , M. Wohlgemuth , S. Grätz , L. Borchardt , Angew. Chem. Int. Ed. 2023, 62, e202301490, 10.1002/anie.202301490. 37018656

[5] M. Provost , J. Tanepau , T. Buffeteau , M. Gressier , F. Lamaty , J. Pinaud , X. Bantreil , M.‐J. Menu , S. Duluard , Breaking New Ground in Direct Mechanocatalysis: Knoevenagel Condensation via Supported Organo‐catalysts on Zirconia 2025. ChemRxiv, DOI: 10.26434/chemrxiv-2025-0zstm

[6] S. Shah , M. Mokhtar , T. Tran , K. R. Floyd , L. Mella , T. Dao , A. Garza , J. D. Batteas , J. Mack , RSC mechanochem. 2025.

[7] J. Templ , S. Hwang , T. Schwemin , H. Baltaci , L. Borchardt , RSC mechanochem. 2025, 2, 598–602, 10.1039/D5MR00032G. 40415883 PMC12100728

[8] M. Wohlgemuth , S. Schmidt , M. Mayer , W. Pickhardt , S. Graetz , L. Borchardt , Angew. Chem. Int. Ed. 2024, 63, e202405342, 10.1002/anie.202405342. 38801736

[9] P. Brandão , M. Pineiro , in Sustainable Approaches in Pharmaceutical Sciences, John Wiley & Sons, Hoboken, New Jersey, USA, 2023, pp. 255–272, 10.1002/9781119889878.

[10] M. Pérez‐Venegas , E. Juaristi , ACS Sustainable Chem. Eng. 2020, 8, 8881–8893, 10.1021/acssuschemeng.0c01645.

[11] D. Tan , L. Loots , T. Friščić , Chem. Commun. 2016, 52, 7760–7781, 10.1039/C6CC02015A. 27185190

[12] J. Templ , L. Borchardt , Angew. Chem. Int. Ed. 2025, 64, e202503061. 10.1002/anie.202503061 PMC12501704 40326717

[13] J. Templ , M. Schnürch , Angew. Chem. Int. Ed. 2024, 63, e202314637, 10.1002/anie.202314637. PMC10952285 37931225

[14] S. Aydonat , A. H. Hergesell , C. L. Seitzinger , R. Lennarz , G. Chang , C. Sievers , J. Meisner , I. Vollmer , R. Göstl , Polym. J. 2024, 56, 249–268, 10.1038/s41428-023-00863-9.

[15] A. Krusenbaum , S. Grätz , G. T. Tigineh , L. Borchardt , J. G. Kim , Chem. Soc. Rev. 2022, 51, 2873–2905, 10.1039/D1CS01093J. 35302564 PMC8978534

[16] H. W. Lee , K. Yoo , L. Borchardt , J. G. Kim , Green Chem. 2024, 26, 2087–2093.

[17] L. Yang , Z. Chen , C. A. Goult , T. Schlatzer , R. S. Paton , V. Gouverneur , Nature 2025, 640, 100–106. 40140572 10.1038/s41586-025-08698-5 PMC11964924

[18] J. Zhou , T.‐G. Hsu , J. Wang , Angew. Chem. Int. Ed. 2023, 62, e202300768, 10.1002/anie.202300768. 37002927

[19] P. Y. Butyagin , Russ. Chem. Rev. 1971, 40, 901–915, 10.1070/RC1971v040n11ABEH001982.

[20] P. Y. Butyagin , Russ. Chem. Rev. 1984, 53, 1025–1038, 10.1070/RC1984v053n11ABEH003138.

[21] P. Y. Butyagin , I. Pavlichev , React. Solids 1986, 1, 361–372.

[22] O. Lapshin , E. Boldyreva , V. Boldyrev , Russ. J. Inorg. Chem. 2021, 66, 433–453, 10.1134/S0036023621030116.

[23] A. A. Michalchuk , E. V. Boldyreva , A. M. Belenguer , F. Emmerling , V. V. Boldyrev , Front. Chem. 2021, 9, 685789, 10.3389/fchem.2021.685789. 34164379 PMC8216082

[24] P.‐A. Thiessen , K. Meyer , G. Heinicke , Grundlagen der tribochemie De Gruyter, Berlin, Germany, 1967, 10.1515/9783112649022.

[25] G. Cagnetta , J. Huang , B. Wang , S. Deng , G. Yu , Chem. Eng. J. 2016, 291, 30–38, 10.1016/j.cej.2016.01.079.

[26] M. Carta , L. Vugrin , G. Miletić , M. J. Kulcsár , P. C. Ricci , I. Halasz , F. Delogu , Angew. Chem. Int. Ed. 2023, 62, e202308046, 10.1002/anie.202308046. 37377246

[27] F. Delogu , G. Mulas , L. Schiffini , G. Cocco , Mater. Sci. Eng., A 2004, 382, 280–287, 10.1016/j.msea.2004.05.047.

[28] H. Huang , J. Pan , P. G. McCormick , Mater. Sci. Eng., A 1997, 232, 55–62, 10.1016/S0921-5093(97)00084-1.

[29] E. Nwoye , K. Floyd , J. Batteas , J. Felts , RSC Mechanochem. 2025, 2, 911‐922, 10.1039/D5MR00059A.

[30] R. Schmidt , C. F. Burmeister , M. Baláž , A. Kwade , A. Stolle , Org. Process Res. Dev. 2015, 19, 427–436, 10.1021/op5003787.

[31] L. Vugrin , M. Carta , S. Lukin , E. Meštrović , F. Delogu , I. Halasz , Faraday Discuss. 2023, 241, 217–229, 10.1039/D2FD00083K. 36149388

[32] O. F. Jafter , S. Lee , J. Park , C. Cabanetos , D. Lungerich , Angew. Chem. Int. Ed. 2024, 63, e202409731, 10.1002/anie.202409731. 39148147

[33] G. I. Peterson , Angew. Chem. Int. Ed. 2025, 64, e202512324, 10.1002/anie.202512324. 40988371

[34] M. Wohlgemuth , S. Schmidt , M. Mayer , W. Pickhardt , S. Grätz , L. Borchardt , Chem. Eur. J. 2023, 29, e202301714. 37503657 10.1002/chem.202301714

[35] Q. Cao , J. L. Howard , D. E. Crawford , S. L. James , D. L. Browne , Green Chem. 2018, 20, 4443–4447, 10.1039/C8GC02036A.

[36] A. H. Hergesell , C. L. Seitzinger , J. Burg , R. J. Baarslag , I. Vollmer , RSC Mechanochem. 2025, 2, 263–272, 10.1039/D4MR00098F. 39760087 PMC11696860

[37] E. S. Go , E. J. Hong , J. Y. Lee , T. Stolar , G. I. Peterson , F. L. Emmerling , K. Kim , J. G. Kim , JACS Au 2025, 5, 2720–2727, 10.1021/jacsau.5c00322. 40575324 PMC12188479

[38] A. M. Belenguer , A. A. L. Michalchuk , G. I. Lampronti , J. K. M. Sanders , Beilstein J. Org. Chem. 2019, 15, 1226–1235, 10.3762/bjoc.15.120. 31293670 PMC6604707

[39] R. A. Hernandez R , N. Nabavi , S. J. Patterson , P. Forgione , ChemCatChem 2024, 16, e202400406.

[40] A. A. L. Michalchuk , I. A. Tumanov , E. V. Boldyreva , CrystEngComm 2019, 21, 2174–2179, 10.1039/C8CE02109K.

[41] F. Hammerer , L. Loots , J.‐L. Do , J. P. D. Therien , C. W. Nickels , T. Friščić , K. Auclair , Angew. Chem. Int. Ed. 2018, 57, 2621–2624, 10.1002/anie.201711643. 29342316

[42] I. Huskić , C. B. Lennox , T. Friščić , Green Chem. 2020, 22, 5881–5901.

[43] M. Alrbaihat , F. Khalil Al‐Zeidaneen , Q. Abu‐Afifeh , Materials Today: Proceedings 2022, 65, 3651–3656.

[44] J. M. Andersen , J. Mack , Chem. Sci. 2017, 8, 5447–5453, 10.1039/C7SC00538E. 28970924 PMC5609516

[45] P. Baláž , M. Achimovičová , M. Baláž , P. Billik , Z. Cherkezova‐Zheleva , J. M. Criado , F. Delogu , E. Dutková , E. Gaffet , F. J. Gotor , R. Kumar , I. Mitov , T. Rojac , M. Senna , A. Streletskii , K. Wieczorek‐Ciurowa , Chem. Soc. Rev. 2013, 42, 7571–7637. 23558752 10.1039/c3cs35468g

[46] M. Carta , E. Colacino , F. Delogu , A. Porcheddu , Phys. Chem. Chem. Phys. 2020, 22, 14489–14502, 10.1039/D0CP01658F. 32573582

[47] M. Carta , F. Delogu , A. Porcheddu , Phys. Chem. Chem. Phys. 2021, 23, 14178–14194, 10.1039/D1CP01361K. 34132305

[48] E. Colacino , M. Carta , G. Pia , A. Porcheddu , P. C. Ricci , F. Delogu , ACS Omega 2018, 3, 9196–9209. 31459054 10.1021/acsomega.8b01431 PMC6644374

[49] K. Užarević , I. Halasz , T. Friščić , J. Phys. Chem. Lett. 2015, 6, 4129–4140. 26722788 10.1021/acs.jpclett.5b01837

[50] N. Blanc , C. Mayer‐Laigle , X. Frank , F. Radjai , J.‐Y. Delenne , Powder Technol. 2020, 376, 661–667, 10.1016/j.powtec.2020.08.048.

[51] P. Karinkanta , M. Illikainen , J. Niinimäki , Powder Technol. 2013, 233, 286–294, 10.1016/j.powtec.2012.09.011.

[52] M. Rappen , J. Maeder , S. Graetz , L. Borchardt , RSC Mechanochem. 2026, Advance Article, 10.1039/D5MR00112A. PMC12863241 41635527

[53] G. Traversari , A. Porcheddu , G. Pia , F. Delogu , A. Cincotti , Phys. Chem. Chem. Phys. 2021, 23, 229–245, 10.1039/D0CP05647B. 33325477

[54] F. Mele , N. Biedermann , C. Suster , J. Templ , C. Stanetty , M. Schnürch , ChemSusChem 2025, 19, e202502026, 10.1002/cssc.202502026. 41400218 PMC12767755

[55] J. Templ , M. Schnürch , Angew. Chem. Int. Ed. 2024, 63, e202411536, 10.1002/anie.202411536. 39207262

[56] A. Shaabani , P. Mirzaei , S. Naderi , D. G. Lee , Tetrahedron 2004, 60, 11415–11420, 10.1016/j.tet.2004.09.087.

[57] D. Tan , T. Friščić , Eur. J. Org. Chem. 2018, 2018, 18–33, 10.1002/ejoc.201700961.

