# Supplementary material for: Experimental Isolation of Single Impact, Cumulative Energy, Aging, and Liquid‐Assisted Grinding Effects in Mechanochemical Reactions
Source: Angew Chem Int Ed Engl. 2026 Jan 15;65(8):e23191. doi: 10.1002/anie.202523191 (PMC12910137; doi:10.1002/anie.202523191)
Supplement: Supplementary file 1 — Supporting Information [file ANIE-65-e23191-s001.pdf]

Experimental Isolation of Single Impact, Cumulative Energy, Aging, and Liquid-Assisted Grinding Effects in Mechanochemical Reactions

Johanna Templ*[a] and Lars Borchardt*[a]

Supporting Information

Contents

1. General Remarks **9**

2. The Halogen Exchange Reaction (Finkelstein Reaction) **9**

2.1 General Procedure A: **9**

2.2 Reaction Validation and Preliminary Studies: **10**

Control A - Excluding Solvent-induced Reaction Without Milling or Stirring 10

Control B – Exclude Further Reaction Within the NMR Tube: 10

Control C – Aging Experiments: 10

Control D – Exclude Solvent-Induced Reaction after Milling 11

Control E – Drying Experiments 11

Control F – Mechanical Impact as the Major Driving Force 12

Control G - KI vs. NaI, Additives, and Preliminary LAG Studies 12

Control H – Pre-activation of single reaction components 12

2.3 Quantitative NMR Analysis **13**

2.4 Complete Results of Kinetic Studies **14**

2.4.1 Milling Time-Dependent Yield at Defined Milling Frequencies 14

2.4.2 Yield at a Calculated E_{total} of 12 000 J at Selected Frequencies *via* Milling Time Variation 22

2.4.3 Yields at a Constant Milling Time of 12.5 min and Constant Frequency of 20 Hz Using Milling Balls of Different Materials and Masses (15 mm Diameter) 23

2.4.4 Yields at constant impact energy (E_{impact}) via ball mass and frequency variation at constant milling times 26

2.4.5 Yields at Comparable E_{impact} via Frequency Adjustment for Ball Milling Masses with Total Energy (E_{total}) Equalized by Adjusting Milling Times to Standardize the Number of Theoretical Impacts 30

2.4.6 Effects of Different Liquid-Assisted Grinding (LAG) Agents on the Yields at a Constant Milling Time of 12.5 min and Constant Frequency of 20 Hz Using Milling Balls of Different Materials and Masses (15 mm Diameter Each) 35

3. The $KMnO_4$ oxidation **40**

3.1 General Procedure B **40**

3.2 Reaction Validation and Preliminary Studies **40**

Control I – Reaction progress with and without quencher under solvent-free conditions and in solution 40

Control J – Verification of instantaneous and complete oxidant quenching 41

Control K – Exclusion of further reaction within the NMR tube 42

Control L – Check product stability in the presence of oxidant 42

3.3 Quantitative NMR Analysis **42**

3.4 Complete results of kinetic studies **43**

3.4.1 Yields at a Constant Milling Time of 5 min and Constant Frequency of 20 Hz Using Milling Balls of Different Materials and Masses (15 mm Diameter) ... 43

3.4.2 Yields at constant impact energy (E_{impact}) via ball mass and frequency variation at constant milling times ... 45

3.4.3 Yields at Comparable E_{impact} via Frequency Adjustment for Ball Milling Masses with Total Energy (E_{total}) Equalized by Adjusting Milling Times to Standardize the Number of Theoretical Impacts 46

3.4.4 Moderation of the oxidation reaction by MnO_2 ... 48

4. The Wittig Olefination under All-Solid and LAG Conditions ... 49

4.1 General Procedure C ... 50

4.2 Reaction validation and preliminary studies ... 50

Control N – Check the necessity of quenching the base and exclude reaction progress for LAG reactions upon $CDCl_3$ addition ... 50

Control O – Exclude conversion without mechanical impact ... 51

Control P – Aging experiments ... 51

4.3 Quantitative NMR analysis ... 52

4.4 Complete results of kinetic studies ... 53

4.4.1 Milling Time-Dependent Yield at Defined Milling Frequencies ... 53

4.4.2 Yield at a Calculated E_{total} of 12000 J and 18360 J at Selected Frequencies *via* Milling Time Variation ... 55

No LAG, E_{total} = 12 000 J ... 55

21 µL ^{t}BuOH as LAG, E_{total} = 18 360 J ... 56

4.4.3 Yields at constant milling time and frequency using 15 mm milling balls of different materials and masses under all-solid conditions with variable amounts of LAG additives ... 58

No LAG ... 58

21 µL ^{t}BuOH as LAG (η = 0.1) ... 59

107 µL ^{t}BuOH as LAG (η = 0.5) ... 61

213 µL ^{t}BuOH as LAG (η = 1.0) ... 62

4.4.4 Yields at constant impact energy (E_{impact}) via ball mass and frequency variation at constant milling times ... 64

4.4.5 Yields at Comparable E_{impact} via Frequency Adjustment for Ball Milling Masses with Total Energy (E_{total}) Equalized by Adjusting Milling Times to Standardize the Number of Theoretical Impacts 65

5. References ... 67

List of Tables

Table S-1. Reaction parameters and yields of **2** for various reaction with and without aging.

Table S-2. Milling time-dependent yields of product **2** using a single 15 mm zirconia milling ball at the specified frequency.

Table S-3. Average yields of **2** from triplicate reactions as a function of the milling time and the calculated total energies (E_{total}) for each reaction set, grouped by milling frequencies.

Table S-4. Reaction parameters and yields of **2** for reactions having a calculated E_{total} value of 12 000 J at specified milling frequencies, achieved *via* variation of milling time

Table S-5. Reaction parameters and yields of **2** for reactions ball milled for a fixed time of 12.5 min (*non-stop*) at 20 Hz using 15 mm milling balls of different materials.

Table S-6. Reaction parameters and yields of **2** for reactions ball-milled at 20 Hz using 15 mm milling balls of different materials for a fixed total reaction time of 12.5 min (entries 1–16) or 5 min (entries 17–32). Milling was performed in consecutive cycles of 1 min active milling followed by a 10 min break without mechanical impact; active milling times (excluding breaks) are reported in the table

Table S-7. Reaction parameters and yields of **2** for reactions ball milled for a fixed duration of 12.5 min, with milling frequencies adjusted according to the milling ball mass to maintain the single-impact energy (E_{impact}) within a narrow range of 0.079 – 0.087 J.

Table S-8. Reaction parameters and yields of **2** for reactions ball milled for a fixed duration of 7.5 min, with milling frequencies adjusted according to the milling ball mass to maintain the single-impact energy (E_{impact}) within a narrow range of 0.089 – 0.097 J

Table S-9. Reaction parameters and yields of **2** for reactions ball milled for a fixed duration of 10 min, with milling frequencies adjusted according to the milling ball mass to maintain the single-impact energy (E_{impact}) within a narrow range of 0.116 – 0.119 J.

Table S-10. Reaction parameters and yields of **2** for reactions with comparable E_{impact} and E_{total}. Reference experiments are indicated in entries 10–12 (green); for all other entries, the milling times were adjusted to equalize the number of impacts relative to the reference experiments

Table S-11. Reaction parameters and yields of **2** for reactions with comparable E_{impact} and E_{total}. Reference experiments are indicated in entries 7-9 (green); for all other entries, the milling times were adjusted to equalize the number of impacts relative to the reference experiments

Table S-12. Reaction parameters and yields of **2** for reactions with comparable E_{impact} and E_{total}. Reference experiments are indicated in entries 10–12 (green); for all other entries, the milling times were adjusted to equalize the number of impacts relative to the reference experiments.

Table S-13. Reaction parameters and yields of **2** for reactions ball-milled for 12.5 min at a constant frequency of 20 Hz using 15 mm milling balls of different materials, without the addition of a LAG agent.

Table S-14. Reaction parameters and yields of **2** for reactions ball-milled for 12.5 min at a constant frequency of 20 Hz using 15 mm milling balls of different materials, with 30 µL D_2O as LAG.

Table S-15. Reaction parameters and yields of **2** for reactions ball-milled for 12.5 min at a constant frequency of 20 Hz using 15 mm milling balls of different materials, with 30 µL $CDCl_3$ as LAG

Table S-16. Reaction parameters and yields of **2** for reactions ball-milled for 12.5 min at a constant frequency of 20 Hz using 15 mm milling balls of different materials, with 30 µL acetone-d_6 as LAG.

Table S-17. Reaction parameters and yields of **6** for reactions ball-milled for a fixed reaction time of 5 min at a constant milling frequency of 20 Hz using 15 mm milling balls of different materials.

Table S-18. Reaction parameters and yields of **6** for reactions ball milled for a fixed reaction time of 5 min with different frequencies adapted to the milling ball mass to maintain the single impact energy (E_{impact}) within a narrow range of 0.116–0.119 J.

Table S-19. Reaction parameters and yields of **6** for reactions with comparable E_{impact} and E_{total}. Reference experiments are shown in entries 10–12 (green); for all other entries, the milling times were adjusted to equalize the number of impacts relative to the reference experiment.

Table S-20. Control-M experiments demonstrating the effect of MnO_2 on the oxidation reaction yields.

Table S-21. Reaction parameters and yields of **4** for the aging-experiments.

Table S-23. Milling time-dependent yields of product **4** using a single 15 mm zirconia milling ball at the specified frequency.

Table S-23. Average yields of **4** from triplicate reactions as a function of the milling time and the calculated total energies (E_{total}) for each reaction set, grouped by milling frequencies.

Table S-24. Reaction parameters and yields of **4** for reactions with a calculated E_{total} of 12 000 J under all-solid conditions at the specified milling frequencies, with milling times adjusted accordingly.

Table S-25. Reaction parameters and yields of **4** for reactions with a calculated E_{total} of 18 360 J under LAG conditions (21 µL ^{t}BuOH) at the specified milling frequencies, with milling times adjusted accordingly.

Table S-26. Reaction parameters and yields of **4** for reactions ball milled for a fixed time of 10 min at a constant frequency of 25 Hz using 15 mm milling balls of different materials under solvent-free conditions (no LAG added).

Table S-27. Reaction parameters and yields of **4** for reactions ball milled for a fixed time of 10 min at a constant frequency of 25 Hz using 15 mm milling balls of different materials with 21 µL ^{t}BuOH (η = 0.1) as LAG additive.

Table S-28. Reaction parameters and yields of **4** for reactions ball milled for a fixed time of 10 min at a constant frequency of 30 Hz using 15 mm milling balls of different materials with 21 µL ^{t}BuOH (η = 0.1) as LAG additive.

Table S-29. Reaction parameters and yields of **4** for reactions ball milled for a fixed time of 10 min at a constant frequency of 25 Hz using 15 mm milling balls of different materials with 107 µL ^{t}BuOH (η = 0.5) as LAG additive.

Table S-30. Reaction parameters and yields of **4** for reactions ball milled for a fixed time of 10 min at a constant frequency of 25 Hz using 15 mm milling balls of different materials with 213 µL ^{t}BuOH (η = 1.0) as LAG additive.

Table S-31. Reaction parameters and yields of **4** for reactions ball milled for a fixed reaction time of 10 min, with milling frequencies adjusted according to the milling ball mass to maintain the single impact energy (E_{impact}) within a narrow range of 0.116–0.119 J.

Table S-32. Reaction parameters and yields of **4** for reactions with comparable E_{impact} and E_{total}. Reference experiments (entries 10–12, green); for all other entries, milling times were adjusted to match the number of impacts to the reference experiment.

List of Charts

Chart S-1. Average yields of **2** from triplicate reactions as a function of milling time

Chart S-2. Average yields of **2** from triplicate reaction as a function of milling time, plotted against the calculated total energy (E_{total}= 0-80 000)

Chart S-3. Average yields of **2** from triplicate reaction as a function of milling time, plotted against the calculated total energy (E_{total}= 5 000-25 000)

Chart S-4. Yields for specified milling frequencies for a constant, theoretical E_{total} of 12 000 J.

Chart S-5. Yields of **2** as a function of E_{impact} [J] for reactions performed by ball milling for a fixed time of 12.5 min (*non-stop*) at 20 Hz using 15 mm milling balls made from different materials. Black crosses indicate the average yield for each set of reactions conducted with identical ball material density, and the trendline represents the linear correlation of these averages.

Chart S-6. Yields of **2** as a function of E_{impact} [J] for reactions ball-milled at 20 Hz using 15 mm milling balls of different materials for a fixed total reaction time of 12.5 min (**A**) or 5 min (**B**). Milling was performed in consecutive cycles of 1 min active milling followed by a 10 min break without mechanical impact. Black crosses indicate the average yield for each set of reactions conducted with identical ball material density, and the trendlines represent the linear correlation of these averages.

Chart S-7. Yields of **2** for reactions ball milled for a fixed duration of 12.5 min, with milling frequencies adjusted according to the milling ball mass to maintain the single-impact energy (E_{impact}) within a narrow range of 0.079 – 0.087 J.

Chart S-8. Yields of **2** for reactions ball milled for a fixed duration of 7.5 min, with milling frequencies adjusted according to the milling ball mass to maintain the single-impact energy (E_{impact}) within a narrow range of 0.089 – 0.097 J.

Chart S-9. Yields of **2** for reactions ball milled for a fixed duration of 10 min, with milling frequencies adjusted according to the milling ball mass to maintain the single-impact energy (E_{impact}) within a narrow range of 0.116 – 0.119 J.

Chart S-10. Yields of **2** for reactions with comparable E_{impact} and E_{total}. Reference experiments are shown in the fourth box from the left (green); for all other entries, the milling times were adjusted to equalize the number of impacts relative to the reference experiments.

Chart S-11. Yields of **2** for reactions with comparable E_{impact} and E_{total}. Reference experiments are shown in the third box from the left (green); for all other entries, the milling times were adjusted to equalize the number of impacts relative to the reference experiments

Chart S-12. Yields of **2** for reactions with comparable E_{impact} and E_{total}. Reference experiments are shown in the fourth box from the left (green); for all other entries, the milling times were adjusted to equalize the number of impacts relative to the reference experiments

Chart S-13. Yields of **2** as a function of E_{impact} [J] for reactions performed by ball milling for a fixed time of 12.5 min at 20 Hz using 15 mm milling balls made from different materials, without the addition of

a LAG agent. Black crosses indicate the average yield for each set of reactions conducted with identical E_{impact}, and the trendline represents the linear correlation of these averages.

Chart S-14. Yields of **2** as a function of E_{impact} [J] for reactions performed by ball milling for a fixed time of 12.5 min at 20 Hz using 15 mm milling balls made from different materials, with 30 µL D_2O as LAG. Black crosses indicate the average yield for each set of reactions conducted with identical E_{impact}, and the trendline represents the linear correlation of these averages.

Chart S-15. Yields of **2** as a function of E_{impact} [J] for reactions performed by ball milling for a fixed time of 12.5 min at 20 Hz using 15 mm milling balls made from different materials, with 30 µL $CDCl_3$ as LAG. Black crosses indicate the average yield for each set of reactions conducted with identical E_{impact}, and the trendline represents the linear correlation of these averages.

Chart S-16. Yields of **2** as a function of E_{impact} [J] for reactions performed by ball milling for a fixed time of 12.5 min at 20 Hz using 15 mm milling balls made from different materials, with 30 µL acetone-d_6 as LAG. Black crosses indicate the average yield for each set of reactions conducted with identical E_{impact}, and the trendline represents the linear correlation of these averages.

Chart S-17. Yields of **6** as a function of E_{impact} [J] for reactions performed by ball milling for a fixed time of 5 min at 20 Hz using 15 mm milling balls made from different materials. Black crosses indicate the average yield for each set of reactions conducted with identical E_{impact}, and the trendline represents the polynomic (2nd order) correlation of these averages.

Chart S-18. Yields of **6** for reactions ball milled for a fixed reaction time of 5 min with different frequences adapted for the milling ball mass to keep the single impact energy (E_{impact}) in a narrow range of 0.116 – 0.119 J.

Chart S-19. Yields of **6** for reactions with comparable E_{impact} and $E_{total.}$ Reference experiments (silicon nitride, 15 mm) are displayed in the rightmost column (4th box); for all other entries, the milling times were adjusted to equalize the number of impacts relative to the reference experiment.

Chart 20. Average yields of **4** from triplicate reaction as a function of milling time, plotted against the calculated total energy (E_{total}= 5 000-25 000 J)

Chart S-21. Yields of **4** for reactions with a calculated E_{total} of 12 000 J under all-solid conditions at the specified milling frequencies, with milling times adjusted accordingly.

Chart S-22. Yields of **4** for reactions with a calculated E_{total} of 18 360 J under LAG conditions (21 µL ^{t}BuOH) at the specified milling frequencies, with milling times adjusted accordingly.

Chart S-23. Yields of **4** as a function of E_{impact} [J] performed by ball milling for a fixed time of 10 min at 25 Hz using 15 mm milling balls made from different materials, without the addition of a LAG agent. Black crosses indicate the average yield for each set of reactions conducted with identical E_{impact}, and the trendline represents the linear correlation of these averages.

Chart S-24. Yields of **4** as a function of E_{impact} [J] performed by ball milling for a fixed time of 10 min at 25 Hz using 15 mm milling balls made from different materials, with 21 µL ^{t}BuOH (η = 0.1) as LAG additive. Black crosses indicate the average yield for each set of reactions conducted with identical E_{impact}, and the trendline represents the linear correlation of these averages.

Chart S-25. Yields of **4** as a function of E_{impact} [J] performed by ball milling for a fixed time of 10 min at 30 Hz using 15 mm milling balls made from different materials, with 21 µL ^{t}BuOH (η = 0.1) as LAG additive. Black crosses indicate the average yield for each set of reactions conducted with identical E_{impact}, and the trendline represents the linear correlation of these averages.

Chart S-26. Yields of **4** as a function of E_{impact} [J] for reactions performed by ball milling for a fixed time of 10 min at 25 Hz using 15 mm milling balls made from different materials, with 107 µL ^{t}BuOH (η = 0.5) as LAG additive. Black crosses indicate the average yield for each set of reactions conducted at identical E_{impact}, and the trendline represents the linear correlation of these averages.

Chart S-27. Yields of **4** as a function of E_{impact} [J] for reactions performed by ball milling for a fixed time of 10 min at 25 Hz using 15 mm milling balls made from different materials, with 213 µL ^{t}BuOH (η = 1.0) as LAG additive. Black crosses indicate the average yield for each set of reactions conducted at identical E_{impact}, and the trendline represents the linear correlation of these averages.

Chart S-28. Combined results of LAG studies of the Wittig olefination using different amounts of ^{t}BuOH as LAG agent. The datapoints (crosses) correspond to the average yield of triplicate reactions. Yield of **4** is shown as a function of E_{impact} [J].

Chart S-29. Yields of **4** for reactions ball milled for a fixed reaction time of 10 min, with milling frequencies adjusted according to the milling ball mass to maintain the single impact energy (E_{impact}) within a narrow range of 0.116–0.119 J.

Chart S-30. Yields of **4** for reactions with comparable E_{impact} and E_{total}. Reference experiments (silicon nitride, 15 mm) are displayed in the rightmost column (4th box); for all other entries, milling times were adjusted to match the number of impacts to the reference experiment.

1. General Remarks

General. All reagents were obtained from commercial suppliers at least in synthesis grade purity and were used without further purification. Organic solvents used for LAG were obtained in analysis grade. The milling was carried out exclusively in a Retsch MM-500 ball mill. The milling vessels were custom made from PFA.

^{1}H NMR spectra were recorded on a Bruker Avance III HD spectrometer at 400 MHz at ambient temperature. Chemical Shifts (δ) are reported in ppm.

Milling Media. Polymer material for manufacturing of PFA Vessels were obtained from Kelux in Geldern, Germany. Milling balls out of different material were purchased from Kugel-Winnie, Germany.

Temperature Measurements. The temperature was measured after milling at the inner vessel wall using a commercially available handheld infrared thermometer.

2. The Halogen Exchange Reaction (Finkelstein Reaction)

The Finkelstein reaction, a halogen exchange process, was deliberately selected to study purely all-solid mechanochemical conversions. All reagents are solid, and the reaction produces no liquid byproducts—only NaBr as a solid salt.

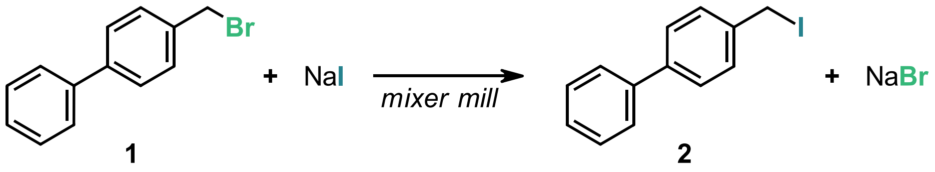

Scheme S-1. The Finkelstein reaction, a halogen exchange reaction

2.1 General Procedure A:

A 14 mL PFA vessel containing a single milling ball was charged with 4-(Bromomethyl)-1,1'-biphenyl **(1)** [CAS: 2567-29-5] (62 mg, 0.25 mmol, 1 equiv.) and sodium iodide [CAS: 7681-82-5] (94 mg, 0.5 mmol, 2 equiv.). The vessel was closed and mounted into a Retsch MM500 vario. Milling was carried out for the specified time and frequency as given in the respective table entries. Ball size and weight are likewise specified in the tables. Unless stated otherwise, no liquid-assisted grinding (LAG) agents were added. Where LAG was applied, the type and volume are reported in the dedicated sections.

Immediately after milling, the vessel wall temperature was measured by infrared thermometer. After cooling to room temperature, 0.8 mL $CDCl_3$ was added, followed by either 50 µL of a 1 M benzyl benzoate [CAS: 120-51-4] solution in $CDCl_3$ (*via* pipette or microsyringe) or direct addition of the internal standard by microsyringe. The vessel walls were rinsed carefully with solvent, and the mixture was filtered through a short celite plug (cotton-stuffed Pasteur pipette) directly into an NMR tube. ^{1}H-NMR spectra were recorded with a prolonged relaxation delay (D1) of 5 seconds.

For post-acquisition FID processing see **Section 2.3**.

2.2 Reaction Validation and Preliminary Studies:

To investigate the kinetics and mechanical effects of the all-solid Finkelstein reaction, it was essential to confirm that no significant conversion occurs during solvent addition and subsequent NMR quantification. Several control experiments were therefore carried out as detailed below.

Control A - Excluding Solvent-induced Reaction Without Milling or Stirring

Control A1 and A2: Two glass vials were charged with each 4-(Bromomethyl)-1,1'-biphenyl **(1)** (62 mg, 0.25 mmol, 1 equiv.) and sodium iodide (94 mg, 0.5 mmol, 2 equiv.), followed by addition of 1 mL $CDCl_3$ each.

> **Control A1**: The suspension was immediately filtered over celite into an NMR tube and analyzed by ^{1}H-NMR.
>
> **Control A2**: The mixture was left to stand for 15 min before celite filtration and NMR analysis.
>
> For both **control A1** and **A2** only unreacted starting material **1** was observed; **no product (2) was detected.**

Thus, $CDCl_3$ addition and the sample preparation procedure described in General Procedure A do not induce reaction and can be used to quantify only the ball-milled conversion.

Control A3: A glass vials were charged with 4-(Bromomethyl)-1,1'-biphenyl **(1)** (62 mg, 0.25 mmol, 1 equiv.), sodium iodide (94 mg, 0.5 mmol, 2 equiv.) and **30 µL acetone-d_6**, followed by addition of 1 mL $CDCl_3$ and the internal NMR standard. The suspension was mixed for approximately 1 min, filtered over celite into an NMR tube, and analyzed.

> **Control A3** showed 71% conversion, with product **2** obtained in **26% yield**.

This result clearly indicates that while no conversion is detected without milling upon $CDCl_3$ addition in no-LAG experiments or when using $D_2O/CDCl_3$ as LAG agents, the use of acetone-d_6 as LAG leads to rapid reaction even in the absence of mechanical impact before and once $CDCl_3$ is added. Therefore, results from LAG experiments with acetone-d_6, a solvent commonly employed in solution-phase Finkelstein reactions, do not strictly reflect the yields obtained under purely mechanochemical conditions and vary depending on the time taken for NMR sample preparation.

Control B – Exclude Further Reaction Within the NMR Tube:

A reaction was performed according to General Procedure A with a single 15 mm tungsten carbide ball (26.226 g), 20 Hz, 12.5 min. After workup and addition of the internal standard, an immediate NMR was taken, and the same sample was re-measured after 20 h.

No changes in substrate/product ratio were observed, confirming that no further conversion occurs in $CDCl_3$ after sample preparation.

Control C – Aging Experiments:

To examine post-milling aging in the all-solid reaction mixture, pairs of identical reactions were run (General Procedure A, 15 mm ZrO_2 ball). One sample was prepared and measured immediately; the other was left standing without solvent or mechanical impact for a defined time before NMR analysis. Reaction parameters and yields are given in **Table S-1**.

*The results show that without solvent or further mechanical energy, the reaction progresses only slowly. Thus, fast sample preparation accurately reflects the yield of **2** generated under ball milling conditions.*

Table S-1. Reaction parameters and yields of **2** for various reaction with and without aging.

entry	frequency [Hz]	milling time [min]	aging time [h]	recovery (1)	yield (2)	average yield (2)	Δ yield (2) with aging
1	20	10	-	60%	39%		-
2	20	10	-	62%	36%	40 ± 4%	-
3	20	10	-	55%	44%		-
4	20	10	**12**	55%	44%		**4%**
5	20	10	**12**	55%	43%	44 ± 1%	**3%**
6	20	10	**12**	53%	46%		**6%**
7	30	2.5	-	81%	13%		-
8	30	2.5	-	84%	14%	13 ± 1%	-
9	30	2.5	-	88%	12%		-
10	30	2.5	**0.17**	76%	16%	-	**3%**
11	30	2.5	**1**	77%	21%	-	**8%**
12	30	2.5	**2**	77%	22%	-	**9%**
13	30	2.5	**18**	69%	29%	-	**16%**
14	30	2.5	**24**	66%	30%	-	**17%**

Control D – Exclude Solvent-Induced Reaction after Milling

Two identical reactions were conducted according to General Procedure A using a single 10 mm zirconia milling ball each (ball masses = 3.143 g and 3.151 g), at 35 Hz for 10 min. After milling, one reaction (**Control D1**) was immediately worked up and subjected to quantitative ^{1}H NMR analysis. For the other reaction (**Control D2**), 1 mL $CDCl_3$ was added to the milling vessel, and the mixture was left to stand without stirring or further mechanical impact for 1.5 h before NMR analysis. **Control D1** gave 32% yield of iodinated product **2**, while **Control D2** gave 40%, corresponding to an 8% difference. This yield increase is in the same range as the differences observed in solvent-free "aging" experiments (cf. **Table S-1, entries 11** and **12**). These results again highlight that fast and consistent sample preparation followed by quantitative NMR analysis reflects the yield generated during the actual ball-milling reaction.

Control E – Drying Experiments

To assess whether adsorbed water affects the reaction outcome, considering the strongly hygroscopic nature of sodium iodide, several experiments were carried out in which either sodium iodide alone or both reactants were pre-dried at elevated temperatures. The results were compared with standard, non-dried conditions.

All reactions were conducted under General Procedure A using a single 15 mm ZrO_2 milling ball at 20 Hz for 10 min, with the only variation being the drying procedure:

Control E1: No pre-drying of reagents → **45% yield of 2** (average of 4 runs).

Control E2: Both reagents pre-dried → **49% yield of 2**.

4-(Bromomethyl)-1,1'-biphenyl **(1)** dried at 80 °C for 6 h (ambient pressure).

Sodium iodide dried at 280 °C for 6 h (ambient pressure).

Control E3: Only sodium iodide pre-dried (280 °C, 6 h) → **45% yield of 2**.

*These results show that small amounts of water present in sodium iodide do not significantly influence the outcome of the reaction. Therefore, pre-drying of starting material **1** and NaI was omitted for all subsequent experiments.*

Control F – Mechanical Impact as the Major Driving Force

The following experiments were designed to demonstrate that mechanical forces (shear, impact, friction) are the major driving factors of the reaction, rather than particle translocation of the reagents without mechanically induced regeneration of reactive surfaces.

All reactions were performed under General Procedure A at 30 Hz for 10 min, with varying milling media to reduce impact force while enhancing particle translocation:

Control F1: 1 × 15 mm ZrO_2 ball → **68% yield of 2** (average of 3 runs).

Control F2: No milling balls → **0% yield of 2** (average of 3 runs).

Control F3: 10 × 5 mm polyoxomethylene (POM) balls, total ball weight 0.886 g → **4% yield of 2** (average of 3 runs).

In **Control F3**, reactant translocation should be maximal, but mechanical impact and shear forces are minimized. The very low conversion obtained under these conditions clearly indicates that the reaction is driven by harsh mechanical forces and the resulting activation of reagent surfaces, rather than by efficient but low-energy particle translocation.

Control G - KI vs. NaI, Additives, and Preliminary LAG Studies

As part of the preliminary studies, potassium iodide was compared with sodium iodide as halogen-exchange salts. Reactions were conducted following the General Procedure A at 35 Hz for 15 min using a single 10 mm ZrO_2 ball. NMR spectra were recorded without internal standard; thus, only relative ratios of starting material 1 to product 2 are reported. No side products were detected.

- Sodium iodide (2.5 equiv., 0.75 mmol): 49:51.
- Potassium iodide (2.5 equiv., 0.75 mmol): 17:83 .

In addition, typical additives known to accelerate halogen exchange were tested. Addition of tetrabutylammonium iodide (TBAI, 10 mol%, 0.05 mmol) to a reaction (General Procedure A, 35 Hz, 10 min, 1 × 10 mm ZrO_2 ball) led to full conversion of 1 to 2. Similarly, when acetone was added as a liquid-assisted grinding (LAG) agent (25 µL, η = 0.25) under otherwise identical conditions, complete conversion was also observed.

For the kinetic studies, sodium iodide was chosen as the halogen-exchange reagent throughout, without additional additives, except where LAG agents were explicitly tested.

Control H – Pre-activation of single reaction components

To investigate whether surface activation of individual reaction components influences the overall reaction yield, a series of control reactions was performed. In the first set (**Control H-1** to **H-2**), the reaction was carried out without any pre-milling, following General Procedure A, using a 15 mm ZrO_2 milling ball at 30 Hz for 5 min. In the subsequent sets, one of the two reactants was pre-milled separately: NaI (**Control H-3** to **H-4**) or 4-(Bromomethyl)-1,1'-biphenyl **(1)** (**Control H-5** to **H-6**). Each pre-milling step was conducted in a reaction vessel with a 15 mm ZrO_2 ball at 30 Hz for 5 min, after which the second, non-pre-milled reactant was added, and milling was continued for another 5 min at 30 Hz.

Control H-1 to **H-2**: no pre-milling → average yield of duplicate reactions: **41%**

Control H-3 to **H-4**: NaI pre-milled → average yield of duplicate reactions: **42%**

Control H-5 to **H-6**: 4-(Bromomethyl)-1,1'-biphenyl **(1)** pre-milled → average yield of duplicate reactions: **45%**

These results suggest that surface activation via pre-milling of individual components has little to no effect on the overall yield as yield differences observed lie within the human and instrumental error range.

2.3 Quantitative NMR Analysis

After sample preparation as described in General Procedure A, ^{1}H-NMR spectra were recorded on a Bruker 400 MHz spectrometer with a prolonged relaxation delay (D1) of 5 seconds.

Spectra were processed using MestreNova software with the following parameters:

- Apodization along t_1: exponential, 0.0 Hz.
- Zero filling along t_1: from FID size to 524288 points (512K).
- Automatic phase correction: Global, Whitening, Regions, Selective, Min. Entropy, Metabonomics, and Baseline Optimization (initial phase = 0).
- Automatic baseline correction: ablative method, 5 points, 10 passes.

An example spectrum for quantitative ^{1}H-NMR analysis is shown in **Figure S-1**, displaying only the relevant region and including the internal standard.

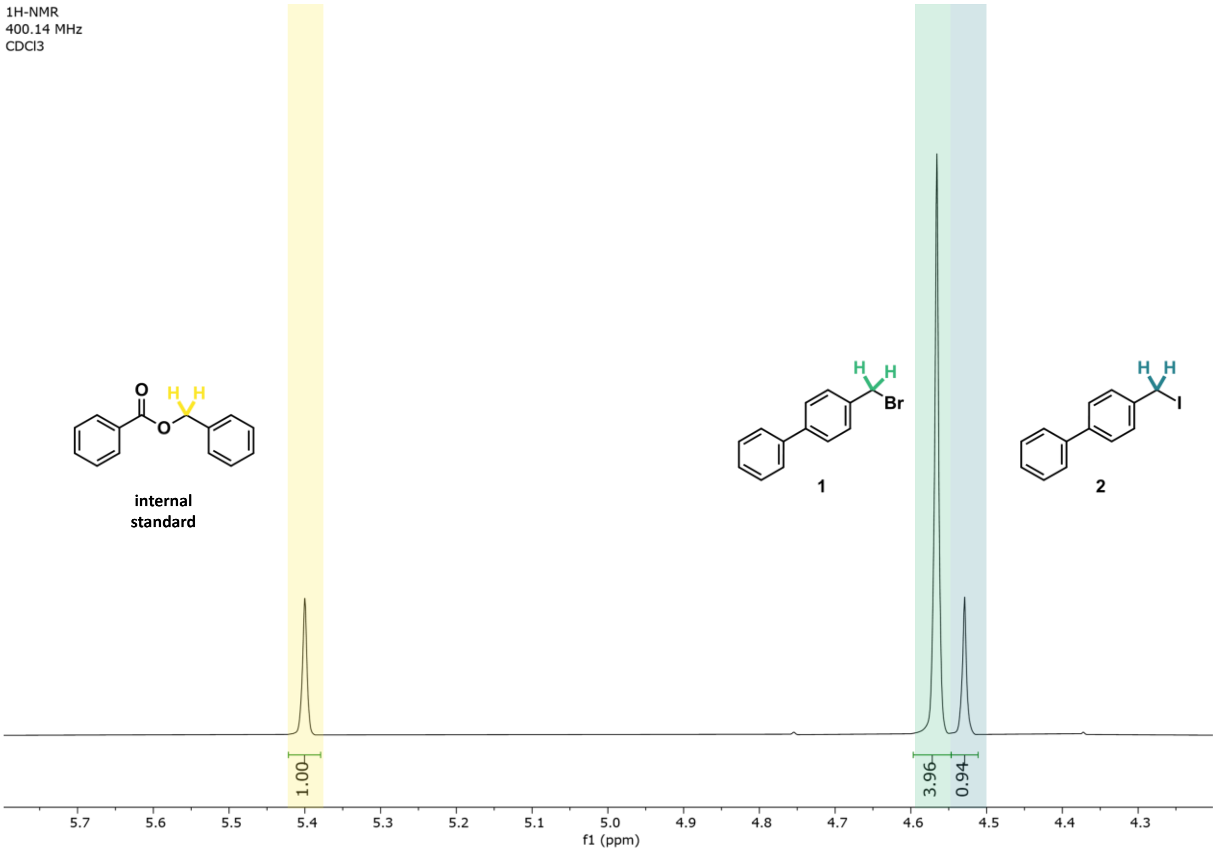

Figure S-1. Example ^{1}H-NMR spectra for quantitative analysis of the halogen exchange reaction including the signal of the internal NMR standard benzyl benzoate.

2.4 Complete Results of Kinetic Studies

In the following, all results are presented in full. Unless stated otherwise, reactions were performed according to General Procedure A. The milling material and ball size, as well as the milling frequency and reaction time, are specified in the respective table entries.

The single impact energy and total energy input for each experiment were calculated following the method described by the Lungerich group [1] using their online ball mill calculator for mixer mills (https://lungerich-group.github.io/Ball-mill-calculator/). Calculations were based on a system with a single milling ball and the following PFA jar dimensions:

- Diameter: 20 mm
- Length: 60 mm
- Oscillation Amplitude: 30 mm

The milling ball size [mm], weight [g], frequency [Hz], and time [min] used for each reaction are given in the respective tables. In all cases where incomplete conversion was observed, only unreacted starting material **1** remained. Quantitative recovery of **1** was confirmed by ^{1}H NMR, and no by-products or decomposition were detected in any experiment.

2.4.1 Milling Time-Dependent Yield at Defined Milling Frequencies

To determine the effect of milling time at fixed frequencies, reactions were conducted following the General Procedure A using a single 15 mm zirconia (ZrO_2) milling ball per jar. The ball mass used for energy calculations was 11.300 g, corresponding to the average mass of 30 randomly selected 15 mm ZrO_2 balls.

The vessel temperature was recorded immediately after milling using an infrared thermometer, and mean values from sets of three identical reactions are reported in the corresponding table.

Table S-2. Milling time-dependent yields of product **2** using a single 15 mm zirconia milling ball at the specified frequency.

entry	ball size [mm]	ball material	material density [g/cm³]	ball mass [g]	milling time [min]	frequency [Hz]	calc. E_{impact} [J]	calc. E_{total} [J]	temperature [°C]	yield (2)	average yield (2) ± std. dev.
					**35 Hz**						
1	15	ZrO_2	6.10	11.300	2.5	35	0.246	12597		24%	
2	15	ZrO_2	6.10	11.300	2.5	35	0.246	12597	34	24%	23 ± 1%
3	15	ZrO_2	6.10	11.300	2.5	35	0.246	12597		23%	
4	15	ZrO_2	6.10	11.300	5	35	0.246	25194		39%	
5	15	ZrO_2	6.10	11.300	5	35	0.246	25194	44	37%	37 ± 2%
6	15	ZrO_2	6.10	11.300	5	35	0.246	25194		35%	
7	15	ZrO_2	6.10	11.300	7.5	35	0.246	37790		51%	
8	15	ZrO_2	6.10	11.300	7.5	35	0.246	37790	51	50%	50 ± 1%
9	15	ZrO_2	6.10	11.300	7.5	35	0.246	37790		49%	
10	15	ZrO_2	6.10	11.300	10	35	0.246	50387		99%	
11	15	ZrO_2	6.10	11.300	10	35	0.246	50387	51	70%	78 ±18%
12	15	ZrO_2	6.10	11.300	10	35	0.246	50387		65%	
13	15	ZrO_2	6.10	11.300	12.5	35	0.246	62984		98%	
14	15	ZrO_2	6.10	11.300	12.5	35	0.246	62984	58	98%	92 ± 9%
15	15	ZrO_2	6.10	11.300	12.5	35	0.246	62984		81%	
16	15	ZrO_2	6.10	11.300	15	35	0.246	75581		99%	
17	15	ZrO_2	6.10	11.300	15	35	0.246	75581	61	91%	96 ± 4%
18	15	ZrO_2	6.10	11.300	15	35	0.246	75581		98%	

entry	ball size [mm]	ball material	material density [g/cm³]	ball mass [g]	milling time [min]	frequency [Hz]	calc. E_{impact} [J]	calc. E_{total} [J]	temperature [°C]	yield (2)	average yield (2) ± std. dev.
					32 Hz						
19	15	ZrO_2	6.10	11.300	2.5	32	0.206	9627		20%	
20	15	ZrO_2	6.10	11.300	2.5	32	0.206	9627	30	21%	18 ± 4%
21	15	ZrO_2	6.10	11.300	2.5	32	0.206	9627		14%	
22	15	ZrO_2	6.10	11.300	5	32	0.206	19255		38%	
23	15	ZrO_2	6.10	11.300	5	32	0.206	19255	38	37%	34 ± 6%
24	15	ZrO_2	6.10	11.300	5	32	0.206	19255		28%	
25	15	ZrO_2	6.10	11.300	7.5	32	0.206	28882		74%	
26	15	ZrO_2	6.10	11.300	7.5	32	0.206	28882	37	64%	61 ± 14%
27	15	ZrO_2	6.10	11.300	7.5	32	0.206	28882		45%	
28	15	ZrO_2	6.10	11.300	10	32	0.206	38509		94%	
29	15	ZrO_2	6.10	11.300	10	32	0.206	38509	46	95%	85 ± 17%
30	15	ZrO_2	6.10	11.300	10	32	0.206	38509		65%	
31	15	ZrO_2	6.10	11.300	12.5	32	0.206	48137		98%	
32	15	ZrO_2	6.10	11.300	12.5	32	0.206	48137	46	99%	92 ± 10%
33	15	ZrO_2	6.10	11.300	12.5	32	0.206	48137		80%	
34	15	ZrO_2	6.10	11.300	15	32	0.206	57764		99%	
35	15	ZrO_2	6.10	11.300	15	32	0.206	57764	50	99%	95 ± 7%
36	15	ZrO_2	6.10	11.300	15	32	0.206	57764		87%	
					30 Hz						
37	15	ZrO_2	6.10	11.300	2.5	30	0.181	7933		14%	
38	15	ZrO_2	6.10	11.300	2.5	30	0.181	7933	28	14%	13 ± 1%
39	15	ZrO_2	6.10	11.300	2.5	30	0.181	7933		12%	
40	15	ZrO_2	6.10	11.300	5	30	0.181	15865		41%	
41	15	ZrO_2	6.10	11.300	5	30	0.181	15865	43	40%	40 ± 2%
42	15	ZrO_2	6.10	11.300	5	30	0.181	15865		38%	
43	15	ZrO_2	6.10	11.300	7.5	30	0.181	23798		59%	
44	15	ZrO_2	6.10	11.300	7.5	30	0.181	23798	40	52%	54 ± 5%
45	15	ZrO_2	6.10	11.300	7.5	30	0.181	23798		50%	
46	15	ZrO_2	6.10	11.300	10	30	0.181	31731		66%	
47	15	ZrO_2	6.10	11.300	10	30	0.181	31731	44	70%	65 ± 5%
48	15	ZrO_2	6.10	11.300	10	30	0.181	31731		60%	
49	15	ZrO_2	6.10	11.300	12.5	30	0.181	39663		90%	
50	15	ZrO_2	6.10	11.300	12.5	30	0.181	39663	48	75%	80 ± 8%
51	15	ZrO_2	6.10	11.300	12.5	30	0.181	39663		76%	
52	15	ZrO_2	6.10	11.300	15	30	0.181	47596		95%	
53	15	ZrO_2	6.10	11.300	15	30	0.181	47596	48	85%	89 ± 5%
54	15	ZrO_2	6.10	11.300	15	30	0.181	47596		87%	

entry	ball size [mm]	ball material	material density [g/cm^3]	ball mass [g]	milling time [min]	frequency [Hz]	calc. E_{impact} [J]	calc. E_{total} [J]	temperature [°C]	yield (2)	average yield (2) ± std. dev.
						**27 Hz**					
55	15	ZrO_2	6.10	11.300	2.5	27	0.146	5783		13%	
56	15	ZrO_2	6.10	11.300	2.5	27	0.146	5783	31	14%	14 ± 2%
57	15	ZrO_2	6.10	11.300	2.5	27	0.146	5783		16%	
58	15	ZrO_2	6.10	11.300	5	27	0.146	11566		29%	
59	15	ZrO_2	6.10	11.300	5	27	0.146	11566	33	28%	27 ± 3%
60	15	ZrO_2	6.10	11.300	5	27	0.146	11566		23%	
61	15	ZrO_2	6.10	11.300	7.5	27	0.146	17349		44%	
62	15	ZrO_2	6.10	11.300	7.5	27	0.146	17349	39	43%	48 ± 7%
63	15	ZrO_2	6.10	11.300	7.5	27	0.146	17349		57%	
64	15	ZrO_2	6.10	11.300	10	27	0.146	23132		64%	
65	15	ZrO_2	6.10	11.300	10	27	0.146	23132	38	67%	67 ± 3%
66	15	ZrO_2	6.10	11.300	10	27	0.146	23132		69%	
67	15	ZrO_2	6.10	11.300	12.5	27	0.146	28915		79%	
68	15	ZrO_2	6.10	11.300	12.5	27	0.146	28915	41	80%	80 ± 1%
69	15	ZrO_2	6.10	11.300	12.5	27	0.146	28915		80%	
70	15	ZrO_2	6.10	11.300	15	27	0.146	34698		88%	
71	15	ZrO_2	6.10	11.300	15	27	0.146	34698	44	86%	86 ± 2%
72	15	ZrO_2	6.10	11.300	15	27	0.146	34698		85%	
						**25 Hz**					
73	15	ZrO_2	6.10	11.300	2.5	25	0.125	4591		11%	
74	15	ZrO_2	6.10	11.300	2.5	25	0.125	4591	31	9%	10 ± 1%
75	15	ZrO_2	6.10	11.300	2.5	25	0.125	4591		10%	
76	15	ZrO_2	6.10	11.300	5	25	0.125	9181		22%	
77	15	ZrO_2	6.10	11.300	5	25	0.125	9181	33	21%	22 ± 2%
78	15	ZrO_2	6.10	11.300	5	25	0.125	9181		24%	
79	15	ZrO_2	6.10	11.300	7.5	25	0.125	13772		36%	
80	15	ZrO_2	6.10	11.300	7.5	25	0.125	13772	36	35%	40 ± 7%
81	15	ZrO_2	6.10	11.300	7.5	25	0.125	13772		48%	
82	15	ZrO_2	6.10	11.300	10	25	0.125	18363		53%	
83	15	ZrO_2	6.10	11.300	10	25	0.125	18363	36	52%	51 ± 2%
84	15	ZrO_2	6.10	11.300	10	25	0.125	18363		49%	
85	15	ZrO_2	6.10	11.300	12.5	25	0.125	22953		69%	
86	15	ZrO_2	6.10	11.300	12.5	25	0.125	22953	38	63%	66 ± 3%
87	15	ZrO_2	6.10	11.300	12.5	25	0.125	22953		65%	
88	15	ZrO_2	6.10	11.300	15	25	0.125	27544		77%	
89	15	ZrO_2	6.10	11.300	15	25	0.125	27544	43	83%	81 ± 4%
90	15	ZrO_2	6.10	11.300	15	25	0.125	27544		84%	

entry	ball size [mm]	ball material	material density [g/cm^3]	ball mass [g]	milling time [min]	frequency [Hz]	calc. E_{impact} [J]	calc. E_{total}[J]	temperature [°C]	yield (2)	average yield (2) ± std. dev.
					22 Hz						
91	15	ZrO_2	6.10	11.300	2.5	22	0.097	3128		6%	
92	15	ZrO_2	6.10	11.300	2.5	22	0.097	3128	28	5%	5 ± 1%
93	15	ZrO_2	6.10	11.300	2.5	22	0.097	3128		4%	
94	15	ZrO_2	6.10	11.300	5	22	0.097	6257		13%	
95	15	ZrO_2	6.10	11.300	5	22	0.097	6257	31	17%	16 ± 2%
96	15	ZrO_2	6.10	11.300	5	22	0.097	6257		17%	
97	15	ZrO_2	6.10	11.300	7.5	22	0.097	9385		36%	
98	15	ZrO_2	6.10	11.300	7.5	22	0.097	9385	32	30%	32 ± 3%
99	15	ZrO_2	6.10	11.300	7.5	22	0.097	9385		31%	
100	15	ZrO_2	6.10	11.300	10	22	0.097	12514		44%	
101	15	ZrO_2	6.10	11.300	10	22	0.097	12514	35	47%	45 ± 1%
102	15	ZrO_2	6.10	11.300	10	22	0.097	12514		45%	
103	15	ZrO_2	6.10	11.300	12.5	22	0.097	15642		50%	
104	15	ZrO_2	6.10	11.300	12.5	22	0.097	15642	35	57%	55 ± 5%
105	15	ZrO_2	6.10	11.300	12.5	22	0.097	15642		59%	
106	15	ZrO_2	6.10	11.300	15	22	0.097	18770		77%	
107	15	ZrO_2	6.10	11.300	15	22	0.097	18770	35	80%	78 ± 2%
108	15	ZrO_2	6.10	11.300	15	22	0.097	18770		76%	
					20 Hz						
109	15	ZrO_2	6.10	11.300	2.5	20	0.080	2350		7%	
110	15	ZrO_2	6.10	11.300	2.5	20	0.080	2350	29	7%	6 ± 2%
111	15	ZrO_2	6.10	11.300	2.5	20	0.080	2350		4%	
112	15	ZrO_2	6.10	11.300	5	20	0.080	4701		15%	
113	15	ZrO_2	6.10	11.300	5	20	0.080	4701	26	17%	19 ± 5%
114	15	ZrO_2	6.10	11.300	5	20	0.080	4701		25%	
115	15	ZrO_2	6.10	11.300	7.5	20	0.080	7051		34%	
116	15	ZrO_2	6.10	11.300	7.5	20	0.080	7051	32	33%	30 ± 6%
117	15	ZrO_2	6.10	11.300	7.5	20	0.080	7051		23%	
118	15	ZrO_2	6.10	11.300	10	20	0.080	9402		45%	
119	15	ZrO_2	6.10	11.300	10	20	0.080	9402	27	44%	45 ± 6%
120	15	ZrO_2	6.10	11.300	10	20	0.080	9402		44%	
121	15	ZrO_2	6.10	11.300	12.5	20	0.080	11752		49%	
122	15	ZrO_2	6.10	11.300	12.5	20	0.080	11752	32	58%	53 ± 4%
123	15	ZrO_2	6.10	11.300	12.5	20	0.080	11752		53%	
124	15	ZrO_2	6.10	11.300	15	20	0.080	14103		67%	
125	15	ZrO_2	6.10	11.300	15	20	0.080	14103	35	69%	65 ± 5%
126	15	ZrO_2	6.10	11.300	15	20	0.080	14103		59%	

entry	ball size [mm]	ball material	material density [g/cm³]	ball mass [g]	milling time [min]	frequency [Hz]	calc. E_{impact} [J]	calc. E_{total}[J]	temperature [°C]	yield (2)	average yield (2) ± std. dev.
					**17 Hz**						
127	15	ZrO_2	6.10	11.300	2.5	17	0.058	1443		5%	
128	15	ZrO_2	6.10	11.300	2.5	17	0.058	1443	28	6%	4 ± 1%
129	15	ZrO_2	6.10	11.300	2.5	17	0.058	1443		3%	
130	15	ZrO_2	6.10	11.300	5	17	0.058	2887		13%	
131	15	ZrO_2	6.10	11.300	5	17	0.058	2887	29	11%	10 ± 4%
132	15	ZrO_2	6.10	11.300	5	17	0.058	2887		5%	
133	15	ZrO_2	6.10	11.300	7.5	17	0.058	4330		13%	
134	15	ZrO_2	6.10	11.300	7.5	17	0.058	4330	30	19%	15 ± 3%
135	15	ZrO_2	6.10	11.300	7.5	17	0.058	4330		13%	
136	15	ZrO_2	6.10	11.300	10	17	0.058	5774		32%	
137	15	ZrO_2	6.10	11.300	10	17	0.058	5774	32	30%	27 ± 8%
138	15	ZrO_2	6.10	11.300	10	17	0.058	5774		17%	
139	15	ZrO_2	6.10	11.300	12.5	17	0.058	7217		38%	
140	15	ZrO_2	6.10	11.300	12.5	17	0.058	7217	32	32%	38 ± 6%
141	15	ZrO_2	6.10	11.300	12.5	17	0.058	7217		43%	
142	15	ZrO_2	6.10	11.300	15	17	0.058	8661		45%	
143	15	ZrO_2	6.10	11.300	15	17	0.058	8661	31	42%	45 ± 4%
144	15	ZrO_2	6.10	11.300	15	17	0.058	8661		49%	
					**15 Hz**						
145	15	ZrO_2	6.10	11.300	2.5	15	0.045	992		2%	
146	15	ZrO_2	6.10	11.300	2.5	15	0.045	992	27	0%	1 ± 1%
147	15	ZrO_2	6.10	11.300	2.5	15	0.045	992		2%	
148	15	ZrO_2	6.10	11.300	5	15	0.045	1983		4%	
149	15	ZrO_2	6.10	11.300	5	15	0.045	1983	27	7%	5 ± 1%
150	15	ZrO_2	6.10	11.300	5	15	0.045	1983		5%	
151	15	ZrO_2	6.10	11.300	7.5	15	0.045	2975		12%	
152	15	ZrO_2	6.10	11.300	7.5	15	0.045	2975	29	13%	11 ± 3%
153	15	ZrO_2	6.10	11.300	7.5	15	0.045	2975		7%	
154	15	ZrO_2	6.10	11.300	10	15	0.045	3966		19%	
155	15	ZrO_2	6.10	11.300	10	15	0.045	3966	31	16%	16 ± 3%
156	15	ZrO_2	6.10	11.300	10	15	0.045	3966		13%	
157	15	ZrO_2	6.10	11.300	12.5	15	0.045	4958		20%	
158	15	ZrO_2	6.10	11.300	12.5	15	0.045	4958	32	20%	19 ± 2%
159	15	ZrO_2	6.10	11.300	12.5	15	0.045	4958		16%	
160	15	ZrO_2	6.10	11.300	15	15	0.045	5950		28%	
161	15	ZrO_2	6.10	11.300	15	15	0.045	5950	31	30%	30 ± 2%
162	15	ZrO_2	6.10	11.300	15	15	0.045	5950		31%	

entry	ball size [mm]	ball material	material density [g/cm³]	ball mass [g]	milling time [min]	frequency [Hz]	calc. E_{impact} [J]	calc. E_{total} [J]	temperature [°C]	yield (2)	average yield (2) ± std. dev.
						**12 Hz**					
163	15	ZrO_2	6.10	11.300	2.5	12	0.029	508		0%	
164	15	ZrO_2	6.10	11.300	2.5	12	0.029	508	27	0%	0 ± 0%
165	15	ZrO_2	6.10	11.300	2.5	12	0.029	508		1%	
166	15	ZrO_2	6.10	11.300	5	12	0.029	1015		5%	
167	15	ZrO_2	6.10	11.300	5	12	0.029	1015	27	4%	3 ± 2%
168	15	ZrO_2	6.10	11.300	5	12	0.029	1015		0%	
169	15	ZrO_2	6.10	11.300	7.5	12	0.029	1523		8%	
170	15	ZrO_2	6.10	11.300	7.5	12	0.029	1523	28	9%	7 ± 3%
171	15	ZrO_2	6.10	11.300	7.5	12	0.029	1523		3%	
172	15	ZrO_2	6.10	11.300	10	12	0.029	2031		13%	
173	15	ZrO_2	6.10	11.300	10	12	0.029	2031	29	12%	10 ± 4%
174	15	ZrO_2	6.10	11.300	10	12	0.029	2031		6%	
175	15	ZrO_2	6.10	11.300	12.5	12	0.029	2538		18%	
176	15	ZrO_2	6.10	11.300	12.5	12	0.029	2538	30	15%	14 ± 5%
177	15	ZrO_2	6.10	11.300	12.5	12	0.029	2538		8%	
178	15	ZrO_2	6.10	11.300	15	12	0.029	3046		25%	
179	15	ZrO_2	6.10	11.300	15	12	0.029	3046	29	20%	21 ± 3%
180	15	ZrO_2	6.10	11.300	15	12	0.029	3046		18%	
						**10 Hz**					
181	15	ZrO_2	6.10	11.300	2.5	10	0.020	294		1%	
182	15	ZrO_2	6.10	11.300	2.5	10	0.020	294	27	1%	1 ± 0%
183	15	ZrO_2	6.10	11.300	2.5	10	0.020	294		1%	
184	15	ZrO_2	6.10	11.300	5	10	0.020	589		3%	
185	15	ZrO_2	6.10	11.300	5	10	0.020	589	27	3%	2 ± 1%
186	15	ZrO_2	6.10	11.300	5	10	0.020	589		1%	
187	15	ZrO_2	6.10	11.300	7.5	10	0.020	881		3%	
188	15	ZrO_2	6.10	11.300	7.5	10	0.020	881	28	3%	2 ± 1%
189	15	ZrO_2	6.10	11.300	7.5	10	0.020	881		1%	
190	15	ZrO_2	6.10	11.300	10	10	0.020	1175		4%	
191	15	ZrO_2	6.10	11.300	10	10	0.020	1175	29	3%	3 ± 1%
192	15	ZrO_2	6.10	11.300	10	10	0.020	1175		3%	
193	15	ZrO_2	6.10	11.300	12.5	10	0.020	1469		5%	
194	15	ZrO_2	6.10	11.300	12.5	10	0.020	1469	28	4%	5 ± 1%
195	15	ZrO_2	6.10	11.300	12.5	10	0.020	1469		5%	
196	15	ZrO_2	6.10	11.300	15	10	0.020	1763		1%	
197	15	ZrO_2	6.10	11.300	15	10	0.020	1763	26	6%	3 ± 2%
198	15	ZrO_2	6.10	11.300	15	10	0.020	1763		2%	

Table S-3. Average yields of **2** from triplicate reactions as a function of the milling time and the calculated total energies (E_{total}) for each reaction set, grouped by milling frequencies.

time [min]	average yield (2)	calculated E_{total} [J]	average yield (2)	calculated E_{total} [J]	average yield (2)	calculated E_{total} [J]
	**35 Hz**		**32 Hz**		**30 Hz**	
**2.5**	23 ± 1%	12597	18 ± 4%	9627	13 ± 1%	7933
**5**	37 ± 2%	25194	34 ± 6%	19255	40 ± 2%	15865
**7.5**	50 ± 1%	37790	61 ± 14%	28882	54 ± 5%	23798
**10**	78 ±18%	50387	85 ± 17%	38509	65 ± 5%	31731
**12.5**	92 ± 9%	62984	92 ± 10%	48137	80 ± 8%	39663
**15**	96 ± 4%	75581	95 ± 7%	57764	89 ± 5%	47596

time [min]	average yield (2)	calculated E_{total} [J]	average yield (2)	calculated E_{total} [J]	average yield (2)	calculated E_{total} [J]	average yield (2)	calculated E_{total} [J]
	**27 Hz**		**25 Hz**		**22 Hz**		**20 Hz**	
**2.5**	14 ± 2%	5783	10 ± 1%	4591	5 ± 1%	3128	6 ± 2%	2350
**5**	27 ± 3%	11566	22 ± 2%	9181	16 ± 2%	6257	19 ± 5%	4701
**7.5**	48 ± 7%	17349	40 ± 7%	13772	32 ± 3%	9385	30 ± 6%	7051
**10**	67 ± 3%	23132	51 ± 2%	18363	45 ± 1%	12514	45 ± 6%	9402
**12.5**	80 ± 1%	28915	66 ± 3%	22953	55 ± 5%	15642	53 ± 4%	11752
**15**	86 ± 2%	34698	81 ± 4%	27544	78 ± 2%	18770	65 ± 5%	14103
	**17 Hz**		**15 Hz**		**12 Hz**		**10 Hz**	
**2.5**	4 ± 1%	1443	1 ± 1%	992	0 ± 0%	508	1 ± 0%	294
**5**	10 ± 4%	2887	5 ± 1%	1983	3 ± 2%	1015	2 ± 1%	589
**7.5**	15 ± 3%	4330	11 ± 3%	2975	7 ± 3%	1523	2 ± 1%	881
**10**	27 ± 8%	5774	16 ± 3%	3966	10 ± 4%	2031	3 ± 1%	1175
**12.5**	38 ± 6%	7217	19 ± 2%	4958	14 ± 5%	2538	5 ± 1%	1469
**15**	45 ± 4%	8661	30 ± 2%	5950	21 ± 3%	3046	3 ± 2%	1763

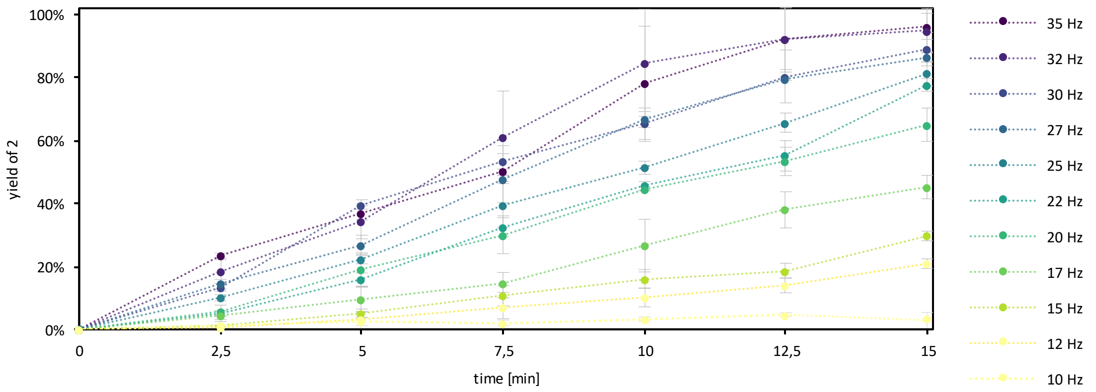

Chart S-1. Average yields of **2** from triplicate reactions as a function of milling time.

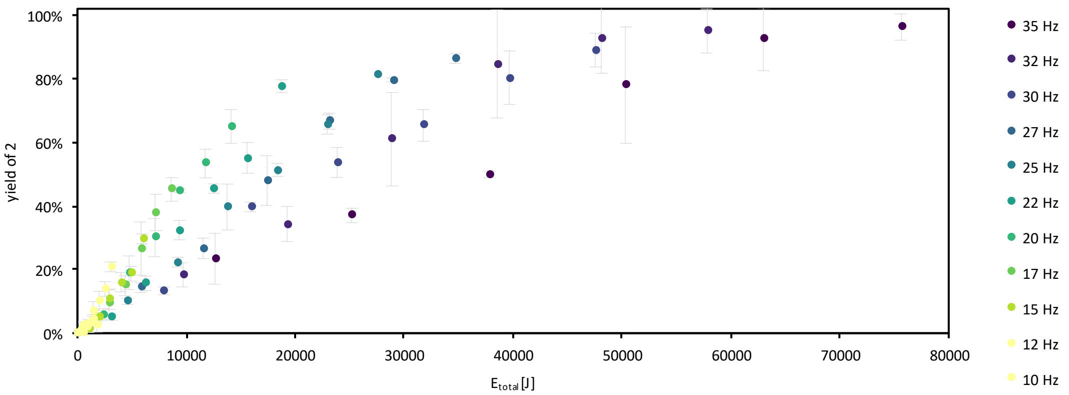

Chart S-2. Average yields of **2** from triplicate reaction as a function of milling time, plotted against the calculated total energy (E_{total}= 0-80 000)

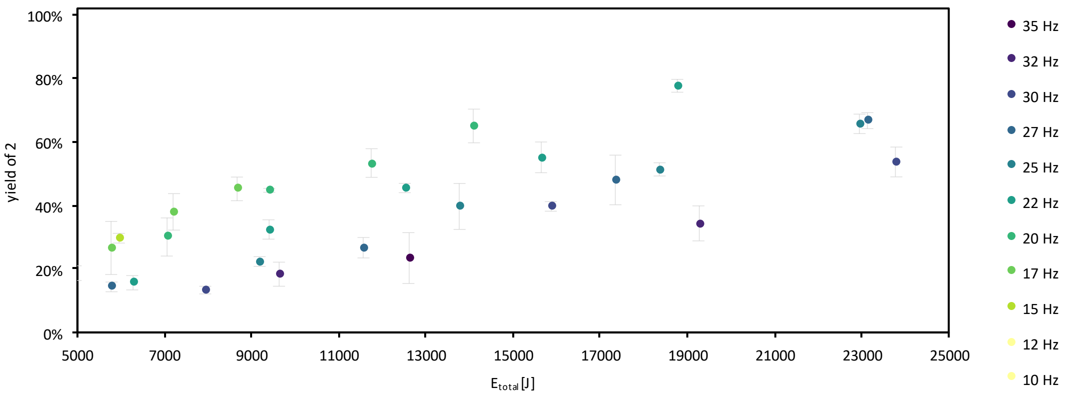

Chart S-3. Average yields of **2** from triplicate reaction as a function of milling time, plotted against the calculated total energy (E_{total}= 5 000-25 000 J)

2.4.2 Yield at a Calculated E_{total} of 12 000 J at Selected Frequencies *via* Milling Time Variation

Reactions were conducted for pre-determined milling times at selected frequency to achieve a theoretical cumulative energy (E_{total}) of 12 000 J per reaction. The calculated single impact energies (E_{impact}) and the corresponding milling times are listed in the table below. Vessel temperatures were measured immediately after milling using an infrared thermometer, and mean values from sets of 3–4 identical reactions are reported.

Although some recent reports suggest that maintaining a constant cumulative energy (E_{total}) defined as the product of the single impact energy (E_{impact}) and the number of impact events (2 × milling frequency × time), should give the product in comparable yields, our results for this specific halogen exchange reaction indicate otherwise. For reactions with the same calculated E_{total} of 12 000 J (calculated according to Lungerich[1]), observed yields differed by up to 60%, highlighting that factors beyond cumulative energy - such as frequency-dependent dynamics and aging effects - significantly influence reaction outcomes.

Table S-4. Reaction parameters and yields of **2** for reactions having a calculated E_{total} value of 12 000 J at specified milling frequencies, achieved *via* variation of milling time.

entry	ball size [mm]	ball material	material density [g/cm³]	ball mass [g]	milling time [min]	frequency [Hz]	calc. E_{impact} [J]	calc. E_{total} [J]	temperature [°C]	yield (2)	average yield (2) ± std. dev.
1	15	ZrO_2	6.10	11.054	**2.43**	35	0.241	**12000**	34	19%	19 ± 1%
2	15	ZrO_2	6.10	11.054	**2.43**	35	0.241	**12000**		20%	
3	15	ZrO_2	6.10	11.063	**2.43**	35	0.241	**12000**		19%	
4	15	ZrO_2	6.10	11.075	**2.43**	35	0.241	**12000**		19%	
5	15	ZrO_2	6.10	11.123	**3.84**	30	0.178	**12000**	35	23%	24 ± 1%
6	15	ZrO_2	6.10	11.125	**3.84**	30	0.178	**12000**		25%	
7	15	ZrO_2	6.10	11.125	**3.84**	30	0.178	**12000**		24%	
8	15	ZrO_2	6.10	11.132	**3.84**	30	0.178	**12000**		25%	
9	15	ZrO_2	6.10	11.172	**6.60**	25	0.124	**12000**	34	27%	32 ± 4%
10	15	ZrO_2	6.10	11.185	**6.60**	25	0.124	**12000**		35%	
11	15	ZrO_2	6.10	11.186	**6.60**	25	0.124	**12000**		33%	
12	15	ZrO_2	6.10	11.195	**6.60**	25	0.124	**12000**		34%	
13	15	ZrO_2	6.10	11.333	**12.72**	20	0.081	**12000**	30	44%	45 ± 3%
14	15	ZrO_2	6.10	11.337	**12.72**	20	0.081	**12000**		43%	
15	15	ZrO_2	6.10	11.340	**12.72**	20	0.081	**12000**		49%	
16	15	ZrO_2	6.10	11.348	**12.72**	20	0.081	**12000**		46%	
17	15	ZrO_2	6.10	11.361	**30.06**	15	0.045	**12000**	30	71%	70 ± 6%
18	15	ZrO_2	6.10	11.363	**30.06**	15	0.045	**12000**		65%	
19	15	ZrO_2	6.10	11.382	**30.06**	15	0.045	**12000**		78%	
20	15	ZrO_2	6.10	11.389	**30.06**	15	0.045	**12000**		66%	
21	15	ZrO_2	6.10	11.468	**100.52**	10	0.020	**12000**	28	83%	79 ± 6%
22	15	ZrO_2	6.10	11.472	**100.52**	10	0.020	**12000**		70%	
23	15	ZrO_2	6.10	11.485	**100.52**	10	0.020	**12000**		83%	
24	15	ZrO_2	6.10	11.490	**100.52**	10	0.020	**12000**		78%	

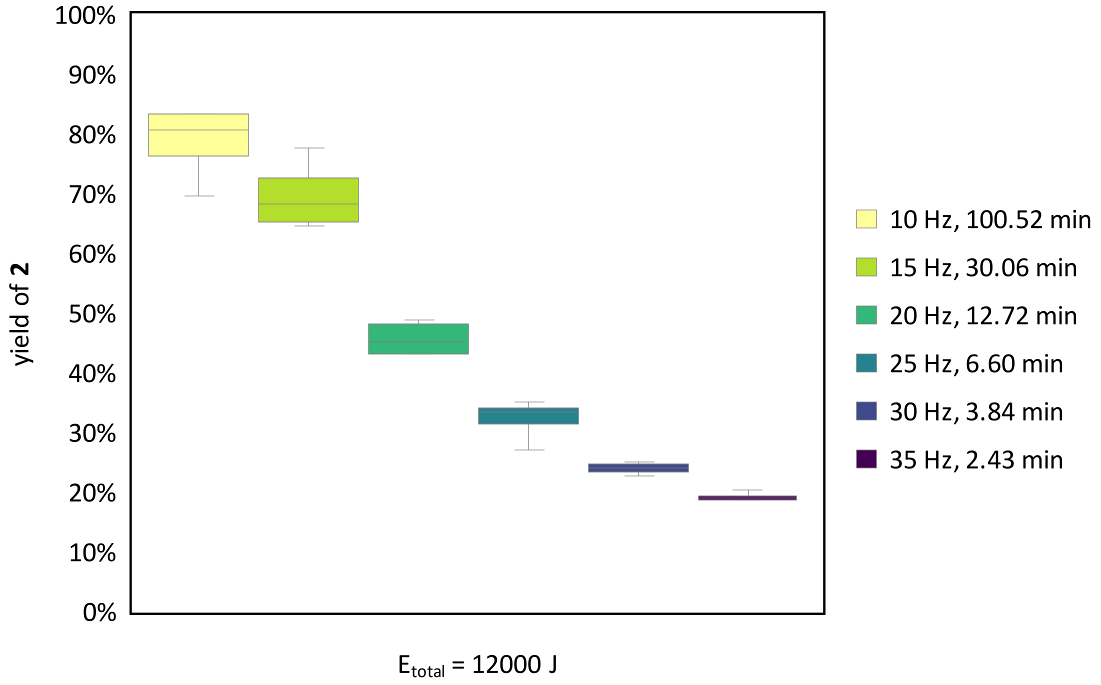

Chart S-4. Yields for specified milling frequencies for a constant, theoretical E_{total} of 12 000 J.

$$E_{total} \propto E_{impact} \cdot f \cdot t \qquad \textit{Eq. 1}$$

According to the E_{total} calculations (proportionality see Eq. 1), low-frequency reactions require much longer milling times to reach the same cumulative energy as high-frequency reactions, which achieve it in a shorter period (e.g., 10 Hz → 100.52 min vs. 35 Hz → 2.43 min). However, rather than producing comparable yields, low-frequency reactions with prolonged milling times gave significantly higher yields than their short-time, high-frequency counterparts (see **Table S-4** and **Chart S-4).** These results indicate that, for the reaction studied here, using E_{total} as a global predictor of yield is not appropriate. Consequently, we sought to identify other factors that govern reactions driven by mechanical forces.

2.4.3 Yields at a Constant Milling Time of 12.5 min and Constant Frequency of 20 Hz Using Milling Balls of Different Materials and Masses (15 mm Diameter)

These experiments were designed to investigate whether the number of net impacts (N_i) is the major driving force of the reaction. If N_i were the sole contributing factor, then conversion rates would depend only on milling ball size, frequency, and milling time, rather than on the actual weight of the milling ball. Reactions were conducted at a fixed milling time of 12.5 min and a frequency of 20 Hz. A single 15 mm milling ball was used for each reaction, while the milling ball material - and thus its weight - was varied. The balls were made of tungsten carbide (WC), chrome steel (Fe-Cr), zirconia (ZrO_2), or silicon nitride (Si_3N_4). Milling ball specifications and corresponding yields of product **2** are provided in the table below (**Table S-5**), and the results are visualized in **Chart S-5**

To determine whether temperature differences contributed to the yield variations observed in the previous experiments (20 Hz, 12.5 min *non-stop experiments*), the same reactions were repeated in consecutive milling cycles with intermittent breaks (*on/off experiments*). Here, milling was performed for 1 min followed by a 10 min pause without mechanical impact, allowing the system to cool and minimizing temperature differences. The total active milling time is listed in the table (break periods are excluded). For total active milling of 12.5 min (**Table S-6**, **entries 1–16**; 12 × 10 min breaks between cycles), yields increased significantly and plateaued around 90% for reactions using tungsten carbide,

chrome steel, or zirconia balls. Lighter silicon nitride balls gave slightly lower yields (approx. 65%), which were nevertheless higher than those obtained in the previous non-stop experiments (**Chart S-6 A**).

This overall increase in yield suggests that mechanical impact and friction can generate activated surface areas within the solid reactants, enabling reactions to continue at the interfaces even in the absence of continuous mechanical forces, presumably when the activation energy is sufficiently low to allow for ongoing reaction (aging). These observations are consistent with the results of the aging control experiments (see **Section 2.2, Control C**).

Table S-5. Reaction parameters and yields of **2** for reactions ball milled for a fixed time of 12.5 min (*non-stop*) at 20 Hz using 15 mm milling balls of different materials.

entry	ball size [mm]	ball material	material density [g/cm³]	ball mass [g]	milling time [min]	frequency [Hz]	calc. E_{impact} [J]	calc. E_{total} [J]	temperature [°C]	yield (2)	average yield (2) ± std. dev.
1	15	WC	14.90	26.226	12.50	20	0.186	27275		78%	
2	15	WC	14.90	26.234	12.50	20	0.186	27284	38	74%	76 ± 2%
3	15	WC	14.90	26.257	12.50	20	0.187	27308		77%	
4	15	Fe-Cr	7.70	13.553	12.50	20	0.096	14095		57%	
5	15	Fe-Cr	7.70	13.586	12.50	20	0.097	14130	35	58%	57 ± 2%
6	15	Fe-Cr	7.70	13.590	12.50	20	0.097	14134		55%	
7	15	ZrO_2	6.10	11.337	12.50	20	0.081	11791		51%	
8	15	ZrO_2	6.10	11.359	12.50	20	0.081	11813	35	52%	49 ± 5%
9	15	ZrO_2	6.10	11.348	12.50	20	0.081	11802		43%	
10	15	Si_3N_4	3.20	5.723	12.50	20	0.041	5952		38%	
11	15	Si_3N_4	3.20	5.741	12.50	20	0.041	5971	32	40%	40 ± 1%
12	15	Si_3N_4	3.20	5.762	12.50	20	0.041	5993		41%	

However, the plateaued yields observed in the 12.5 min on/off experiments do not allow proper kinetic analysis with respect to the influence of temperature on yield. Therefore, the reaction time in the on/off experiments was reduced to 5 min (**Table S-6, entries 17–32**; 5 × 1 min milling / 10 min break; **Chart S-6 B**), which limited temperature differences to a maximum of 5 °C. Despite this, the same trends observed in the non-stop experiments - the dependence of product formation on ball weight— were still evident.

These findings strongly suggest that the reaction is driven primarily by kinetic, rather than thermal, energy. Furthermore, results from both non-stop and on/off experiments indicate that the kinetic energy of the milling impact is the major driving factor: milling balls with higher density produce significantly higher yields than lighter ones. In contrast, if the number of net impacts were the sole driving force for starting material conversion, ball weight would be negligible, and only ball size, frequency, and milling time would matter - a hypothesis that is clearly disproven by the present results.

Table S-6. Reaction parameters and yields of **2** for reactions ball-milled at 20 Hz using 15 mm milling balls of different materials for a fixed total reaction time of 12.5 min (entries 1–16) or 5 min (entries 17–32). Milling was performed in consecutive cycles of 1 min active milling followed by a 10 min break without mechanical impact; active milling times (excluding breaks) are reported in the table.

entry	ball size [mm]	ball material	material density [g/cm³]	ball mass [g]	milling time [min]	frequency [Hz]	calc. E_{impact} [J]	calc. E_{total} [J]	temperature [°C]	yield (2)	average yield (2) ± std. dev.
1	15	WC	14.90	26.226	12.50	20	0.186	27275	28	92%	91 ± 1%
2	15	WC	14.90	26.234	12.50	20	0.186	27284		91%	
3	15	WC	14.90	26.234	12.50	20	0.186	27308		90%	
4	15	WC	14.90	26.257	12.50	20	0.187	27308		91%	
5	15	Fe-Cr	7.70	13.553	12.50	20	0.096	14095	29	87%	90 ± 2%
6	15	Fe-Cr	7.70	13.586	12.50	20	0.097	14130		92%	
7	15	Fe-Cr	7.70	13.586	12.50	20	0.097	14134		91%	
8	15	Fe-Cr	7.70	13.590	12.50	20	0.097	14144		88%	
9	15	ZrO_2	6.10	11.337	12.50	20	0.081	11786	29	85%	86 ± 1%
10	15	ZrO_2	6.10	11.359	12.50	20	0.081	11791		86%	
11	15	ZrO_2	6.10	11.359	12.50	20	0.081	11802		87%	
12	15	ZrO_2	6.10	11.348	12.50	20	0.081	11813		88%	
13	15	Si_3N_4	3.20	5.723	12.50	20	0.041	5952	28	63%	65 ± 3%
14	15	Si_3N_4	3.20	5.741	12.50	20	0.041	5971		67%	
15	15	Si_3N_4	3.20	5.741	12.50	20	0.041	5993		63%	
16	15	Si_3N_4	3.20	5.762	12.50	20	0.041	6008		68%	
17	15	WC	14.90	26.226	5.00	20	0.186	10910	31	48%	50 ± 1%
18	15	WC	14.90	26.234	5.00	20	0.186	10913		51%	
19	15	WC	14.90	26.234	5.00	20	0.186	10923		50%	
20	15	WC	14.90	26.257	5.00	20	0.187	10923		50%	
21	15	Fe-Cr	7.70	13.553	5.00	20	0.096	4906	27	27%	34 ± 6%
22	15	Fe-Cr	7.70	13.586	5.00	20	0.097	4820		32%	
23	15	Fe-Cr	7.70	13.586	5.00	20	0.097	4821		37%	
24	15	Fe-Cr	7.70	13.590	5.00	20	0.097	4826		40%	
25	15	ZrO_2	6.10	11.337	5.00	20	0.081	4715	26	28%	27 ± 2%
26	15	ZrO_2	6.10	11.359	5.00	20	0.081	4716		25%	
27	15	ZrO_2	6.10	11.359	5.00	20	0.081	4721		28%	
28	15	ZrO_2	6.10	11.348	5.00	20	0.081	4725		27%	
29	15	Si_3N_4	3.20	5.723	5.00	20	0.041	2381	27	20%	20 ± 1%
30	15	Si_3N_4	3.20	5.741	5.00	20	0.041	2388		19%	
31	15	Si_3N_4	3.20	5.741	5.00	20	0.041	2397		21%	
32	15	Si_3N_4	3.20	5.762	5.00	20	0.041	2403		20%	

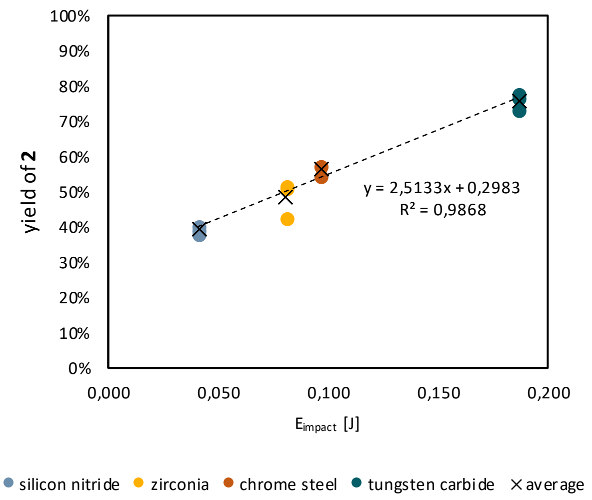

Chart S-5. Yields of **2** as a function of E_{impact} [J] for reactions performed by ball milling for a fixed time of 12.5 min (*non-stop*) at 20 Hz using 15 mm milling balls made from different materials. Black crosses indicate the average yield for each set of reactions conducted with identical ball material density, and the trendline represents the linear correlation of these averages.

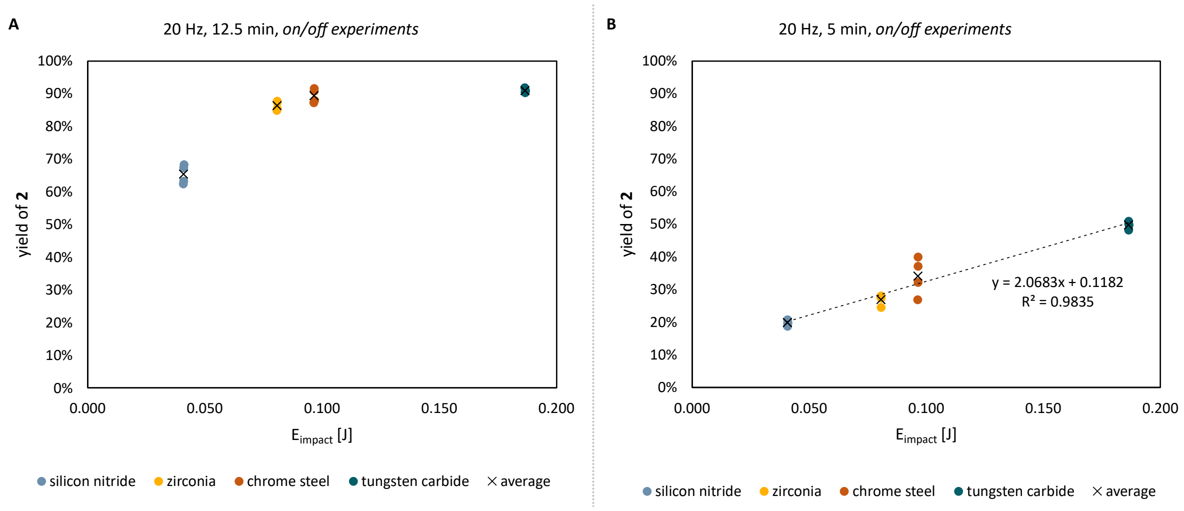

Chart S-6. Yields of **2** as a function of E_{impact} [J] for reactions ball-milled at 20 Hz using 15 mm milling balls of different materials for a fixed total reaction time of 12.5 min (**A**) or 5 min (**B**). Milling was performed in consecutive cycles of 1 min active milling followed by a 10 min break without mechanical impact. Black crosses indicate the average yield for each set of reactions conducted with identical ball material density, and the trendlines represent the linear correlation of these averages.

2.4.4 Yields at constant impact energy (E_{impact}) via ball mass and frequency variation at constant milling times

As previous experiments (see **Section 2.2, Control F** and **Section 2.4.3**) demonstrated that the kinetic energy of the impact, rather than the number of net impacts, is the predominant driving force in the halogen exchange reaction, we sought to investigate the influence of single-impact energy on the reaction outcome. To this end, several sets of experiments were carried out under constant milling time, with the milling frequency adjusted according to the weight of the milling ball in order to achieve a comparable theoretical impact energy (E_{impact}, calculated according to Lungerich[1]). Three independent sets of reactions, each targeting different E_{impact}, were conducted. All reactions were performed according to General Procedure A, while the specific milling parameters and milling material specifications are provided in the corresponding tables below.

E_{impact} = 0.079 – 0.087 J, 12.5 min:

Table S-7. Reaction parameters and yields of **2** for reactions ball milled for a fixed duration of 12.5 min, with milling frequencies adjusted according to the milling ball mass to maintain the single-impact energy (E_{impact}) within a narrow range of 0.079 – 0.087 J.

entry	ball size [mm]	ball material	material density [g/cm³]	ball mass [g]	milling time [min]	frequency [Hz]	calc. E_{impact} [J]	calc. E_{total} [J]	temperature [°C]	yield (2)	average yield (2) ± std. dev.
1	15	WC	14.90	26.226	12.50	13	**0.079**	7490	32	38%	32 ± 8%
2	15	WC	14.90	26.234	12.50	13	**0.079**	7493		28%	
3	15	WC	14.90	26.257	12.50	13	**0.079**	7499		24%	
4	15	WC	14.90	26.258	12.50	13	**0.079**	7500		39%	
5	15	Fe-Cr	7.70	13.553	12.50	19	**0.087**	12085	32	39%	46 ± 5%
6	15	Fe-Cr	7.70	13.586	12.50	19	**0.087**	12114		46%	
7	15	Fe-Cr	7.70	13.590	12.50	19	**0.087**	12118		49%	
8	15	Fe-Cr	7.70	13.600	12.50	19	**0.087**	12127		49%	
9	15	ZrO_2	6.10	11.333	12.50	20	**0.080**	11786	32	43%	51 ± 6%
10	15	ZrO_2	6.10	11.337	12.50	20	**0.081**	11790		53%	
11	15	ZrO_2	6.10	11.348	12.50	20	**0.081**	11802		56%	
12	15	ZrO_2	6.10	11.359	12.50	20	**0.081**	11813		53%	
13	15	Si_3N_4	3.20	5.723	12.50	28	**0.080**	16332	33	59%	57 ± 3%
14	15	Si_3N_4	3.20	5.741	12.50	28	**0.080**	16384		60%	
15	15	Si_3N_4	3.20	5.762	12.50	28	**0.080**	16444		54%	
16	15	Si_3N_4	3.20	5.777	12.50	28	**0.080**	16486		55%	

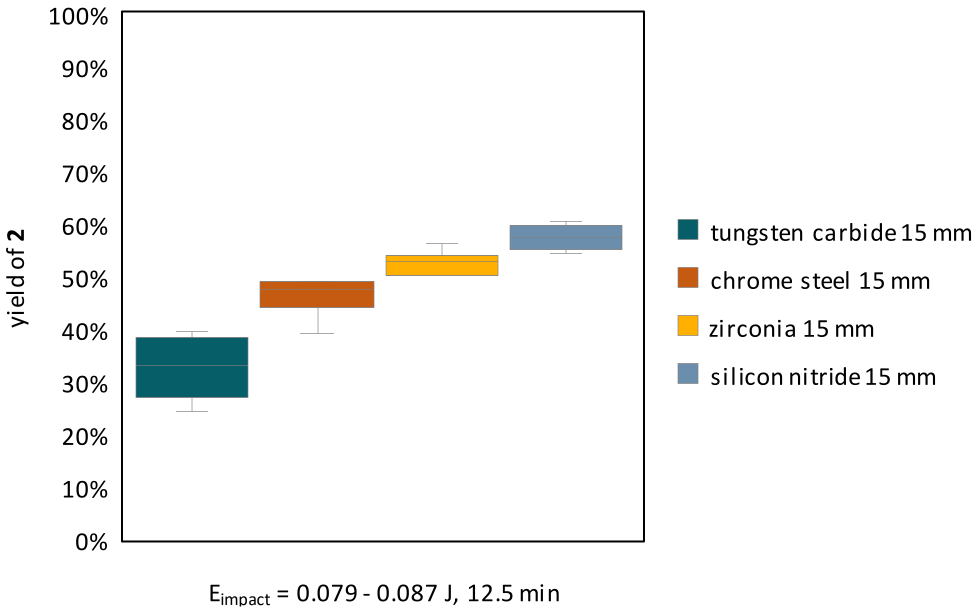

Chart S-7. Yields of **2** for reactions ball milled for a fixed duration of 12.5 min, with milling frequencies adjusted according to the milling ball mass to maintain the single-impact energy (E_{impact}) within a narrow range of 0.079 – 0.087 J.

E_{impact} = 0.089 – 0.097 J, 7.5 min:

Table S-8. Reaction parameters and yields of **2** for reactions ball milled for a fixed duration of 7.5 min, with milling frequencies adjusted according to the milling ball mass to maintain the single-impact energy (E_{impact}) within a narrow range of 0.089 – 0.097 J.

entry	ball size [mm]	ball material	material density [g/cm³]	ball mass [g]	milling time [min]	frequency [Hz]	calc. E_{impact} [J]	calc. E_{total} [J]	temperature [°C]	yield (2)	average yield (2) ± std. dev
1	15	WC	14.90	26.226	7.50	14	**0.091**	5613		31%	
2	15	WC	14.90	26.234	7.50	14	**0.091**	5615	38	38%	37 ± 5%
3	15	WC	14.90	26.257	7.50	14	**0.091**	5620		42%	
4	15	Fe-Cr	7.70	13.553	7.50	20	**0.096**	8457		43%	
5	15	Fe-Cr	7.70	13.586	7.50	20	**0.097**	8478	40	47%	46 ± 3%
6	15	Fe-Cr	7.70	13.590	7.50	20	**0.097**	8480		48%	
7	15	ZrO_2	6.10	11.333	7.50	21	**0.089**	8187		45%	
8	15	ZrO_2	6.10	11.337	7.50	21	**0.089**	8189	41	51%	50 ± 5%
9	15	ZrO_2	6.10	11.348	7.50	21	**0.089**	8197		54%	
10	15	Si_3N_4	3.20	5.723	7.50	30	**0.092**	12053		57%	
11	15	Si_3N_4	3.20	5.741	7.50	30	**0.092**	12091	45	58%	57 ± 1%
12	15	Si_3N_4	3.20	5.762	7.50	30	**0.092**	12135		57%	
13	10	WC	14.90	7.815	7.50	26	**0.094**	10977		43%	
14	10	WC	14.90	7.824	7.50	26	**0.094**	10990	45	44%	44 ± 1%
15	10	WC	14.90	7.825	7.50	26	**0.094**	10991		44%	
16	12	Fe-Cr	7.70	6.969	7.50	27	**0.090**	10945		43%	
17	12	Fe-Cr	7.70	6.973	7.50	27	**0.090**	10952	34	51%	50 ± 7%
18	12	Fe-Cr	7.70	6.979	7.50	27	**0.090**	10961		56%	

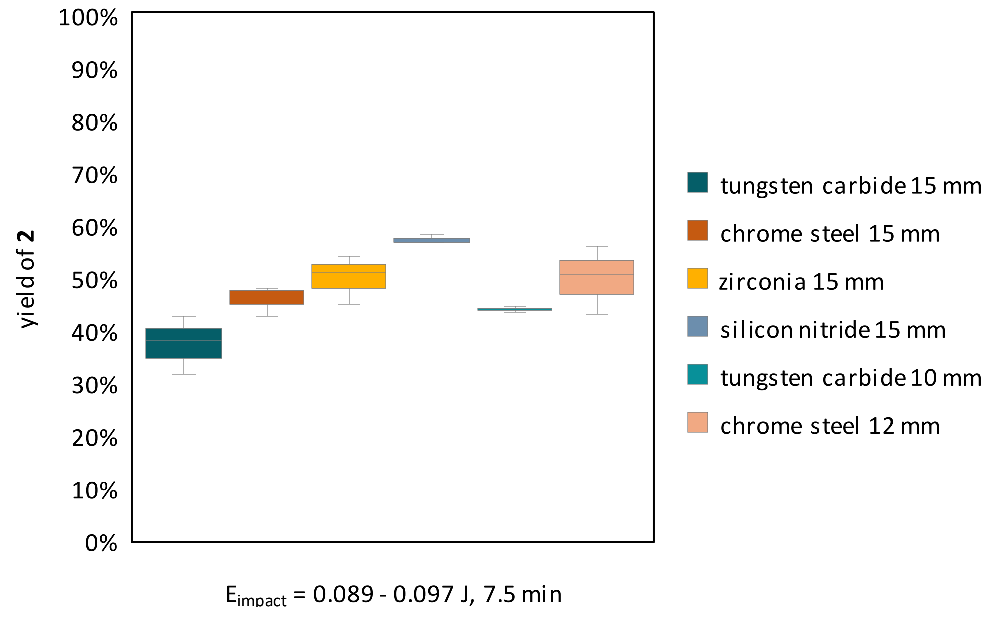

Chart S-8. Yields of **2** for reactions ball milled for a fixed duration of 7.5 min, with milling frequencies adjusted according to the milling ball mass to maintain the single-impact energy (E_{impact}) within a narrow range of 0.089 – 0.097 J.

E_{impact} = 0.116 – 0.119 J, 10 min:

Table S-9. Reaction parameters and yields of **2** for reactions ball milled for a fixed duration of 10 min, with milling frequencies adjusted according to the milling ball mass to maintain the single-impact energy (E_{impact}) within a narrow range of 0.116 – 0.119 J.

entry	ball size [mm]	ball material	material density [g/cm³]	ball mass [g]	milling time [min]	frequency [Hz]	calc. E_{impact} [J]	calc. E_{total} [J]	temperature [°C]	yield (2)	average yield (2) ± std. dev.
1	15	WC	14.90	26.226	10.00	16	**0.119**	11172		34%	
2	15	WC	14.90	26.234	10.00	16	**0.119**	11175	36	47%	40 ± 7%
3	15	WC	14.90	26.257	10.00	16	**0.119**	11185		38%	
4	15	Fe-Cr	7.70	13.553	10.00	22	**0.117**	15009		53%	
5	15	Fe-Cr	7.70	13.586	10.00	22	**0.117**	15045	38	48%	51 ± 2%
6	15	Fe-Cr	7.70	13.590	10.00	22	**0.117**	15050		51%	
7	15	ZrO_2	6.10	11.333	10.00	24	**0.116**	16294		50%	
8	15	ZrO_2	6.10	11.337	10.00	24	**0.116**	16299	36	54%	52 ± 2%
9	15	ZrO_2	6.10	11.348	10.00	24	**0.116**	16315		52%	
10	15	Si_3N_4	3.20	5.723	10.00	34	**0.118**	23394		65%	
11	15	Si_3N_4	3.20	5.741	10.00	34	**0.118**	23467	39	59%	62 ± 3%
12	15	Si_3N_4	3.20	5.762	10.00	34	**0.118**	23553		63%	
13	10	WC	14.90	7.815	10.00	29	**0.117**	20310		59%	
14	10	WC	14.90	7.824	10.00	29	**0.117**	20333	45	57%	57 ± 2%
15	10	WC	14.90	7.825	10.00	29	**0.117**	20336		56%	
16	12	Fe-Cr	7.70	6.969	10.00	31	**0.119**	22089		49%	
17	12	Fe-Cr	7.70	6.973	10.00	31	**0.119**	22101	36	45%	49 ± 4%
18	12	Fe-Cr	7.70	6.979	10.00	31	**0.119**	22120		54%	

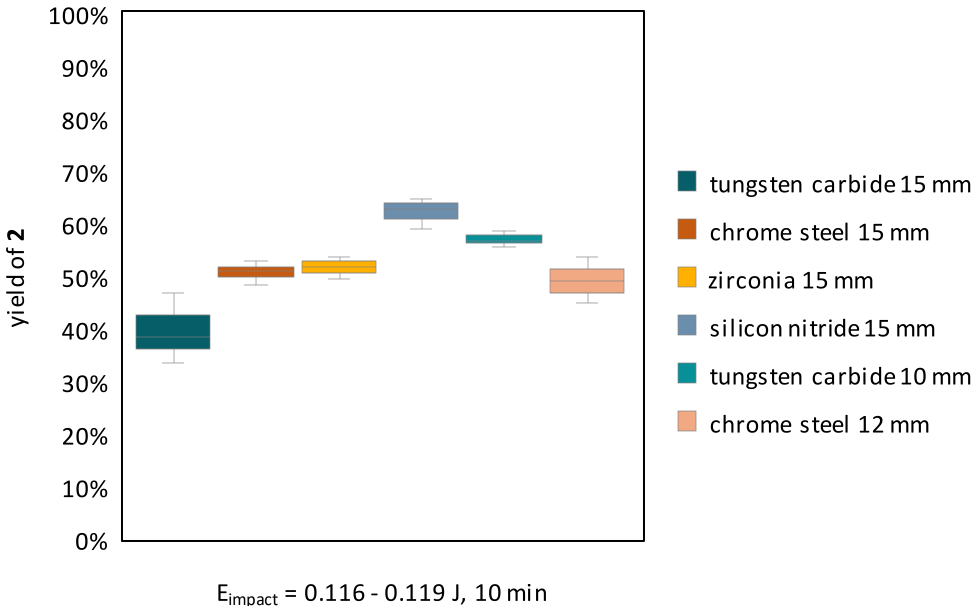

Chart S-9. Yields of **2** for reactions ball milled for a fixed duration of 10 min, with milling frequencies adjusted according to the milling ball mass to maintain the single-impact energy (E_{impact}) within a narrow range of 0.116 – 0.119 J.

2.4.5 Yields at Comparable E_{impact} via Frequency Adjustment for Ball Milling Masses with Total Energy (E_{total}) Equalized by Adjusting Milling Times to Standardize the Number of Theoretical Impacts

In the previous set of reactions (see **Section 2.4.4**), where the E_{impact} values were constrained within a narrow range and reactions were performed for a constant milling time, the observed differences in yields could potentially be attributed to variations in the number of theoretical impacts at different frequencies (assuming the milling ball diameter is approximately equal to the inner diameter of the vessel). For instance, in a reaction performed at 16 Hz, the ball would theoretically strike the vessel walls twice per oscillation, resulting in 32 impacts per second. In contrast, at 34 Hz, the ball would make approximately 68 impacts per second - roughly twice as many. By standardizing the number of theoretical impacts across different frequencies, the cumulative energy delivered to the system (E_{total}) can also be equalized. To achieve this, the reaction times of the target experiments were adjusted ($t_{adjusted}$) relative to a reference experiment conducted at a reference frequency ($f_{reference}$) and a reference milling time ($t_{reference}$) using a frequency ratio (FR) as follows:

$$FR = \frac{f_{reference}}{f_{target}} \qquad \textbf{Eq. 2}$$

$$t_{adjusted} = t_{reference} \times FR \qquad \textbf{Eq. 3}$$

In the following, the reference experiment is indicated in the respective table and highlighted in green in the corresponding chart. The times listed in the tables correspond to the adjusted times ($t_{adjusted}$) calculated using the equations above. All reactions were performed according to General Procedure A. Reaction and milling parameters are provided in the tables below.

It should be noted that at 14 Hz, when using a relatively heavy tungsten carbide milling ball, the ball's movement is sluggish and rather inert at such low frequencies, likely resulting in a significantly reduced number of effective impact events.

E_{impact} = 0.089 – 0.097 J, reference experiment at 30 Hz for 7.5 min

Table S-10. Reaction parameters and yields of **2** for reactions with comparable E_{impact} and E_{total}. Reference experiments are indicated in entries 10–12 (green); for all other entries, the milling times were adjusted to equalize the number of impacts relative to the reference experiments.

entry	ball size [mm]	ball material	material density [g/cm³]	ball mass [g]	milling time [min]	frequency [Hz]	calc. E_{impact} [J]	calc. E_{total} [J]	temperature [°C]	yield (2)	average yield (2) ± std. dev.
1	15	WC	14.90	26.226	**16.07**	14	0.091	12005		66%	
2	15	WC	14.90	26.234	**16.07**	14	0.091	12009	33	54%	58 ± 7%
3	15	WC	14.90	26.257	**16.07**	14	0.091	12019		55%	
4	15	Fe-Cr	7.70	13.553	**11.25**	20	0.096	12686		53%	
5	15	Fe-Cr	7.70	13.586	**11.25**	20	0.097	12717	34	56%	55 ± 2%
6	15	Fe-Cr	7.70	13.590	**11.25**	20	0.097	12720		57%	
7	15	ZrO_2	6.10	11.333	**10.71**	21	0.089	11690		53%	
8	15	ZrO_2	6.10	11.337	**10.71**	21	0.089	11695	32	51%	51 ± 2%
9	15	ZrO_2	6.10	11.348	**10.72**	21	0.089	11706		49%	
10	15	Si_3N_4	3.20	5.723	7.50	30	0.092	12053		57%	
11	15	Si_3N_4	3.20	5.741	7.50	30	0.092	12091	45	58%	57 ± 1%
12	15	Si_3N_4	3.20	5.762	7.50	30	0.092	12135		57%	
13	10	WC	14.90	7.815	**8.65**	26	0.094	12600		42%	
14	10	WC	14.90	7.824	**8.65**	26	0.094	12675	34	47%	46 ± 3%
15	10	WC	14.90	7.825	**8.65**	26	0.094	12676		48%	
16	12	Fe-Cr	7.70	6.969	**8.33**	27	0.090	12157		44%	
17	12	Fe-Cr	7.70	6.973	**8.33**	27	0.090	12164	37	49%	48 ± 4%
18	12	Fe-Cr	7.70	6.979	**8.33**	27	0.090	12174		51%	

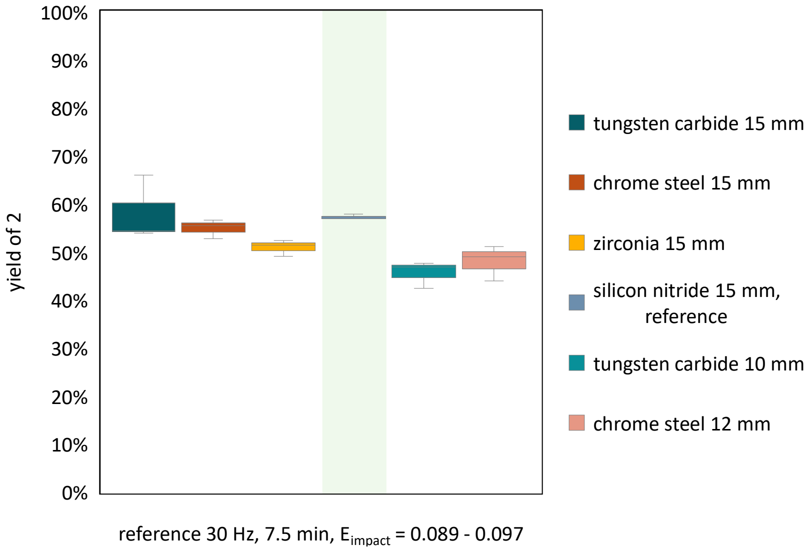

Chart S-10. Yields of **2** for reactions with comparable E_{impact} and E_{total}. Reference experiments are shown in the fourth box from the left (green); for all other entries, the milling times were adjusted to equalize the number of impacts relative to the reference experiments.

E_{impact} = 0.089 – 0.097 J, reference experiment at 21 Hz for 7.5 min

Table S-11. Reaction parameters and yields of **2** for reactions with comparable E_{impact} and E_{total}. Reference experiments are indicated in entries 7-9 (green); for all other entries, the milling times were adjusted to equalize the number of impacts relative to the reference experiments.

entry	ball size [mm]	ball material	material density [g/cm³]	ball mass [g]	milling time [min]	frequency [Hz]	calc. E_{impact} [J]	calc. E_{total} [J]	temperature [°C]	yield (2)	average yield (2) ± std.dev.
1	15	WC	14.90	26.226	**11.25**	14	0.091	8420		42%	
2	15	WC	14.90	26.234	**11.25**	14	0.091	8422	31	46%	41 ± 5%
3	15	WC	14.90	26.257	**11.25**	14	0.091	8430		36%	
4	15	Fe-Cr	7.70	13.553	**7.88**	20	0.096	8886		39%	
5	15	Fe-Cr	7.70	13.586	**7.88**	20	0.097	8907	31	40%	40 ± 2%
6	15	Fe-Cr	7.70	13.590	**7.88**	20	0.097	8910		42%	
7	15	ZrO_2	6.10	11.333	7.50	21	0.089	8187		45%	
8	15	ZrO_2	6.10	11.337	7.50	21	0.089	8189	36	51%	50 ± 5%
9	15	ZrO_2	6.10	11.348	7.50	21	0.089	8197		54%	
10	15	Si_3N_4	3.20	5.723	**5.25**	30	0.092	8437		30%	
11	15	Si_3N_4	3.20	5.741	**5.25**	30	0.092	8463	33	28%	30 ± 3%
12	15	Si_3N_4	3.20	5.762	**5.25**	30	0.092	8494		33%	
13	10	WC	14.90	7.815	**6.06**	26	0.094	8870		23%	
14	10	WC	14.90	7.824	**6.06**	26	0.094	8880	35	32%	29 ± 6%
15	10	WC	14.90	7.825	**6.06**	26	0.094	8881		33%	
16	12	Fe-Cr	7.70	6.969	**5.83**	27	0.090	8508		25%	
17	12	Fe-Cr	7.70	6.973	**5.83**	27	0.090	8513	33	24%	28 ± 6%
18	12	Fe-Cr	7.70	6.979	**5.83**	27	0.090	8520		34%	

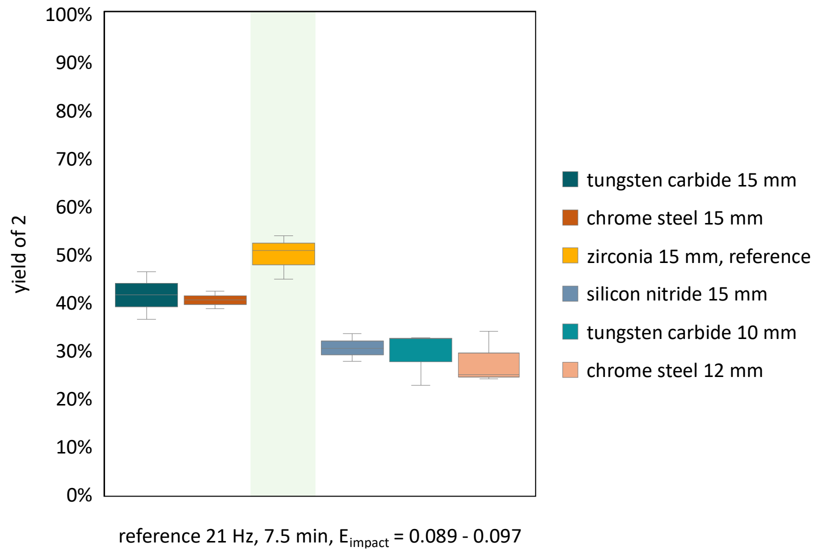

Chart S-11. Yields of **2** for reactions with comparable E_{impact} and E_{total}. Reference experiments are shown in the third box from the left (green); for all other entries, the milling times were adjusted to equalize the number of impacts relative to the reference experiments.

E_{impact} = 0.116 – 0.119 J, reference experiment at 34 Hz for 10 min

Table S-12. Reaction parameters and yields of **2** for reactions with comparable E_{impact} and E_{total}. Reference experiments are indicated in entries 10–12 (green); for all other entries, the milling times were adjusted to equalize the number of impacts relative to the reference experiments.

entry	ball size [mm]	ball material	material density [g/cm³]	ball mass [g]	milling time [min]	frequency [Hz]	calc. E_{impact} [J]	calc. E_{total} [J]	temperature [°C]	yield (2)	average yield (2) ± std.dev.
1	15	WC	14.90	26.226	**21.25**	16	0.119	23740		93%	
2	15	WC	14.90	26.234	**21.25**	16	0.119	23748	45	91%	90 ± 3%
3	15	WC	14.90	26.257	**21.25**	16	0.119	23768		86%	
4	15	Fe-Cr	7.70	13.553	**15.45**	22	0.117	23188		83%	
5	15	Fe-Cr	7.70	13.586	**15.45**	22	0.117	23245	41	76%	79 ± 3%
6	15	Fe-Cr	7.70	13.590	**15.45**	22	0.117	23252		77%	
7	15	ZrO_2	6.10	11.333	**14.17**	24	0.116	23088		86%	
8	15	ZrO_2	6.10	11.337	**14.17**	24	0.116	23096	47	77%	81 ± 4%
9	15	ZrO_2	6.10	11.348	**14.17**	24	0.116	23119		81%	
10	15	Si_3N_4	3.20	5.723	10.00	34	0.118	23394		65%	
11	15	Si_3N_4	3.20	5.741	10.00	34	0.118	23467	39	59%	62 ± 3%
12	15	Si_3N_4	3.20	5.762	10.00	34	0.118	23553		63%	
13	10	WC	14.90	7.815	**11.72**	29	0.117	23803		50%	
14	10	WC	14.90	7.824	**11.72**	29	0.117	23830	40	49%	49 ± 0%
15	10	WC	14.90	7.825	**11.72**	29	0.117	23833		49%	
16	12	Fe-Cr	7.70	6.969	**10.97**	31	0.119	24231		68%	
17	12	Fe-Cr	7.70	6.973	**10.97**	31	0.119	24245	45	72%	68 ± 3%
18	12	Fe-Cr	7.70	6.979	**10.97**	31	0.119	24266		65%	

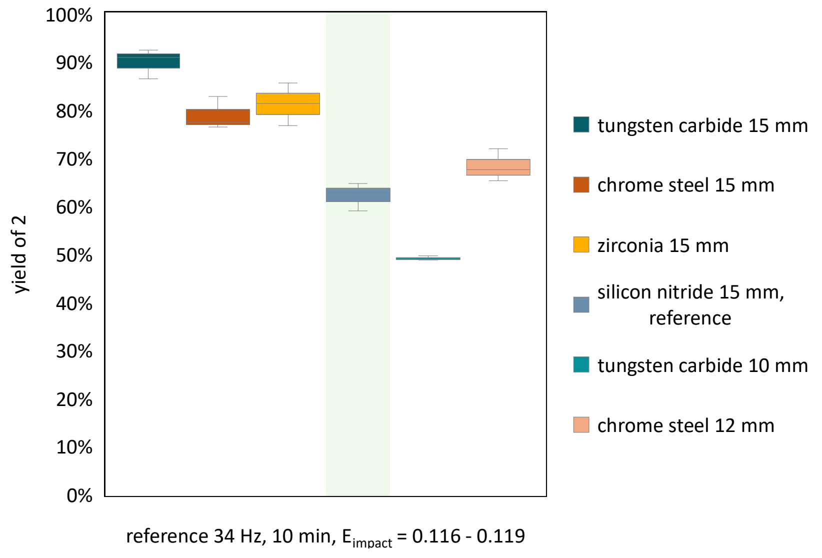

Chart S-12. Yields of **2** for reactions with comparable E_{impact} and E_{total}. Reference experiments are shown in the fourth box from the left (green); for all other entries, the milling times were adjusted to equalize the number of impacts relative to the reference experiments

These results suggest that, for the halogen exchange reaction, higher single-impact energies (E_{impact}) can more effectively activate the reactive mixture, generating fresh contact surfaces between the reactants. This increased reactive interface may facilitate higher conversions even after the initial impact. Consequently, the phenomenon we refer to as aging - the continuation of the reaction after the direct mechanical impacts - is likely more pronounced at higher E_{impact}, where more reactive contact area is created, than at lower E_{impact}. This trend was also observed in the initial control experiments for aging (see **Section 2.2, Control C**), where reactions conducted at lower frequencies (20 Hz, lower E_{impact}) showed smaller yield increases, whereas higher frequencies (30 Hz, higher E_{impact}) led to larger increases attributable to aging.

When comparing the experiments within this section, conducted exclusively under solid-reactant conditions (no LAG or liquid by-products), the assumption that higher E_{impact} generates more reactive contact area - and thus more extensive aging - is supported. This is evident when comparing results at E_{impact}=0.089–0.097 J (**Table S-10, Chart S-10; Table S-11, Chart S-11**) with those at E_{impact}=0.116–0.119 J (**Table S-12, Chart S-12**).

When normalizing the number of impacts and E_{total} by adjusting reaction times relative to a reference experiment via the frequency ratio (see Eq. 3), reactions at lower E_{impact} showed reasonably consistent average yields, although perfect equalization was not achieved (cf. **Table S-8, Chart S-8** vs. **Tables S-10** and **S-11**, **Charts S-10** and **S-11**). In contrast, for reactions at higher E_{impact}, adjusting reaction times failed to reach comparable yields, and the yields of the adjusted reactions significantly exceeded those of the reference experiment (cf. **Table S-9, Chart S-9** vs. **Table S-12, Chart S-12**). This is likely due to the greater generation of reactive contact surfaces at higher E_{impact}. With prolonged reaction time ($t_{adjusted}$), the reaction has more time to proceed *via* aging, i.e., to continue reacting independently of direct mechanical impact. For higher E_{impact}, this effect becomes more pronounced, as more reactive contact area allows aging to occur over a larger portion of the mixture, causing yields to "overshoot" relative to the reference experiment.

We hypothesize that the extent of aging depends on the inherent properties of the reaction itself. Some reactions, such as the halogen exchange studied here, continue to proceed after the impact events, whereas others, such as the Wittig olefination (**Section 4**), do not. Pre-activation of a single component alone was found not to enhance the halogen exchange reaction (pre-milling NaI or 4-(Bromomethyl)-1,1'-biphenyl **(1)** separately; see **Section 2.2, Control H**), indicating that factors other than individual surface activation govern aging. Further studies are needed to determine the specific conditions or factors that enable aging in some reactions but not in others. Based on these results, we propose that total energy (E_{total}) may not be an appropriate parameter for interconverting or comparing milling conditions in pure all-solid reactions that exhibit aging. Instead, single-impact energy combined with overall reaction time appears to play a more significant role, particularly in reactions with low activation energy where aging can occur.

2.4.6 Effects of Different Liquid-Assisted Grinding (LAG) Agents on the Yields at a Constant Milling Time of 12.5 min and Constant Frequency of 20 Hz Using Milling Balls of Different Materials and Masses (15 mm Diameter Each)

The following experiments were designed to elucidate the role of LAG agents in the halogen exchange reaction. For this purpose, we selected solvents with distinct solvation capacities for the two reaction components. Water and chloroform—both poor solvents for the halogen exchange reaction as a whole—were chosen to selectively solubilize a single component: sodium iodide in the case of water and the organic starting material in the case of chloroform. By enhancing the solubility of only one component, we aimed to test whether the reaction is still primarily driven by kinetic impact or if the increased mobility of one reagent shifts the driving force toward a diffusion-dominated regime. In the latter case, conducting the reaction under LAG conditions would be expected to result in yields determined solely by the milling parameters (ball size, milling time, and frequency), but independent of milling ball weight (cf. **Section 2.4.3**).

The reactions were carried out according to General Procedure A, with the deviation that the respective LAG agent was added via microsyringe to the solid reactants prior to milling. Deuterated solvents were employed to minimize solvent peaks in the subsequent NMR analysis. For each reaction, 30 µL of solvent was added, corresponding to an η-value of 0.2 (expressed in µL per mg solid reactants).

<u>No LAG, 20 Hz, 12.5 min:</u>

Table S-13. Reaction parameters and yields of **2** for reactions ball-milled for 12.5 min at a constant frequency of 20 Hz using 15 mm milling balls of different materials, without the addition of a LAG agent.

entry	ball size [mm]	ball material	material density [g/cm³]	ball mass [g]	milling time [min]	frequency [Hz]	calc. E_{impact} [J]	calc. E_{total} [J]	temperature [°C]	yield (2)	average yield (2) ± std. dev.
1	15	WC	14.90	26.226	12.50	20	0.186	27275		78%	
2	15	WC	14.90	26.234	12.50	20	0.186	27284	38	74%	76 ± 2%
3	15	WC	14.90	26.257	12.50	20	0.187	27308		77%	
4	15	Fe-Cr	7.70	13.553	12.50	20	0.096	14095		57%	
5	15	Fe-Cr	7.70	13.586	12.50	20	0.097	14130	35	58%	57 ± 2%
6	15	Fe-Cr	7.70	13.590	12.50	20	0.097	14134		55%	
7	15	ZrO_2	6.10	11.337	12.50	20	0.081	11791		51%	
8	15	ZrO_2	6.10	11.359	12.50	20	0.081	11813	35	52%	49 ± 5%
9	15	ZrO_2	6.10	11.348	12.50	20	0.081	11802		43%	
10	15	Si_3N_4	3.20	5.723	12.50	20	0.041	5952		38%	
11	15	Si_3N_4	3.20	5.741	12.50	20	0.041	5971	32	40%	40 ± 1%
12	15	Si_3N_4	3.20	5.762	12.50	20	0.041	5993		41%	

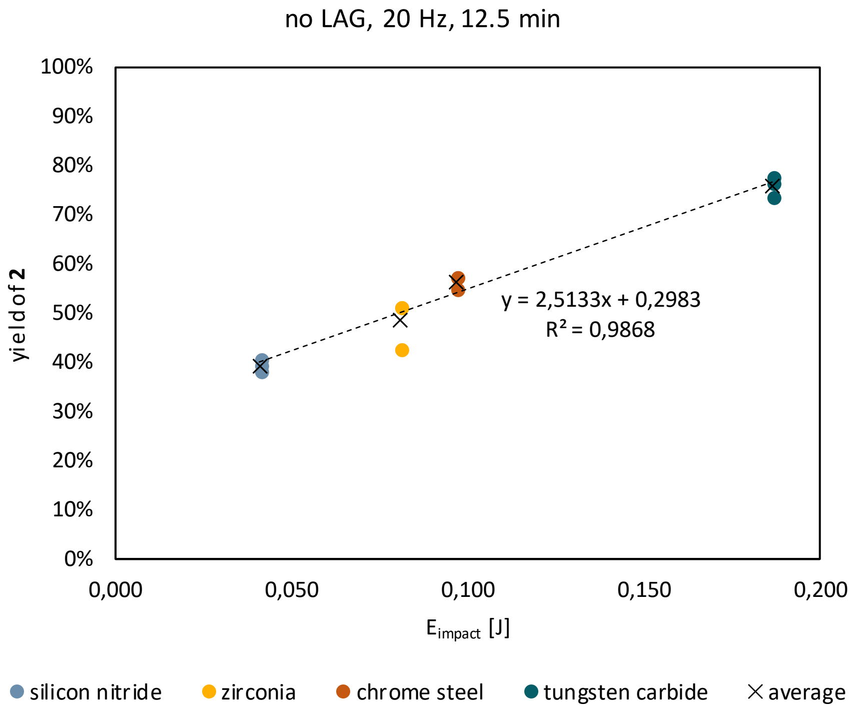

Chart S-13. Yields of **2** as a function of E_{impact} [J] for reactions performed by ball milling for a fixed time of 12.5 min at 20 Hz using 15 mm milling balls made from different materials, without the addition of a LAG agent. Black crosses indicate the average yield for each set of reactions conducted with identical E_{impact}, and the trendline represents the linear correlation of these averages.

D_2O as LAG, 20 Hz, 12.5 min:

Table S-14. Reaction parameters and yields of **2** for reactions ball-milled for 12.5 min at a constant frequency of 20 Hz using 15 mm milling balls of different materials, with 30 µL D_2O as LAG.

entry	ball size [mm]	ball material	material density [g/cm³]	ball mass [g]	milling time [min]	frequency [Hz]	calc. E_{impact} [J]	calc. E_{total} [J]	temperature [°C]	yield (2)	average yield (2) ± std. dev.
1	15	WC	14.90	26.226	12.50	20	0.186	27275		34%	
2	15	WC	14.90	26.234	12.50	20	0.186	27284	38	42%	41 ± 7%
3	15	WC	14.90	26.257	12.50	20	0.187	27308		47%	
4	15	Fe-Cr	7.70	13.553	12.50	20	0.096	14095		23%	
5	15	Fe-Cr	7.70	13.586	12.50	20	0.097	14130	33	31%	26 ± 5%
6	15	Fe-Cr	7.70	13.590	12.50	20	0.097	14134		24%	
7	15	ZrO_2	6.10	11.337	12.50	20	0.081	11791		19%	
8	15	ZrO_2	6.10	11.359	12.50	20	0.081	11813	30	11%	18 ± 7%
9	15	ZrO_2	6.10	11.348	12.50	20	0.081	11802		24%	
10	15	Si_3N_4	3.20	5.723	12.50	20	0.041	5952		16%	
11	15	Si_3N_4	3.20	5.741	12.50	20	0.041	5971	28	15%	14 ± 3%
12	15	Si_3N_4	3.20	5.762	12.50	20	0.041	5993		11%	

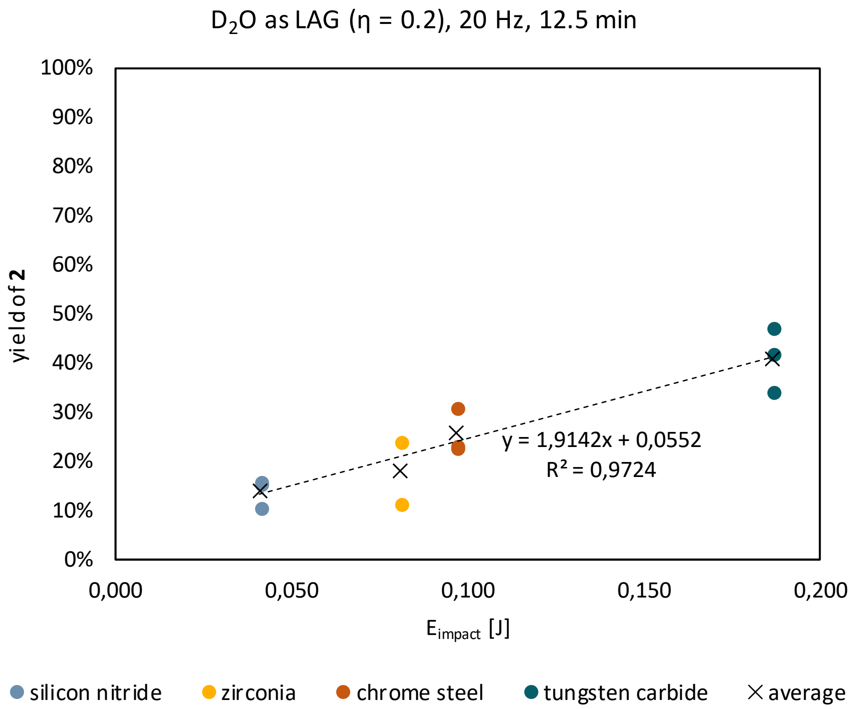

Chart S-14. Yields of **2** as a function of E_{impact} [J] for reactions performed by ball milling for a fixed time of 12.5 min at 20 Hz using 15 mm milling balls made from different materials, with 30 µL D_2O as LAG. Black crosses indicate the average yield for each set of reactions conducted with identical E_{impact}, and the trendline represents the linear correlation of these averages.

$CDCl_3$ as LAG, 20 Hz, 12.5 min:

Table S-15. Reaction parameters and yields of **2** for reactions ball-milled for 12.5 min at a constant frequency of 20 Hz using 15 mm milling balls of different materials, with 30 µL $CDCl_3$ as LAG.

entry	ball size [mm]	ball material	material density [g/cm³]	ball mass [g]	milling time [min]	frequency [Hz]	calc. E_{impact} [J]	calc. E_{total} [J]	temperature [°C]	yield (2)	average yield (2) ± std. dev.
1	15	WC	14.90	26.226	12.50	20	0.186	27275		22%	
2	15	WC	14.90	26.234	12.50	20	0.186	27284	33	33%	27 ± 6%
3	15	WC	14.90	26.257	12.50	20	0.187	27308		25%	
4	15	Fe-Cr	7.70	13.553	12.50	20	0.096	14095		28%	
5	15	Fe-Cr	7.70	13.586	12.50	20	0.097	14130	34	20%	23 ± 5%
6	15	Fe-Cr	7.70	13.590	12.50	20	0.097	14134		20%	
7	15	ZrO_2	6.10	11.337	12.50	20	0.081	11791		14%	
8	15	ZrO_2	6.10	11.359	12.50	20	0.081	11813	34	17%	16 ± 2%
9	15	ZrO_2	6.10	11.348	12.50	20	0.081	11802		18%	
10	15	Si_3N_4	3.20	5.723	12.50	20	0.041	5952		7%	
11	15	Si_3N_4	3.20	5.741	12.50	20	0.041	5971	28	7%	7 ± 0%
12	15	Si_3N_4	3.20	5.762	12.50	20	0.041	5993		6%	

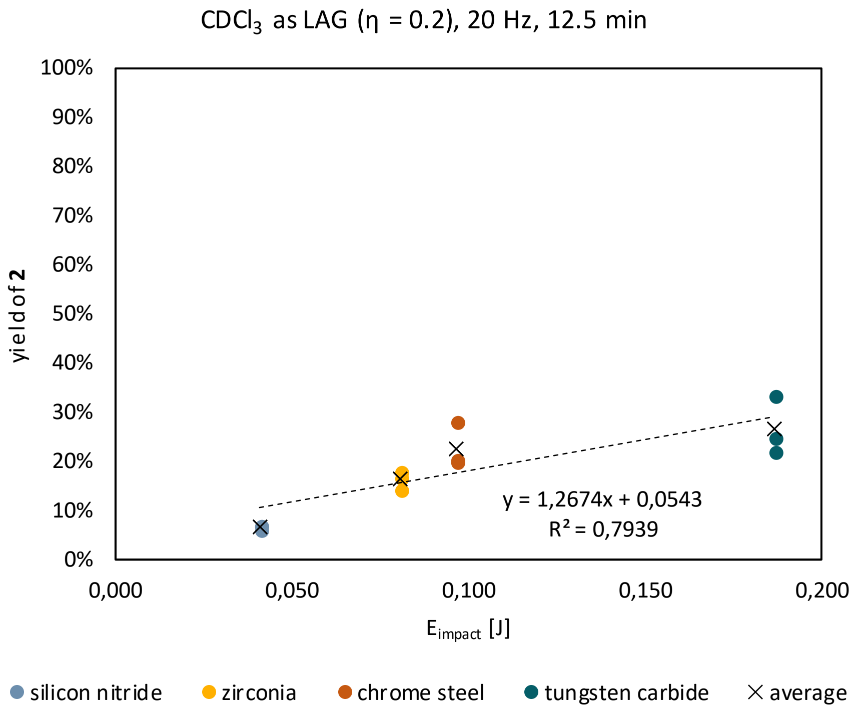

Chart S-15. Yields of **2** as a function of E_{impact} [J] for reactions performed by ball milling for a fixed time of 12.5 min at 20 Hz using 15 mm milling balls made from different materials, with 30 µL $CDCl_3$ as LAG. Black crosses indicate the average yield for each set of reactions conducted with identical E_{impact}, and the trendline represents the linear correlation of these averages.

Acetone-d_6 as LAG, 20 Hz, 12.5 min:

Table S-16. Reaction parameters and yields of **2** for reactions ball-milled for 12.5 min at a constant frequency of 20 Hz using 15 mm milling balls of different materials, with 30 µL acetone-d_6 as LAG.

entry	ball size [mm]	ball material	material density [g/cm³]	ball mass [g]	milling time [min]	frequency [Hz]	calc. E_{impact} [J]	calc. E_{total} [J]	temperature [°C]	yield (2)	average yield (2) ± std.dev.
1	15	WC	14.90	26.226	12.50	20	0.186	27275		99%	
2	15	WC	14.90	26.234	12.50	20	0.186	27284	38	94%	97 ± 2%
3	15	WC	14.90	26.257	12.50	20	0.187	27308		96%	
4	15	Fe-Cr	7.70	13.553	12.50	20	0.096	14095		96%	
5	15	Fe-Cr	7.70	13.586	12.50	20	0.097	14130	32	91%	95 ± 4%
6	15	Fe-Cr	7.70	13.590	12.50	20	0.097	14134		98%	
7	15	ZrO_2	6.10	11.337	12.50	20	0.081	11791		99%	
8	15	ZrO_2	6.10	11.359	12.50	20	0.081	11813	30	97%	98 ± 1%
9	15	ZrO_2	6.10	11.348	12.50	20	0.081	11802		99%	
10	15	Si_3N_4	3.20	5.723	12.50	20	0.041	5952		99%	
11	15	Si_3N_4	3.20	5.741	12.50	20	0.041	5971	30	97%	98 ± 1%
12	15	Si_3N_4	3.20	5.762	12.50	20	0.041	5993		99%	

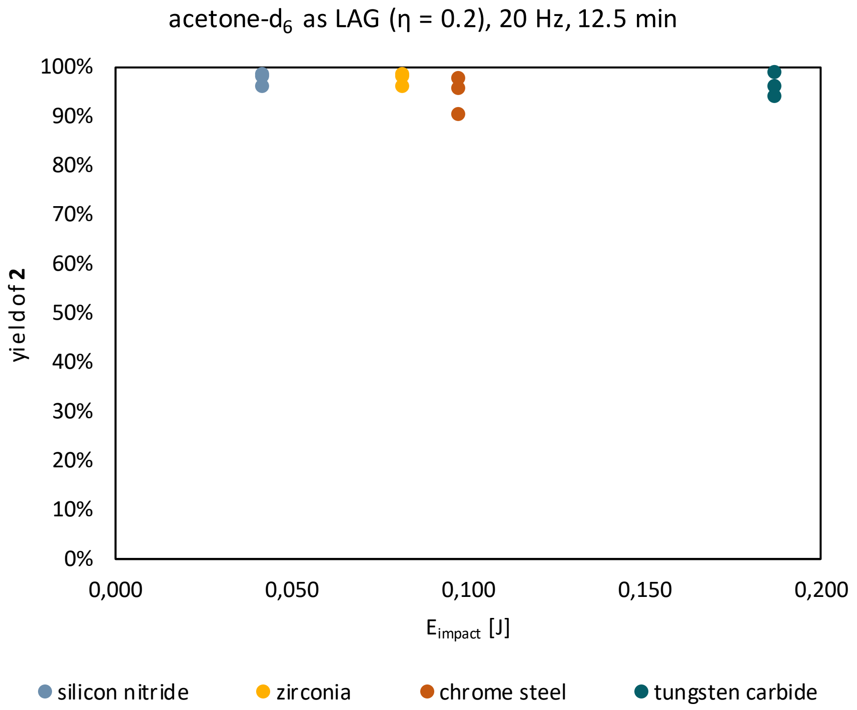

Chart S-16. Yields of **2** as a function of E_{impact} [J] for reactions performed by ball milling for a fixed time of 12.5 min at 20 Hz using 15 mm milling balls made from different materials, with 30 µL acetone-d_6 as LAG. Black crosses indicate the average yield for each set of reactions conducted with identical E_{impact}, and the trendline represents the linear correlation of these averages.

Typically, the Finkelstein halogen-exchange reaction is conducted in acetone, a solvent capable of dissolving NaI, the organic starting material, and the product, but not NaBr. The driving force of the reaction under such conditions is the precipitation of NaBr, which shifts the equilibrium toward the product side. As expected, the addition of 30 µL acetone as LAG drastically accelerates the reaction, leading to full conversion in reaction times shorter than 12.5 min. Moreover, it was confirmed that in the presence of acetone as LAG, the reaction proceeds rapidly even without milling. Thus, acetone is not suitable for studying mechanochemical kinetics, as it alters the process and makes solution-based reactivities more pronounced.

In contrast, using solvents that do not favor the halogen exchange, such as water (dissolving both NaI and NaBr) or chloroform (dissolving neither NaI nor NaBr), as LAG agents results in an overall decrease in conversion of the starting material. Interestingly, however, the same trends observed for no-LAG reactions persist: higher single-impact energies (E_{impact}) result in higher product yields than lower E_{impact} (cf. **Table S-13** vs. **Table S-14** and **Table S-15**, **entries 1-3** for tungsten carbide and **entries 10-12** for silicon nitride). These findings strongly suggest that while LAG additives can modulate the overall reaction rate and conversion, they do not alter the fundamental impact-energy-driven nature of mechanochemical halogen exchange.

Additional evidence supporting this conclusion is provided in **Section 4**, where the Wittig olefination was investigated under LAG conditions.

3. The $KMnO_4$ oxidation

The oxidation of a solid substrate containing a secondary alcohol with potassium permanganate, carried out without the addition of external LAG agents, was tested as an alternative all-solid reaction.[2] In this case, stoichiometric amounts of water are formed as a byproduct, effectively acting as an *in situ* generated LAG additive. However, this reaction was not fully suitable for validating all previous observations, as the progressively formed manganese dioxide (MnO_2) moderated the oxidation and reduced the reaction rate, complicating kinetic analysis (details see **Section 3.4.4** below). Nevertheless, the system proved valuable in confirming that the primary driving force is the kinetic energy imparted by mechanical impact, rather than particle translocation.

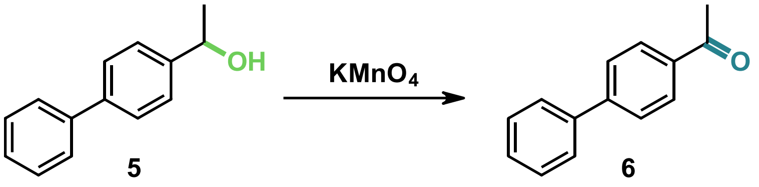

Scheme S-2. Oxidation of the secondary alcohol in 1-(4-biphenyl)ethanol **(5)** using $KMnO_4$ as oxidant.

3.1 General Procedure B

A 14 mL PFA milling vessel containing a single milling ball was charged with 1-(4-biphenyl)ethanol **(5)** [CAS: 3562-73-0] (46 mg, 0.25 mmol, 1 equiv.), potassium permanganate [CAS: 7722-64-7] (59 mg, 0.375 mmol, 1.5 equiv. or 79 mg, 0.5 mmol, 2 equiv.; specified for each experiment), and a defined amount of 1,2,3-trimethoxybenzene as an inert internal standard. The vessel was closed, mounted in the holding station of a Retsch MM500 vario, and milled for the specified time and frequency. Milling ball size, weight, frequency, and duration are detailed in the corresponding table entries. Upon completion of the reaction, $Na_2S_2O_3 \cdot 5\,H_2O$ (140–160 mg, >2 equiv.) and 400–500 mg celite were added. The vessel was closed again and milled at 30 Hz for 1 min to complete quenching of the oxidant. It should be noted that celite does not act as a quenching agent; its purpose is to facilitate subsequent NMR sample preparation by producing a powdery mixture rather than a sticky mass that complicates workup.

After quenching, the vessel was cooled to room temperature, and 1.5 mL $CDCl_3$ were added. The vessel walls were rinsed carefully with the solvent, and the mixture was filtered through a short plug of celite (packed in a cotton-stuffed Pasteur pipette) directly into an NMR tube. ^{1}H-NMR spectra were recorded with a prolonged relaxation delay (D1 = 5 s).

For details on FID processing, refer to the dedicated NMR analysis **Section 3.3**.

3.2 Reaction Validation and Preliminary Studies

In analogy to the halogen exchange reaction, several control experiments were conducted for the oxidation reaction to ensure that quenching was both instantaneous and complete, thereby excluding any significant conversion during (NMR) solvent addition or subsequent quantitative analysis.

Control I – Reaction progress with and without quencher under solvent-free conditions and in solution

Reactions were performed according to General Procedure B using a 15 mm ZrO_2 milling ball at 20 Hz for 5 min with 1.5 equiv. $KMnO_4$, with the following deviations: omission of 1,2,3-trimethoxybenzene as internal standard for **control I-1** to **I-5**, and omission of the quenching step for **control I-3** to **I-5**.

^{1}H-NMR spectra were recorded without internal standard addition; therefore, only relative starting material-to-product ratios (SM:P) are reported.

Control I-1: Quenching immediately after the reaction (General Procedure B). Immediate NMR analysis.
SM:P = 63:37.

Control I-2: Quenching immediately after the reaction, followed by addition of 1.5 mL $CDCl_3$ and standing for 20 min before NMR sample preparation (celite filtration).
SM:P = 66:34.

Control I-3: No quenching, fast NMR sample preparation.
SM:P = 56:44.

Control I-4: No quenching, addition of 1.5 mL $CDCl_3$, standing for 20 min before NMR sample preparation (celite filtration).
SM:P = 38:62.

Control I-5: No immediate quenching, vessel left standing for 20 min without milling, followed by quenching and NMR sample preparation.
SM:P = 44:56.

These results demonstrate that immediate quenching after the reaction is crucial to accurately quantify conversion under milling conditions. Without quenching, the reaction continues both in the presence and absence of solvent.

Control J – Verification of instantaneous and complete oxidant quenching

To effectively test whether the quenching step occurs instantaneously and completely, several reactions were performed in which the quencher ($Na_2S_2O_3$·5 H_2O, with or without additional celite) was present from the very beginning of the reaction. In theory, if the quenching process were slower than the oxidation itself, significant amounts of the alcohol would still be oxidized to the ketone **6** despite the presence of quencher, since the oxidant and substrate would coexist long enough for conversion to occur. In contrast, if the oxidant is quenched rapidly and fully, the starting alcohol **5** should remain unreacted and be quantitatively recovered. The comparison with and without additional celite was included to investigate whether the large bulk amount of celite could potentially slow down the quenching step by diluting or hindering the contact between oxidant and quencher.

Control J-1 and J-2:

A 14 mL PFA vessel containing a single 15 mm ZrO_2 milling ball was charged with 1-(4-biphenyl)ethanol **(5)** [CAS: 3562-73-0] (25 mg, 0.5 equiv.), acetyl-4-biphenyl **(6)** [CAS: 92-91-1] (25 mg, 0.5 equiv., deliberately added as product reference), potassium permanganate [CAS: 7722-64-7] (79 mg, 2 equiv.), and 1,2,3-trimethoxybenzene as an inert internal standard. In addition, $Na_2S_2O_3$·5 H_2O (140–160 mg, >2 equiv.) was added from the beginning for **Control J-1**, while for **Control J-2** the same amount of $Na_2S_2O_3$·5 H_2O was combined with an additional 400–500 mg of celite. The vessel was closed, mounted into the Retsch MM500 vario, and subjected to milling at 30 Hz for 2 min. Subsequent NMR sample preparation and analysis were carried out according to General Procedure B.

In both **Control J-1** and **J-2** experiments, the starting material 1-(4-biphenyl)ethanol **(5)** was recovered in 46% and 45%, respectively, while the deliberately added product acetyl-4-biphenyl **(6)** was quantified at 50% in both cases.

These findings confirm that the starting alcohol was not converted to aldehyde under the given conditions, but instead could be quantitatively recovered, thus proving that $Na_2S_2O_3{\cdot}5\,H_2O$ quenches $KMnO_4$ completely and rapidly. Importantly, the efficiency of the quenching was not influenced by the presence of large amounts of celite.

Control K – Exclusion of further reaction within the NMR tube

To exclude the possibility that the reaction continues after quenching and NMR sample preparation, a reaction was performed using a 15 mm ZrO_2 milling ball at 20 Hz for 5 min according to General Procedure B (1.5 equiv. $KMnO_4$). An NMR spectrum was recorded immediately, and the same NMR sample was re-recorded 5 h later. No change in the ratio of starting material to product was observed.

This experiment confirms that, after efficient quenching and sample preparation, no further reaction occurs in the NMR tube, validating quantitative NMR as a reliable method for yield determination.

Control L – Check product stability in the presence of oxidant

To examine whether the product acetyl-4-biphenyl **(6)** is stable in the presence of $KMnO_4$ and whether overoxidation of the ketone to the corresponding acid could occur, a reaction was performed according to General Procedure B with the modification that acetyl-4-biphenyl **(6)** (50 mg, 1 equiv.) was used as substrate instead of the alcohol starting material **(5)**. The reaction was conducted with $KMnO_4$ (79 mg, 2 equiv.). Quantitative NMR analysis confirmed full recovery of acetyl-4-biphenyl **(6)**, demonstrating that *the product remains completely stable under the applied milling and oxidation conditions*.

3.3 Quantitative NMR Analysis

After sample preparation according to General Procedure B, the NMR spectra were recorded on a Bruker 400 MHz spectrometer using a prolonged relaxation delay (D1) of 5 seconds to ensure reliable quantitative analysis.

The recorded spectra were subsequently processed using MestreNova software following the protocol below:

- Apodization along t_1: exponential, 0.5 Hz.
- Zero filling along t_1: from FID size to 524288 points (512K).
- Automatic phase correction: Global, Whitening, Regions, Selective, Min. Entropy, Metabonomics, and Baseline Optimization (initial phase = 0).
- Automatic baseline correction: ablative method, 5 points, 10 passes.

An example spectrum used for quantitative NMR analysis is shown below (**Figure S-2**), displaying only the relevant region of interest:

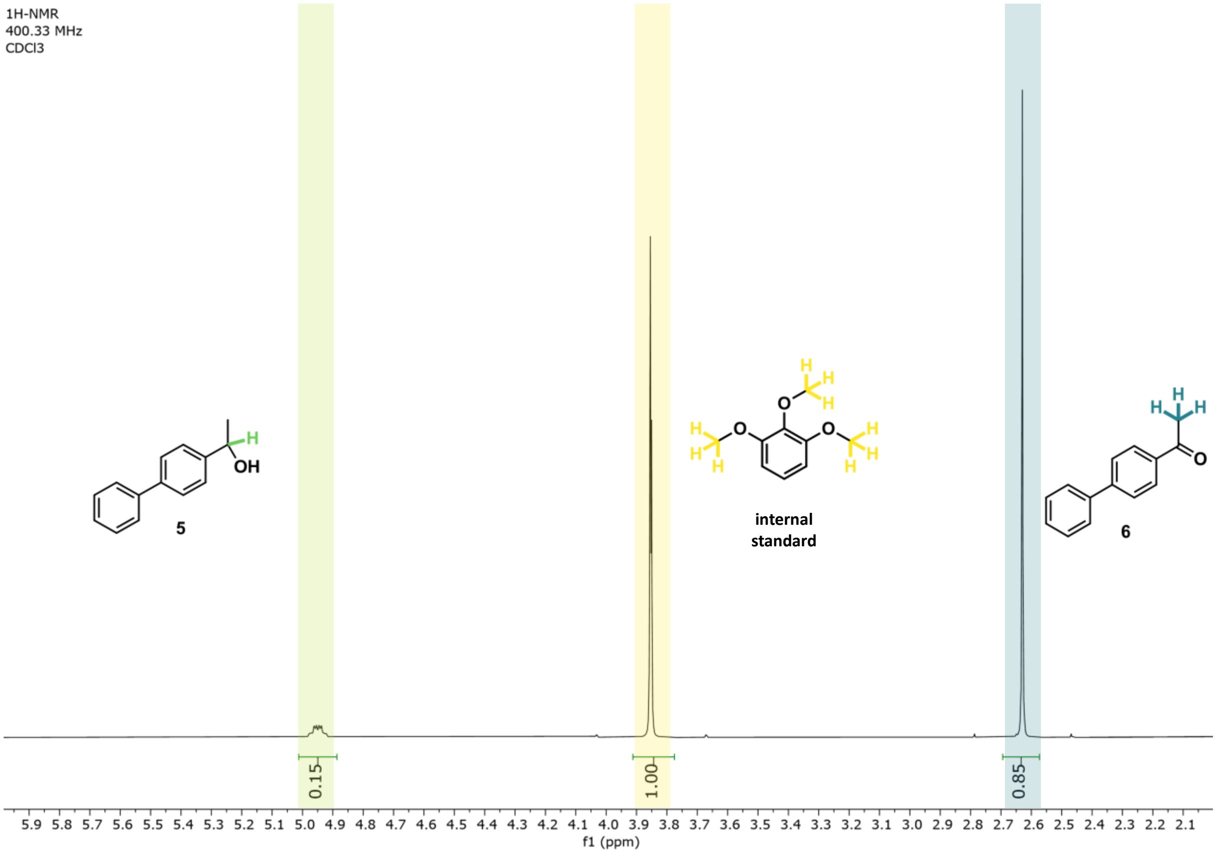

Figure S-2. Example ^{1}H-NMR spectra for quantitative analysis of the permanganate oxidation including the signal of the internal NMR standard.

3.4 Complete results of kinetic studies

In the following, the full set of results from the kinetic studies is presented. Unless stated otherwise, all reactions were conducted according to General Procedure B. The milling ball material and size, as well as the milling frequency and reaction time, are specified in the respective table entries. The single impact energy (E_{impact}) and total energy input (E_{total}) for each experiment were calculated following the report by the Lungerich group[1] using their online ball mill calculator for mixer mills (https://lungerich-group.github.io/Ball-mill-calculator/). The calculations were based on a system employing a single milling ball and the following jar dimensions:

- Jar diameter: 20 mm
- Jar length: 60 mm
- Amplitude of oscillation: 30 mm

The specific milling parameters (ball size [mm], ball weight [g], milling frequency [Hz], and milling time [min]) applied in the calculations can be found in the corresponding table entries. For all reactions showing incomplete conversion, only unreacted starting material **(5)** was detected in the crude mixtures. Quantitative recovery of the remaining starting material was confirmed by quantitative ^{1}H-NMR analysis, ruling out the formation of significant amounts of side products.

3.4.1 Yields at a Constant Milling Time of 5 min and Constant Frequency of 20 Hz Using Milling Balls of Different Materials and Masses (15 mm Diameter)

In analogy to the halogen exchange reaction described in **Section 2.4.3**, the oxidation reaction was likewise investigated to determine the dominant driving factor: whether conversion is primarily governed by kinetic energy delivered through mechanical impact forces, or whether the number of net impacts plays a more significant role.

All reactions were performed at a fixed milling time of 5 min and a constant frequency of 20 Hz, following General Procedure B, using 1.5 equivalents of $KMnO_4$ as oxidant. A single 15 mm milling ball was employed for each reaction, while the ball material was systematically varied, resulting in different ball weights across the experiments. The milling balls were made of tungsten carbide (WC), chrome steel (Fe–Cr), zirconia (ZrO_2), or silicon nitride (Si_3N_4). The complete ball specifications, along with the corresponding product yields for compound **6**, are summarized in the following table, with the data further visualized in **Chart S-17**.

Table S-17. Reaction parameters and yields of **6** for reactions ball-milled for a fixed reaction time of 5 min at a constant milling frequency of 20 Hz using 15 mm milling balls of different materials.

entry	ball size [mm]	ball material	material density [g/cm³]	ball mass [g]	milling time [min]	frequency [Hz]	calc. E_{impact} [J]	calc. E_{total} [J]	yield (6)	average yield (6) ± std. dev.
1	15	WC	14.90	26.226	5.00	20	0.186	10910	36%	
2	15	WC	14.90	26.234	5.00	20	0.186	10913	27%	33 ± 5%
3	15	WC	14.90	26.257	5.00	20	0.186	10923	36%	
4	15	Fe-Cr	7.70	13.553	5.00	20	0.096	4906	28%	
5	15	Fe-Cr	7.70	13.586	5.00	20	0.097	4820	21%	25 ± 3%
6	15	Fe-Cr	7.70	13.590	5.00	20	0.097	4821	26%	
7	15	ZrO_2	6.10	11.337	5.00	20	0.081	4715	10%	
8	15	ZrO_2	6.10	11.359	5.00	20	0.081	4716	19%	15 ± 5%
9	15	ZrO_2	6.10	11.348	5.00	20	0.081	4721	17%	
10	15	Si_3N_4	3.20	5.723	5.00	20	0.041	2381	4%	
11	15	Si_3N_4	3.20	5.741	5.00	20	0.041	2388	7%	5 ± 2%
12	15	Si_3N_4	3.20	5.762	5.00	20	0.041	2397	6%	

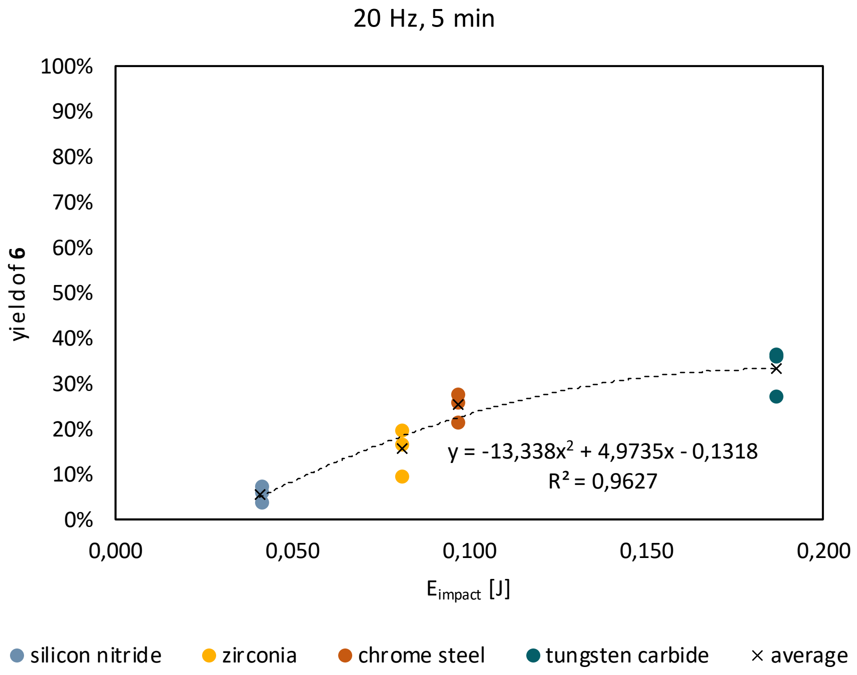

Chart S-17. Yields of **6** as a function of E_{impact} [J] for reactions performed by ball milling for a fixed time of 5 min at 20 Hz using 15 mm milling balls made from different materials. Black crosses indicate the average yield for each set of reactions conducted with identical E_{impact}, and the trendline represents the polynomic (2nd order) correlation of these averages.

In analogy to the previous findings for the halogen exchange reaction (see **Section 2.4.3**), similar trends are observed for the potassium permanganate oxidation of a secondary alcohol: the reaction is predominantly driven by mechanical forces, with higher kinetic energy leading to increased product yields, rather than being governed by the number of net impacts and how often the ball travels back-and-forth.

3.4.2 Yields at constant impact energy (E_{impact}) via ball mass and frequency variation at constant milling times

Several sets of experiments were performed at a constant milling time, with the milling frequency adjusted according to the milling ball weight to achieve a comparable theoretical single impact energy (E_{impact}), calculated according to Lungerich.[1] All reactions were carried out following General Procedure B using 2 equivalents of $KMnO_4$. The specific ball milling parameters and milling material specifications for each experiment are provided in the respective tables below.

Table S-18. Reaction parameters and yields of **6** for reactions ball milled for a fixed reaction time of 5 min with different frequencies adapted to the milling ball mass to maintain the single impact energy (E_{impact}) within a narrow range of 0.116–0.119 J.

entry	ball size [mm]	ball material	material density [g/cm³]	ball mass [g]	milling time [min]	frequency [Hz]	calc. E_{impact} [J]	calc. E_{total} [J]	yield (6)	average yield (6) ± std.dev.
1	15	WC	14.90	26.226	5.00	16	**0.119**	5586	30%	27 ± 3%
2	15	WC	14.90	26.234	5.00	16	**0.119**	5588	25%	
3	15	WC	14.90	26.257	5.00	16	**0.119**	5593	24%	
4	15	Fe-Cr	7.70	13.553	5.00	22	**0.117**	7504	28%	27 ± 1%
5	15	Fe-Cr	7.70	13.586	5.00	22	**0.117**	7523	27%	
6	15	Fe-Cr	7.70	13.590	5.00	22	**0.117**	7525	26%	
7	15	ZrO_2	6.10	11.333	5.00	24	**0.116**	8147	35%	32 ± 7%
8	15	ZrO_2	6.10	11.337	5.00	24	**0.116**	8150	24%	
9	15	ZrO_2	6.10	11.348	5.00	24	**0.116**	8158	36%	
10	15	Si_3N_4	3.20	5.723	5.00	34	**0.118**	11697	49%	44 ± 5%
11	15	Si_3N_4	3.20	5.741	5.00	34	**0.118**	11734	46%	
12	15	Si_3N_4	3.20	5.762	5.00	34	**0.118**	11777	38%	

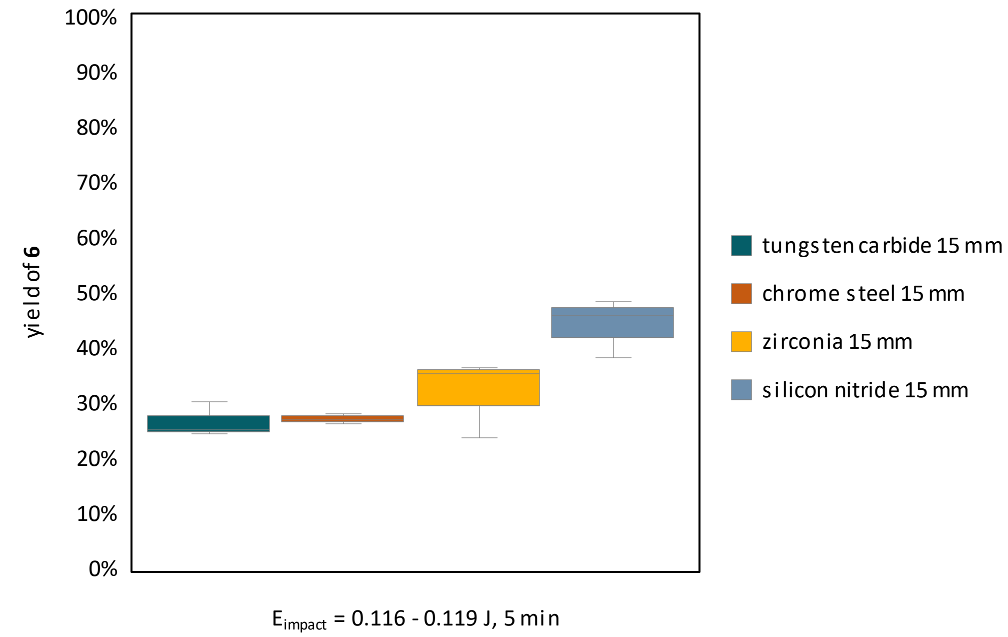

Chart S-18. Yields of **6** for reactions ball milled for a fixed reaction time of 5 min with different frequences adapted for the milling ball mass to keep the single impact energy (E_{impact}) in a narrow range of 0.116 – 0.119 J.

3.4.3 Yields at Comparable E_{impact} via Frequency Adjustment for Ball Milling Masses with Total Energy (E_{total}) Equalized by Adjusting Milling Times to Standardize the Number of Theoretical Impacts

Analogous to the procedure described in **Section 2.4.5**, the reaction times of the target experiment were adjusted ($t_{adjusted}$) with respect to a reference experiment conducted at a reference frequency ($f_{reference}$) for a reference milling time ($t_{reference}$) via a frequency ratio (FR), in order to level the number of impacts of the target experiment to that of the reference experiment according to Eq. 2 and 3.

In the following, the reference experiment is color-coded in green within table and the corresponding chart. The times displayed in the tables correspond to the adjusted times ($t_{adjusted}$) calculated via the equations provided in **Section 2.4.5**. All reactions were performed following the general procedure B using 2 equiv. of $KMnO_4$. Reaction and milling parameters are provided in the tables below.

It should be noted that at 16 Hz, using a relatively heavy tungsten carbide milling ball, the ball movement is somewhat reluctant and inert at these low frequencies, likely resulting in significantly reduced net impact events.

When the results for E_{impact} = 0.116-0.119 and E_{total}-equalized reactions in the oxidation system are compared with those of the halogen exchange reaction (see **Section 2.4.5**), two main conclusions can be drawn:

In both the halogen exchange reaction and the oxidation reaction, equalizing E_{total} by adjusting the reaction time could not fully equilibrate the yields. Prolonging the reaction times for low-frequency reactions resulted in an "overshooting" of product yields relative to the reference experiment. This effect was more pronounced for the halogen exchange reaction, where yields tended to increase substantially for significantly prolonged reaction times (e.g., 16 Hz, 21 min 15 sec for tungsten carbide in **Table S-12 entries 1–3**). For the oxidation reaction, however, the yield differences were less significant, and a reverse trend was observed: the longer the adjusted milling time, the closer the yield approached that of the reference experiment (cf. **Table S-19 entries 1–3**).

Table S-19. Reaction parameters and yields of **6** for reactions with comparable E_{impact} and E_{total}. Reference experiments are shown in entries 10–12 (green); for all other entries, the milling times were adjusted to equalize the number of impacts relative to the reference experiment.

entry	ball size [mm]	ball material	material density [g/cm³]	ball mass [g]	milling time [min]	frequency [Hz]	calc. E_{impact} [J]	calc. E_{total} [J]	yield (6)	average yield (6) ± std. dev
1	15	WC	14.90	26.226	10.63	16	**0.119**	11876	47%	46 ± 3%
2	15	WC	14.90	26.234	10.63	16	**0.119**	11879	48%	
3	15	WC	14.90	26.257	10.63	16	**0.119**	11890	43%	
4	15	Fe-Cr	7.70	13.553	7.73	22	**0.117**	11602	55%	55 ±4%
5	15	Fe-Cr	7.70	13.586	7.73	22	**0.117**	11630	59%	
6	15	Fe-Cr	7.70	13.590	7.73	22	**0.117**	11633	51%	
7	15	ZrO_2	6.10	11.333	7.08	24	**0.116**	11536	64%	61 ± 5%
8	15	ZrO_2	6.10	11.337	7.08	24	**0.116**	11540	55%	
9	15	ZrO_2	6.10	11.348	7.08	24	**0.116**	11551	65%	
10	15	Si_3N_4	3.20	5.723	5.00	34	**0.118**	11697	49%	44 ± 5%
11	15	Si_3N_4	3.20	5.741	5.00	34	**0.118**	11734	46%	
12	15	Si_3N_4	3.20	5.762	5.00	34	**0.118**	11777	38%	

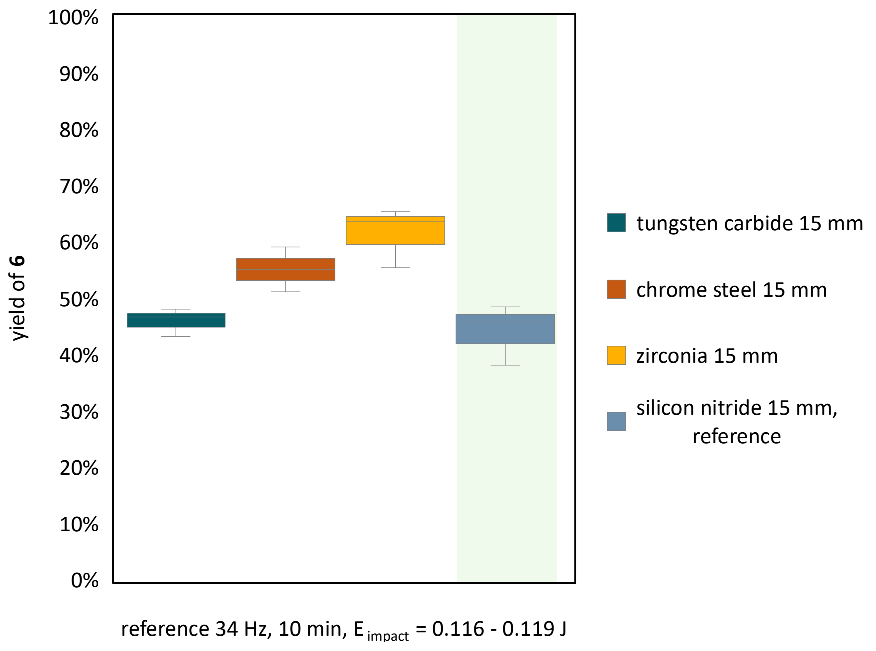

Chart S-19. Yields of **6** for reactions with comparable E_{impact} and E_{total}. Reference experiments (silicon nitride, 15 mm) are displayed in the rightmost column (4th box); for all other entries, the milling times were adjusted to equalize the number of impacts relative to the reference experiment.

These results must be carefully interpreted, as increasing amounts of MnO_2, formed during the $KMnO_4$ oxidation, gradually moderate the reaction. This effect can inherently prevent "overshooting" in yields, potentially triggering the misleading assumption that a constant E_{impact} and E_{total} is sufficient as a comparison and interconversion parameter in the oxidation system. Consequently, $KMnO_4$ oxidation of secondary alcohols is not considered entirely suitable for investigating the total energy and single impact energy theory.

Nonetheless, the oxidation reactions confirm that this all-solid transformation is again driven primarily by kinetic energy and mechanical impact, rather than by particle translocation alone, and that the energy of the single impact (E_{impact}) is indeed a critical factor directly influencing reaction kinetics.

Control reactions corroborating the moderating effect of MnO_2 formed during the oxidation are provided in the subsequent section.

3.4.4 Moderation of the oxidation reaction by MnO_2

To demonstrate that the oxidation reaction is significantly slowed in the presence of MnO_2, a by-product formed during the reaction, a set of control experiments was performed in which either no MnO_2 or defined amounts of MnO_2 (1 equiv. and 2 equiv.) were added at the start of the reaction, prior to milling.

All reactions were conducted following General Procedure B using 2 equiv. $KMnO_4$, with the deviation that either no MnO_2 (**controls M1–M3**), 1 equiv. MnO_2 (**controls M4–M6**) or 2 equiv. (**controls M7–M9**) MnO_2 were added prior to milling. Milling was performed using a single 15 mm ZrO_2 ball at a frequency of 24 Hz for 5 minutes.

Table S-20. Control-M experiments demonstrating the effect of MnO_2 on the oxidation reaction yields.

experiment	frequency [Hz]	milling time [min]	MnO_2 equiv.	yield (6)	average yield (6)	Δ yield (6) from rxn without MnO_2
**control-M1**	20	5	-	35%		-
**control-M2**	20	5	-	24%	32 ± 7%	-
**control-M3**	20	5	-	36%		-
**control-M4**	20	5	**1**	17%		**15%**
**control-M5**	20	5	**1**	24%	22 ± 5%	**8%**
**control-M6**	20	5	**1**	25%		**7%**
**control-M7**	20	5	**2**	13%		**19%**
**control-M8**	20	5	**2**	15%	13 ± 2%	**17%**
**control-M9**	20	5	**2**	11%		**20%**

These results clearly demonstrate that increasing amounts of MnO_2 slow down the oxidation reaction, leading to significantly lower yields when MnO_2 is present from the start, compared to reactions without added MnO_2.

MnO_2 is a known oxidant for benzylic alcohols. In the present system, MnO_2 is generated *in situ* as the reduction product of permanganate, and its effect on the rate is governed by its kinetics relative to the permanganate pathway. The observed inhibition can be rationalized as follows: **(A)** The alcohol can interact with both $KMnO_4$ and MnO_2, but oxidation *via* the permanganate ester is much faster than oxidation at MnO_2. **(B)** As MnO_2 accumulates, a progressively larger fraction of the substrate enters this slower MnO_2-mediated oxidation pathway, reducing the flux through the faster permanganate pathway and thereby decreasing the overall rate. **(C)** Interactions between MnO_2 and permanganate can further lower the effective concentration of active permanganate, reinforcing the inhibitory effect.

Thus, the rate decrease is explained by differences in reaction kinetics rather than by oxidizing capability: although MnO_2 can oxidize benzylic alcohols, the permanganate pathway is substantially faster, and the build-up of MnO_2 diverts substrate into a slower oxidation channel, leading to reduced overall reaction rate.

4. The Wittig Olefination under All-Solid and LAG Conditions

To investigate a mechanochemical transformation fundamentally different from the halogen exchange, we selected the Wittig olefination under ball-milling conditions, which has been previously reported in the literature.[3-4] In the original protocol, the reaction employs KO^{t}Bu as a solid base. During the process, KO^{t}Bu is consumed, generating ^{t}BuOH in situ, which can act as a liquid-assisted grinding (LAG) additive. Under such conditions, the reaction is extremely fast (e.g., aldehyde **3** methylenation in 30 seconds). However, the progressive in situ generation of ^{t}BuOH means that the amount of liquid present cannot be precisely controlled, making this protocol unsuitable for systematic kinetic investigations. To overcome this limitation, we employed Cs_2CO_3 as the solid base, which allows the reaction to be conducted either under strictly solvent-free all-solid conditions or with the addition of a defined amount of ^{t}BuOH as LAG. Under these conditions, the reaction is somewhat slower but still complete within a suitable timeframe (<30 min) for kinetic studies. Using biphenyl-4-carboxaldehyde **(3)** as starting material, the reaction affords 4-vinylbiphenyl **(4)** with triphenylphosphine oxide as a solid byproduct. Thus, all components except the optional LAG additive remain solid, ensuring precise control over the amount of liquid present in the reaction mixture.

As the underlying mechanism of this reaction is fundamentally different from the previously investigated halogen exchange reaction (see **Section 2**), the Wittig olefination is well-suited to further examine the roles of E_{impact} and E_{total} on reaction kinetics, both with and without LAG additives. The most striking difference of the Wittig olefination compared to the halogen exchange is the absence of observable aging. While the halogen exchange reaction exhibited a small but measurable degree of aging—defined as the continued conversion of solid reactants after milling in the absence of further mechanical impact—the Wittig olefination showed no such effect. This indicates that, in contrast to the halogen exchange, the Wittig reaction either (i) proceeds exclusively during mechanical impact events and ceases immediately when mechanical forces are absent, or (ii) undergoes any potential post-impact conversion so rapidly and completely that it cannot be detected on a macroscopic timescale. In practice, this makes the reaction appear as if conversion occurs exclusively under direct mechanical impact.

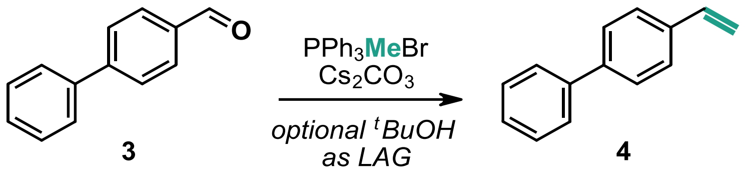

Scheme S-3. The Wittig olefination of biphenyl-4-carboxaldehyde **(3)** to 4-vinylbiphenyl **(4)**

To probe the role of liquid content, we systematically varied the amount of *t*-BuOH from low η-values (0.1–0.5 µL·mg^{-1}, typical for LAG) to higher η-values of 1.0 (entering the "slurry" regime). This allowed us to investigate whether the reaction remains impact-driven or whether, at higher liquid content, diffusion likely begins to dominate as the main driving force. It should be emphasized that ^{t}BuOH is not a solvent typically employed for Wittig olefinations and is therefore not expected to accelerate the reaction in the same way as commonly used polar aprotic solvents such as THF or diethyl ether. Nonetheless, ^{t}BuOH should be capable of dissolving all reaction components to some degree, thereby potentially minimizing effects associated with solid-state surface activation. Using THF or diethyl ether as LAG agents would most likely lead to the same issue encountered in the halogen exchange reaction with acetone as LAG (**Section 2.2, Control A-3**): the reaction could proceed, at least in part, without mechanical impact and/or during NMR solvent addition, thereby rendering the system unsuitable for meaningful kinetic investigations.

4.1 General Procedure C

A 14 mL PFA vessel containing a single milling ball was charged with biphenyl-4-carboxaldehyde **(3)** [CAS: 3218-36-8] (36 mg, 0.2 mmol, 1 equiv.), methyl triphenylphosphonium bromide [CAS: 1779-49-3] (86 mg, 0.24 mmol, 1.2 equiv.), and Cs_2CO_3 [CAS: 534-17-8] (92 mg, 0.28 mmol, 1.4 equiv.). For experiments with LAG additives, a defined amount of ^{t}BuOH [CAS: 75-65-0] was added *via* microsyringe. The vessel was closed and mounted into the holding station of a Retsch MM500 vario, and milling was conducted for the specified time and frequency. The milling ball size, weight, frequency, and milling time are detailed in the respective table entries.

Temperature measurements were taken immediately after milling using an infrared thermometer to record the vessel wall temperature.

After cooling the vessel to room temperature, 1 mL $CDCl_3$ and 20 µL dibromomethane [CAS: 74-95-3] were added as an internal NMR standard *via* microsyringe. The vessel walls were carefully rinsed with the solvent, and the mixture was filtered over a short plug of celite (in a cotton-stuffed Pasteur pipette) directly into an NMR tube. A ^{1}H-NMR spectrum was recorded with a prolonged relaxation delay (D1) of 5 seconds.

4.2 Reaction validation and preliminary studies

Control N – Check the necessity of quenching the base and exclude reaction progress for LAG reactions upon $CDCl_3$ addition

The reaction was performed following the general procedure C with 21 µL ^{t}BuOH as LAG using a 15 mm ZrO_2 milling ball at 20 Hz for 1 minute (**control-N3** and **control-N4**). For **control-N1** and **control-N2** a quenching step was added immediately after milling: the vessel was opened, 2 equiv. NH_4Cl were added, and the reaction was milled again for 1 min at 30 Hz. After this quench, the sample was prepared for quant. ^{1}H-NMR measurements as described in the General Procedure C.

Control-N1: The NMR sample was prepared and immediately measured after the reaction.
The product was obtained in **9%** yield

Control-N2: After the reaction, 1 mL $CDCl_3$ was added, and the mixture was left to stand for 30 minutes before adding CH_2Br_2 and preparing the sample for NMR measurement.
The product was obtained in **6%** yield.

Control-N3: No quenching step. The NMR sample was prepared and immediately measured after the reaction.
The product was obtained in **9%** yield.

Control-N4: No quenching step. After the reaction, 1 mL $CDCl_3$ was added, and the mixture was let stand for 30 minutes before adding CH_2Br_2 and preparing the sample for NMR measurement.
The product was obtained in **3%** yield.

These results demonstrate that the reaction does not proceed in $CDCl_3$, neither with nor without an additional quenching step. For practical reasons, the acidic quench was omitted in all subsequent olefination kinetic studies.

Control O – Exclude conversion without mechanical impact

To test whether the reaction proceeds without mechanical impact, the solid reactants were placed in a 5 mL glass vial without LAG (**control-O1**) and with 21 µL ^{t}BuOH (**control-O2**). Subsequently, 1 mL $CDCl_3$ and 20 µL CH_2Br_2 (internal standard) were added, and the mixtures were stirred for approximately 5 minutes. The mixtures were filtered over a short plug of celite directly into NMR tubes. No conversion was observed in either control; only starting material was recovered.

These experiments indicated that the reaction is indeed necessitating mechanical forces to effectively proceed.

Control P – Aging experiments

Aging experiments were conducted to evaluate whether product forms after stopping the milling process and letting the sample stand for a defined period ("aging time") in the dry, all-solid state without mechanical impact or solvent addition. The effect of aging on reaction yield was determined for an aging time of 1 h. For each aging experiment, a pair of identical reactions was set up following General Procedure C with 21 µL ^{t}BuOH as LAG. One reaction was immediately prepared for quantitative ^{1}H-NMR analysis, while the second sample was left standing for 1 h after milling stopped, without any mechanical impact or solvent addition, before NMR analysis. All reactions used 15 mm ZrO_2 milling balls at 25 Hz for 10 minutes.

The results indicate that, in the absence of mechanical impact or bulk solvent, the olefination reaction does not proceed, in contrast to the halogen exchange reaction, which shows slow aging.

Table S-21. Reaction parameters and yields of **4** for the aging-experiments.

entry	frequency [Hz]	milling time [min]	aging time [h]	yield (4)	average yield (4)
1	25	10	-	31%	
2	25	10	-	31%	32 ± 2%
3	25	10	-	35%	
4	25	10	**1**	31%	
5	25	10	**1**	30%	32 ± 3%
6	25	10	**1**	35%	

4.3 Quantitative NMR analysis

After sample preparation as described in the General Procedure C, the ^{1}H-NMR spectra were recorded on a Bruker 400 MHz machine with a prolonged relaxation delay (D1) of 5 seconds.

The recorded spectra were subsequently processed using MestreNova software following the protocol below:

- Apodization along t_1: exponential, 0.5 Hz.
- Zero filling along t_1: from FID size to 524288 points (512K).
- Automatic phase correction: Global, Whitening, Regions, Selective, Min. Entropy, Metabonomics, and Baseline Optimization (initial phase = 0).
- Automatic baseline correction: ablative method, 5 points, 10 passes.

An example spectrum used for quantitative NMR analysis is shown below (**Figure S-3**), displaying only the relevant region of interest:

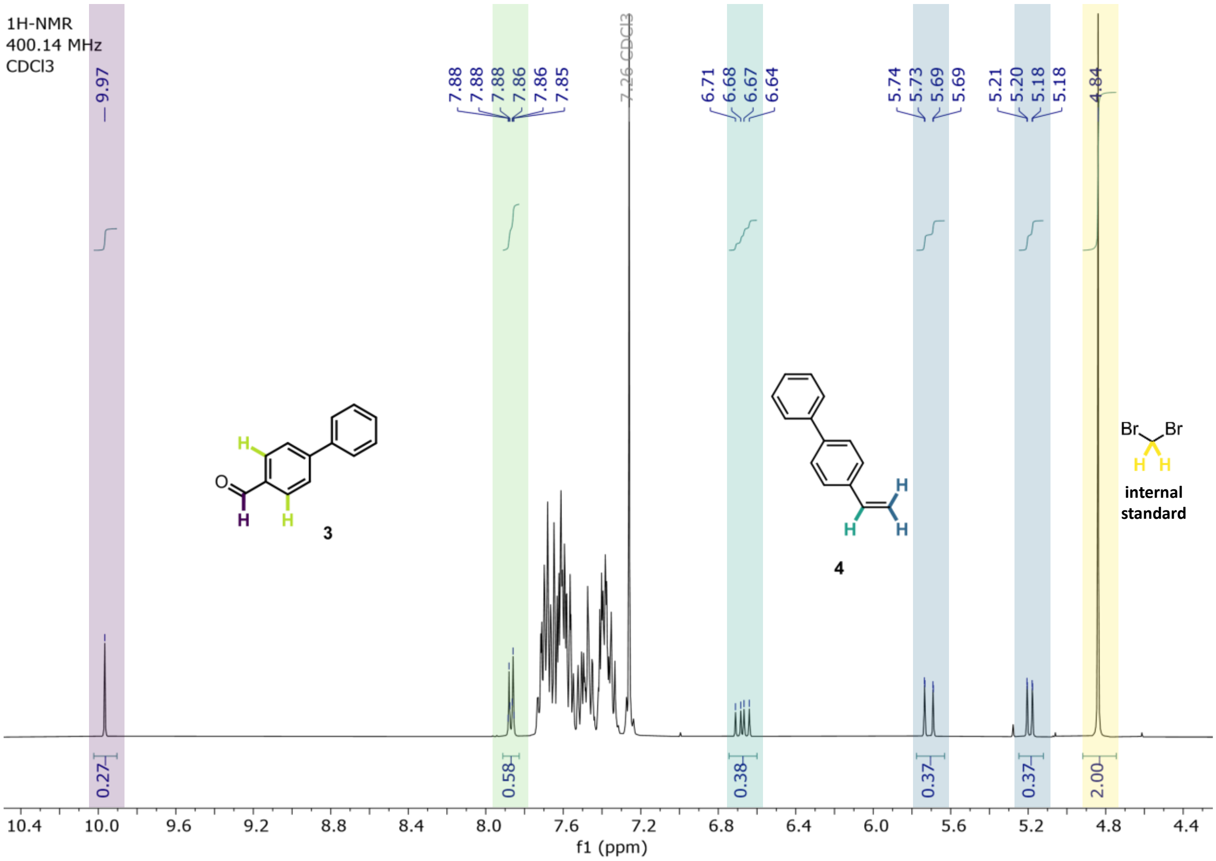

Figure S-3. Example ^{1}H-NMR spectra for quantitative analysis of the Wittig olefination including the signal of the internal NMR standard.

4.4 Complete results of kinetic studies

In the following, the full results from the kinetic studies are presented. Unless stated otherwise, all reactions were conducted according to General Procedure C. The milling ball material and size, as well as the milling frequency and reaction time, are specified in the respective table entries. The single impact energy (E_{impact}) and total energy input (E_{total}) for each experiment were calculated following the report by the Lungerich group[1] using their online ball mill calculator for mixer mills (https://lungerich-group.github.io/Ball-mill-calculator/). The calculations were based on a system employing a single milling ball and the following jar dimensions:

- Jar diameter: 20 mm
- Jar length: 60 mm
- Amplitude of oscillation: 30 mm

The specific milling parameters (ball size [mm], ball weight [g], milling frequency [Hz], and milling time [min]) applied in the calculations can be found in the corresponding table entries.

4.4.1 Milling Time-Dependent Yield at Defined Milling Frequencies

As the Wittig olefination neither showed observable aging effects nor significant self-moderation by reaction by-products, we hypothesized that the yield could be linearly dependent on the cumulative energy (E_{total}) in the system. To test this assumption, several olefination reactions were carried out at different frequencies and defined milling times, chosen such that the cumulative energy ranged between 5 000 and 25 000 J. The Wittig olefinations showed an almost perfectly linear correlation when the yield of **4** plotted against cumulative energy (E_{total}). This stands in clear contrast to the halogen-exchange reaction (cf. **Section 2.4.1, Chart S-2** and **Chart S-3**), where reactions with similar E_{total} showed substantial deviations, with prolonged milling times generally resulting in higher yields - a phenomenon attributed to slight but accumulating aging effects. In the Wittig olefination, the absence of such aging appears to allow for a direct correlation between reaction yield and cumulative energy in the system, provided that the activation energy threshold is overcome by the individual impact energy (E_{impact}).

Table S-22. Milling time-dependent yields of product 4 using a single 15 mm zirconia milling ball at the specified frequency.

entry	ball size [mm]	ball material	ball density [g/cm³]	ball mass [g]	milling time [min]	frequency [Hz]	calc. E_{impact} [J]	calc. E_{total} [J]	temperature [°C]	yield (4)	average yield (4) ± std. dev.
					35 Hz						
1	15	ZrO_2	6.10	11.300	2.5	35	0.246	12597		50%	
2	15	ZrO_2	6.10	11.300	2.5	35	0.246	12597	35	31%	43 ± 10%
3	15	ZrO_2	6.10	11.300	2.5	35	0.246	12597		48%	
					30 Hz						
4	15	ZrO_2	6.10	11.300	2.5	30	0.181	7933		29%	
5	15	ZrO_2	6.10	11.300	2.5	30	0.181	7933	32	24%	25 ± 4%
6	15	ZrO_2	6.10	11.300	2.5	30	0.181	7933		22%	
7	15	ZrO_2	6.10	11.300	5	30	0.181	15865		58%	
8	15	ZrO_2	6.10	11.300	5	30	0.181	15865	36	43%	53 ± 9%
9	15	ZrO_2	6.10	11.300	5	30	0.181	15865		59%	
10	15	ZrO_2	6.10	11.300	7.5	30	0.181	23798		78%	
11	15	ZrO_2	6.10	11.300	7.5	30	0.181	23798	38	71%	75 ± 4%
12	15	ZrO_2	6.10	11.300	7.5	30	0.181	23798		75%	

entry	ball size [mm]	ball material	ball density [g/cm³]	ball mass [g]	milling time [min]	frequency [Hz]	calc. E_{impact} [J]	calc. E_{total} [J]	temperature [°C]	yield (4)	average yield (4) ± std. dev.
**25 Hz**											
13	15	ZrO_2	6.10	11.300	5	25	0.125	9181		21%	
14	15	ZrO_2	6.10	11.300	5	25	0.125	9181	34	28%	26 ± 4%
15	15	ZrO_2	6.10	11.300	5	25	0.125	9181		28%	
16	15	ZrO_2	6.10	11.300	7.5	25	0.125	13772		44%	
17	15	ZrO_2	6.10	11.300	7.5	25	0.125	13772	37	42%	43 ± 1%
18	15	ZrO_2	6.10	11.300	7.5	25	0.125	13772		44%	
19	15	ZrO_2	6.10	11.300	10	25	0.125	18363		58%	
20	15	ZrO_2	6.10	11.300	10	25	0.125	18363	40	55%	55 ± 2%
21	15	ZrO_2	6.10	11.300	10	25	0.125	18363		54%	
22	15	ZrO_2	6.10	11.300	12.5	25	0.125	22953		63%	
23	15	ZrO_2	6.10	11.300	12.5	25	0.125	22953	39	70%	69 ± 6%
24	15	ZrO_2	6.10	11.300	12.5	25	0.125	22953		74%	
**20 Hz**											
25	15	ZrO_2	6.10	11.300	7.5	20	0.080	7051		17%	
26	15	ZrO_2	6.10	11.300	7.5	20	0.080	7051	31	20%	19 ± 2%
27	15	ZrO_2	6.10	11.300	7.5	20	0.080	7051		21%	
28	15	ZrO_2	6.10	11.300	10	20	0.080	9402		19%	
29	15	ZrO_2	6.10	11.300	10	20	0.080	9402	34	22%	22 ± 3%
30	15	ZrO_2	6.10	11.300	10	20	0.080	9402		24%	
31	15	ZrO_2	6.10	11.300	12.5	20	0.080	11752		26%	
32	15	ZrO_2	6.10	11.300	12.5	20	0.080	11752	36	30%	29 ± 3%
33	15	ZrO_2	6.10	11.300	12.5	20	0.080	11752		32%	
34	15	ZrO_2	6.10	11.300	15	20	0.080	14103		32%	
35	15	ZrO_2	6.10	11.300	15	20	0.080	14103	35	34%	34 ± 3%
36	15	ZrO_2	6.10	11.300	15	20	0.080	14103		37%	
**15 Hz**											
37	15	ZrO_2	6.10	11.300	15	15	0.045	5950		12%	
38	15	ZrO_2	6.10	11.300	15	15	0.045	5950	30	15%	15 ± 3%
39	15	ZrO_2	6.10	11.300	15	15	0.045	5950		17%	

Table S-23. Average yields of **4** from triplicate reactions as a function of the milling time and the calculated total energies (E_{total}) for each reaction set, grouped by milling frequencies.

time [min]	average yield (4)	calculated E_{total} [J]	average yield (4)	calculated E_{total} [J]	average yield (4)	calculated E_{total} [J]	average yield (4)	calculated E_{total} [J]	average yield (4)	calculated E_{total} [J]
	35		30		25		20		15	
**2.5**	43 ± 10%	12597	25 ± 4%	7933	-	-	-	-	-	-
**5**	-	-	53 ± 9%	15865	26 ± 4%	9181	-	-	-	-
**7.5**	-	-	75 ± 4%	23798	43 ± 1%	13772	19 ± 2%	7051	-	-
**10**	-	-	-	-	55 ± 2%	18363	22 ± 3%	9402	-	-
**12.5**	-	-	-	-	69 ± 6%	22953	29 ± 3%	11752	-	-
**15**	-	-	-	-	-	-	34 ± 3%	14103	15 ± 3%	5950

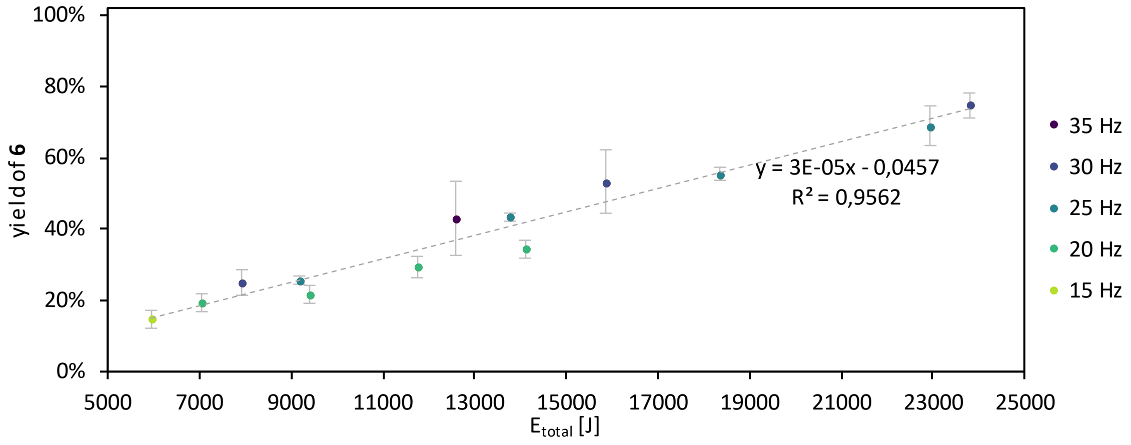

Chart S-20. Average yields of **4** from triplicate reaction as a function of milling time, plotted against the calculated total energy (E_{total}= 5 000-25 000 J)

4.4.2 Yield at a Calculated E_{total} of 12000 J and 18360 J at Selected Frequencies *via* Milling Time Variation

Analogous to the experiments in which E_{total} was kept constant for the all-solid halogen exchange reaction (see **Section 2.4.2**), several Wittig olefination reactions were conducted using a previously determined milling time for each frequency. This was done to achieve a theoretical E_{total} of 12 000 J for reactions without LAG and 18 360 J for reactions with 21 µL ^{t}BuOH as the LAG agent. The calculated E_{impact} values, as well as the corresponding milling times, are listed in the table below. The temperature inside the milling vessel was measured immediately after the milling process using an infrared thermometer, and the mean temperature for each set of three identical reactions is reported in the table.

No LAG, E_{total} = 12 000 J

Table S-24. Reaction parameters and yields of **4** for reactions with a calculated E_{total} of 12 000 J under all-solid conditions at the specified milling frequencies, with milling times adjusted accordingly.

entry	ball size [mm]	ball material	material density [g/cm³]	ball mass [g]	milling time [min]	frequency [Hz]	calc. E_{impact} [J]	calc. E_{total} [J]	temperature [°C]	yield (4)	average yield (4) ± std. dev.
1	15	ZrO_2	6.1	11.054	**2.43**	35	0.241	**12000**		28%	
2	15	ZrO_2	6.1	11.063	**2.43**	35	0.241	**12000**	35	28%	31 ± 5%
3	15	ZrO_2	6.1	11.075	**2.43**	35	0.241	**12000**		37%	
4	15	ZrO_2	6.1	11.123	**3.84**	30	0.178	**12000**		41%	
5	15	ZrO_2	6.1	11.125	**3.84**	30	0.178	**12000**	36	33%	37 ± 4%
6	15	ZrO_2	6.1	11.132	**3.84**	30	0.178	**12000**		38%	
7	15	ZrO_2	6.1	11.172	**6.60**	25	0.124	**12000**		30%	
8	15	ZrO_2	6.1	11.186	**6.60**	25	0.124	**12000**	34	33%	34 ± 5%
9	15	ZrO_2	6.1	11.195	**6.60**	25	0.124	**12000**		39%	
10	15	ZrO_2	6.1	11.333	**12.72**	20	0.081	**12000**		23%	
11	15	ZrO_2	6.1	11.340	**12.72**	20	0.081	**12000**	32	29%	25 ± 3%
12	15	ZrO_2	6.1	11.348	**12.72**	20	0.081	**12000**		23%	
13	15	ZrO_2	6.1	11.361	**30.06**	15	0.045	**12000**		25%	
14	15	ZrO_2	6.1	11.382	**30.06**	15	0.045	**12000**	31	25%	23 ± 3%
15	15	ZrO_2	6.1	11.389	**30.06**	15	0.045	**12000**		20%	

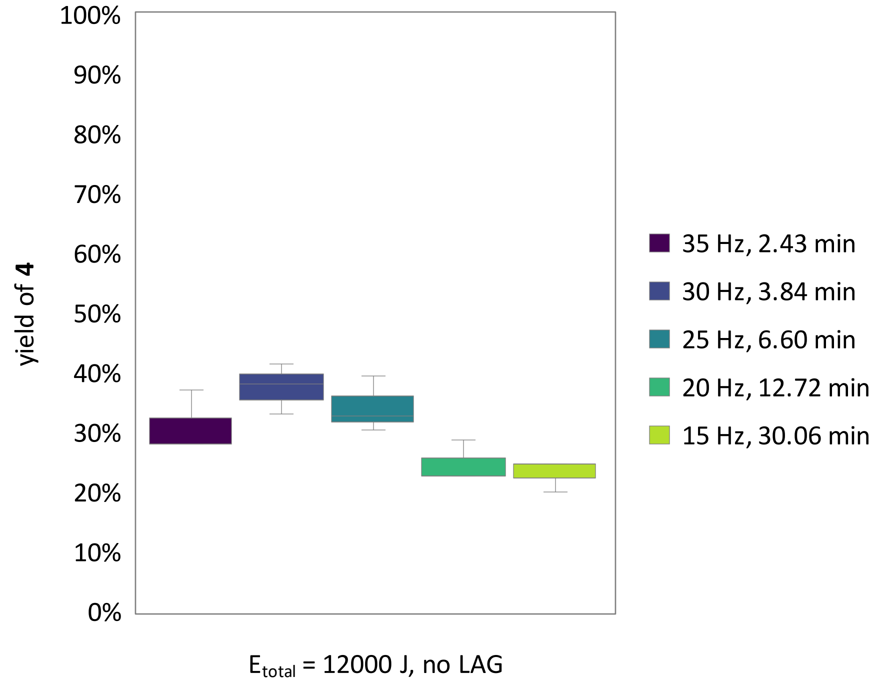

Chart S-21. Yields of **4** for reactions with a calculated E_{total} of 12 000 J under all-solid conditions at the specified milling frequencies, with milling times adjusted accordingly.

21 µL ^{t}BuOH as LAG, E_{total} = 18 360 J

Table S-25. Reaction parameters and yields of **4** for reactions with a calculated E_{total} of 18 360 J under LAG conditions (21 µL ^{t}BuOH) at the specified milling frequencies, with milling times adjusted accordingly.

**entry**	**ball size** [mm]	**ball material**	**material density** [g/cm³]	**ball mass** [g]	**milling time** [min]	**frequency** [Hz]	**calc.** E_{impact} [J]	**calc.** E_{total} [J]	**temperature** [°C]	**yield (4)**	**average yield (4)** ± std. dev.
1	15	ZrO_2	6.1	11.054	**3.72**	35	0.241	**18360**		38%	
2	15	ZrO_2	6.1	11.063	**3.72**	35	0.241	**18360**	34	43%	39 ± 3%
3	15	ZrO_2	6.1	11.075	**3.72**	35	0.241	**18360**		38%	
4	15	ZrO_2	6.1	11.123	**5.88**	30	0.178	**18360**		37%	
5	15	ZrO_2	6.1	11.125	**5.88**	30	0.178	**18360**	34	41%	39 ± 2%
6	15	ZrO_2	6.1	11.132	**5.88**	30	0.178	**18360**		40%	
7	15	ZrO_2	6.1	11.172	**10.10**	25	0.124	**18360**		31%	
8	15	ZrO_2	6.1	11.186	**10.10**	25	0.124	**18360**	34	31%	33 ± 3%
9	15	ZrO_2	6.1	11.195	**10.10**	25	0.124	**18360**		35%	
10	15	ZrO_2	6.1	11.333	**19.47**	20	0.081	**18360**		37%	
11	15	ZrO_2	6.1	11.340	**19.47**	20	0.081	**18360**	30	35%	37 ± 2%
12	15	ZrO_2	6.1	11.348	**19.47**	20	0.081	**18360**		39%	
13	15	ZrO_2	6.1	11.361	**45.96**	15	0.045	**18360**		35%	
14	15	ZrO_2	6.1	11.382	**45.96**	15	0.045	**18360**	31	35%	35 ± 1%
15	15	ZrO_2	6.1	11.389	**45.96**	15	0.045	**18360**		36%	

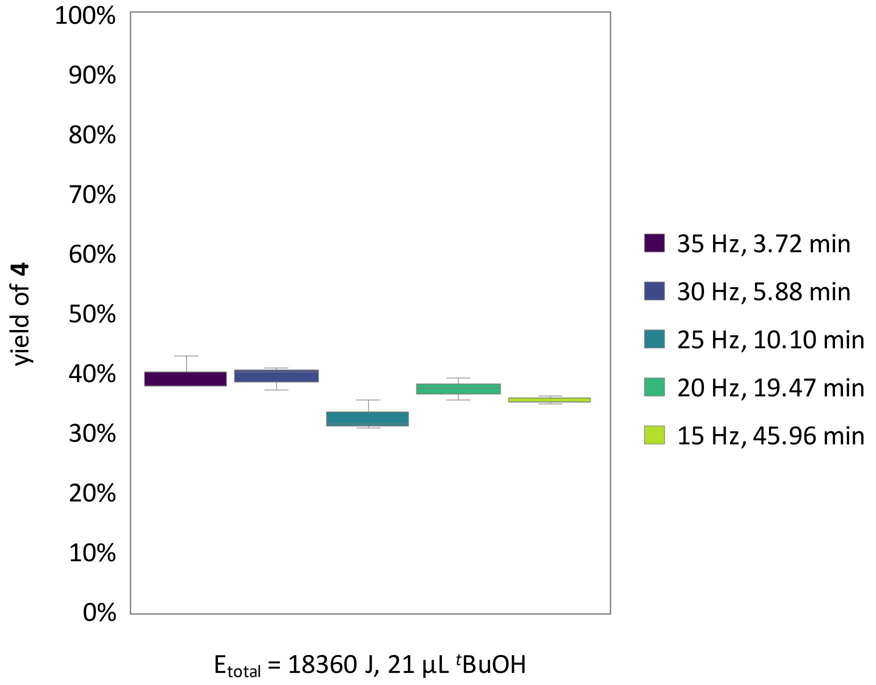

Chart S-22. Yields of **4** for reactions with a calculated E_{total} of 18 360 J under LAG conditions (21 µL ^{t}BuOH) at the specified milling frequencies, with milling times adjusted accordingly.

When comparing the results obtained for the LAG Wittig olefination to the halogen exchange reaction from **Section 2.4.2**, a significant difference becomes apparent: in the halogen exchange reaction, attempts to level the yields at different frequencies by keeping E_{total} constant failed, with yields "overshooting" at longer reaction times. In contrast, for the Wittig olefination, equalization of the reaction yield by maintaining a constant E_{total} was relatively accurate, with average yield differences of at most 20% (compared to >60% for the all-solid halogen exchange reaction). We hypothesize that these differences are due to the fundamentally distinct reaction regimes, particularly in terms of aging behaviour. The halogen-exchange reaction showed slow aging, which could explain the "overshooting" in yields at extended milling times. In contrast, the Wittig olefination, which shows no macroscopically observable aging, appears to reflect predominantly the conversion occurring at the moment of mechanical impact. We further assume that, when the minimum threshold energy required for conversion is reached for each single impact, the total energy may serve as a useful parameter to level reaction yields in a ball milling system using a single milling ball (with a ball diameter close to the inner diameter of the milling vessel), at least for reactions that do not exhibit aging or for which any aging process is so rapid and complete that its macroscopic effects are negligible.

4.4.3 Yields at constant milling time and frequency using 15 mm milling balls of different materials and masses under all-solid conditions with variable amounts of LAG additives

In the following experiments, we aimed to investigate whether the influence of E_{impact} on reaction kinetics observed in the all-solid halogen exchange reaction (see **Section 2.4.3**) also applies to the Wittig olefination. Of particular interest was whether the impact energy continues to drive the reaction when increasing amounts of LAG agent are added, or the number of net impacts becomes a more significant factor under LAG-supported conditions. As discussed previously, if the number of net impacts were the sole driving force, the reaction yield would be expected to be largely independent of milling ball weight when frequency, time, and ball size are held constant.

To test this, a series of reactions were performed following General Procedure C at fixed milling time and frequency. Each reaction employed a single 15 mm milling ball, with the material—and thus the weight—varied across sets. The balls were made of tungsten carbide (WC), chrome steel (Fe-Cr), zirconia (ZrO_2), or silicon nitride (Si_3N_4). ^{t}BuOH was used as the LAG agent in different volumetric amounts to achieve η-values of 0.1, 0.5, and 1.0, where the η-value describes the volume of solvent per mass of reagents. According to literature, mechanochemical reactions with η-values between 0 and 1 are classified as typical LAG reactions, whereas reactions with η > 1 fall into the slurry regime.[5]

The milling ball specifications and the resulting yields of product **4** are summarized in the following tables, with the results visualized in the corresponding charts.

No LAG

Table S-26. Reaction parameters and yields of **4** for reactions ball milled for a fixed time of 10 min at a constant frequency of 25 Hz using 15 mm milling balls of different materials under solvent-free conditions (no LAG added).

entry	ball size [mm]	ball material	material density [g/cm³]	ball mass [g]	milling time [min]	frequency [Hz]	calc. E_{impact} [J]	calc. E_{total} [J]	temperature [°C]	yield (4)	average yield (4) ± std. dev.
1	15	WC	14.90	26.226	10.00	25	0.291	42618		99%	
2	15	WC	14.90	26.234	10.00	25	0.291	42631	31	97%	98 ± 1%
3	15	WC	14.90	26.257	10.00	25	0.292	42668		98%	
4	15	Fe-Cr	7.70	13.553	10.00	25	0.150	22024		70%	
5	15	Fe-Cr	7.70	13.586	10.00	25	0.151	22077	48	79%	73 ± 6%
6	15	Fe-Cr	7.70	13.590	10.00	25	0.151	22084		68%	
7	15	ZrO_2	6.10	11.333	10.00	25	0.126	18416		58%	
8	15	ZrO_2	6.10	11.337	10.00	25	0.126	18423	40	55%	55 ± 2%
9	15	ZrO_2	6.10	11.348	10.00	25	0.126	18428		54%	
10	15	Si_3N_4	3.20	5.723	10.00	25	0.064	9300		22%	
11	15	Si_3N_4	3.20	5.741	10.00	25	0.064	9329	38	21%	23 ± 3%
12	15	Si_3N_4	3.20	5.762	10.00	25	0.064	9363		27%	

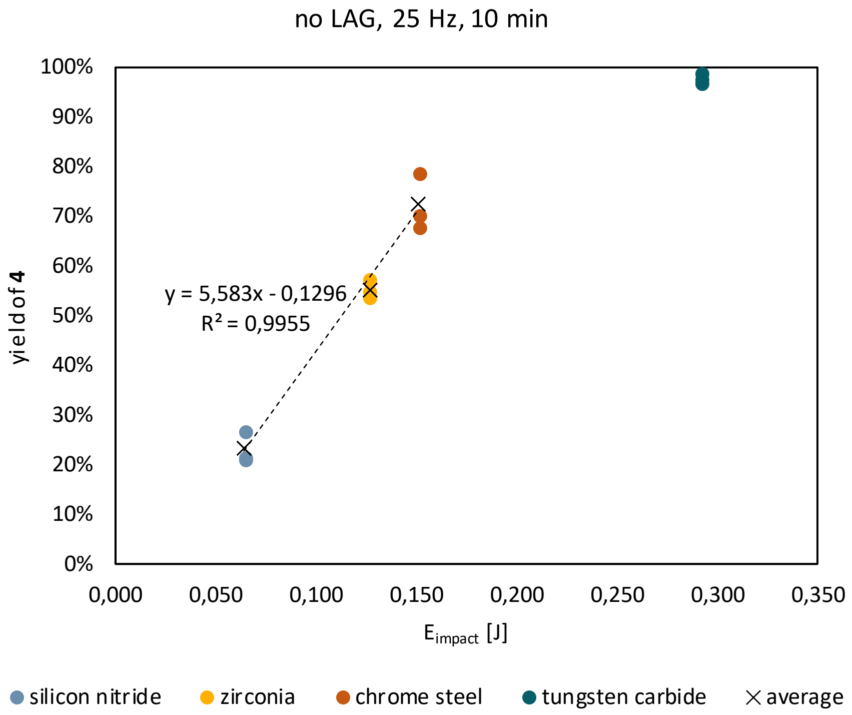

Chart S-23. Yields of **4** as a function of E_{impact} [J] performed by ball milling for a fixed time of 10 min at 25 Hz using 15 mm milling balls made from different materials, without the addition of a LAG agent. Black crosses indicate the average yield for each set of reactions conducted with identical E_{impact}, and the trendline represents the linear correlation of these averages.

21 µL ^{t}BuOH as LAG (η = 0.1)

Table S-27. Reaction parameters and yields of **4** for reactions ball milled for a fixed time of 10 min at a constant frequency of 25 Hz using 15 mm milling balls of different materials with 21 µL ^{t}BuOH (η = 0.1) as LAG additive.

**entry**	**ball size** [mm]	**ball material**	**material density** [g/cm³]	**ball mass** [g]	**milling time** [min]	**frequency** [Hz]	**calc.** E_{impact} [J]	**calc.** E_{total} [J]	**temperature** [°C]	**yield (4)**	**average yield (4)** ± std. dev.
1	15	WC	14.90	26.226	10.00	25	0.291	42618		67%	
2	15	WC	14.90	26.234	10.00	25	0.291	42631	31	58%	62 ± 4%
3	15	WC	14.90	26.257	10.00	25	0.292	42668		62%	
4	15	Fe-Cr	7.70	13.553	10.00	25	0.150	22024		35%	
5	15	Fe-Cr	7.70	13.586	10.00	25	0.151	22077	39	37%	38 ± 4%
6	15	Fe-Cr	7.70	13.590	10.00	25	0.151	22084		43%	
7	15	ZrO_2	6.10	11.333	10.00	25	0.126	18416		31%	
8	15	ZrO_2	6.10	11.337	10.00	25	0.126	18423	37	31%	33 ± 3%
9	15	ZrO_2	6.10	11.348	10.00	25	0.126	18428		35%	
10	15	Si_3N_4	3.20	5.723	10.00	25	0.064	9300		26%	
11	15	Si_3N_4	3.20	5.741	10.00	25	0.064	9329	38	27%	26 ± 1%
12	15	Si_3N_4	3.20	5.762	10.00	25	0.064	9363		25%	

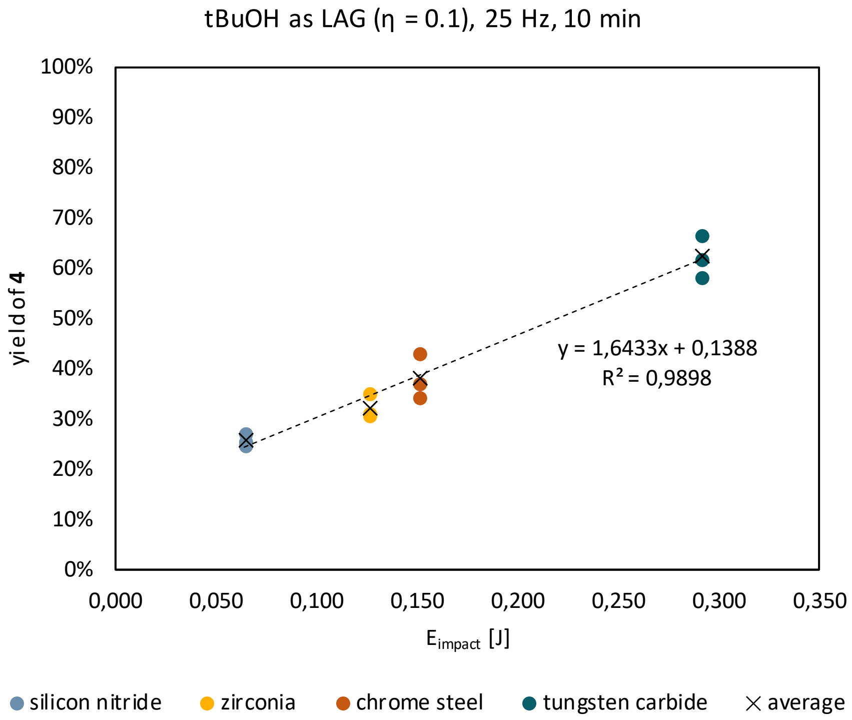

Chart S-24. Yields of **4** as a function of E_{impact} [J] performed by ball milling for a fixed time of 10 min at 25 Hz using 15 mm milling balls made from different materials, with 21 µL ^{t}BuOH (η = 0.1) as LAG additive. Black crosses indicate the average yield for each set of reactions conducted with identical E_{impact}, and the trendline represents the linear correlation of these averages.

Table S-28. Reaction parameters and yields of **4** for reactions ball milled for a fixed time of 10 min at a constant frequency of 30 Hz using 15 mm milling balls of different materials with 21 µL ^{t}BuOH (η = 0.1) as LAG additive.

entry	ball size [mm]	ball material	material density [g/cm³]	ball mass [g]	milling time [min]	frequency [Hz]	calc. E_{impact} [J]	calc. E_{total} [J]	temperature [°C]	yield (4)	average yield (4) ± std. dev.
1	15	WC	14.90	26.226	10.00	30	0.419	73643		99%	
2	15	WC	14.90	26.234	10.00	30	0.419	73666	50	98%	99 ± 1%
3	15	WC	14.90	26.257	10.00	30	0.420	73730		99%	
4	15	Fe-Cr	7.70	13.553	10.00	30	0.217	38057		60%	
5	15	Fe-Cr	7.70	13.586	10.00	30	0.217	38150	45	58%	61 ± 4%
6	15	Fe-Cr	7.70	13.590	10.00	30	0.217	38161		66%	
7	15	ZrO_2	6.10	11.333	10.00	30	0.181	31823		58%	
8	15	ZrO_2	6.10	11.337	10.00	30	0.181	31835	45	58%	56 ± 3%
9	15	ZrO_2	6.10	11.348	10.00	30	0.181	31865		52%	
10	15	Si_3N_4	3.20	5.723	10.00	30	0.092	16070		29%	
11	15	Si_3N_4	3.20	5.741	10.00	30	0.092	16121	46	32%	31 ± 2%
12	15	Si_3N_4	3.20	5.762	10.00	30	0.092	16180		32%	

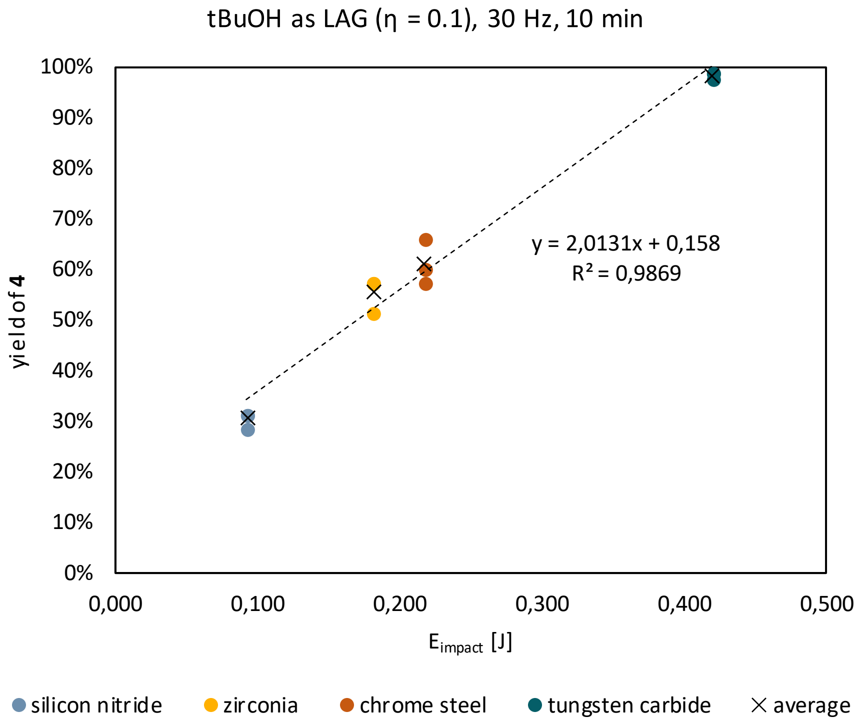

Chart S-25. Yields of **4** as a function of E_{impact} [J] performed by ball milling for a fixed time of 10 min at 30 Hz using 15 mm milling balls made from different materials, with 21 µL ^{t}BuOH (η = 0.1) as LAG additive. Black crosses indicate the average yield for each set of reactions conducted with identical E_{impact}, and the trendline represents the linear correlation of these averages.

107 µL ^{t}BuOH as LAG (η = 0.5)

Table S-29. Reaction parameters and yields of **4** for reactions ball milled for a fixed time of 10 min at a constant frequency of 25 Hz using 15 mm milling balls of different materials with 107 µL ^{t}BuOH (η = 0.5) as LAG additive.

entry	ball size [mm]	ball material	material density [g/cm³]	ball mass [g]	milling time [min]	frequency [Hz]	calc. E_{impact} [J]	calc. E_{total} [J]	temperature [°C]	yield (4)	average yield (4) ± std. dev.
1	15	WC	14.90	26.226	10.00	25	0.291	42618		86%	
2	15	WC	14.90	26.234	10.00	25	0.291	42631	42	91%	91 ± 5%
3	15	WC	14.90	26.257	10.00	25	0.292	42668		95%	
4	15	Fe-Cr	7.70	13.553	10.00	25	0.150	22024		60%	
5	15	Fe-Cr	7.70	13.586	10.00	25	0.151	22077	35	71%	67 ± 6%
6	15	Fe-Cr	7.70	13.590	10.00	25	0.151	22084		70%	
7	15	ZrO_2	6.10	11.333	10.00	25	0.126	18416		60%	
8	15	ZrO_2	6.10	11.337	10.00	25	0.126	18423	34	60%	61 ± 2%
9	15	ZrO_2	6.10	11.348	10.00	25	0.126	18428		63%	
10	15	Si_3N_4	3.20	5.723	10.00	25	0.064	9300		46%	
11	15	Si_3N_4	3.20	5.741	10.00	25	0.064	9329	30	41%	43 ± 2%
12	15	Si_3N_4	3.20	5.762	10.00	25	0.064	9363		43%	

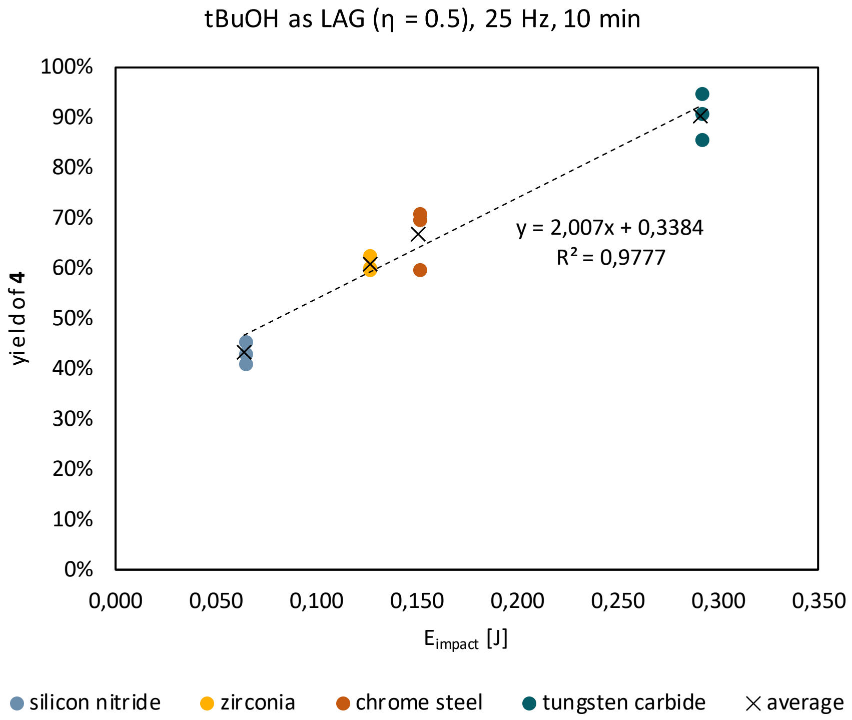

Chart S-26. Yields of **4** as a function of E_{impact} [J] for reactions performed by ball milling for a fixed time of 10 min at 25 Hz using 15 mm milling balls made from different materials, with 107 µL ^{t}BuOH (η = 0.5) as LAG additive. Black crosses indicate the average yield for each set of reactions conducted at identical E_{impact}, and the trendline represents the linear correlation of these averages.

213 µL ^{t}BuOH as LAG (η = 1.0)

Table S-30. Reaction parameters and yields of **4** for reactions ball milled for a fixed time of 10 min at a constant frequency of 25 Hz using 15 mm milling balls of different materials with 213 µL ^{t}BuOH (η = 1.0) as LAG additive.

entry	ball size [mm]	ball material	material density [g/cm³]	ball mass [g]	milling time [min]	frequency [Hz]	calc. E_{impact} [J]	calc. E_{total} [J]	temperature [°C]	yield (4)	average yield (4) ± std. dev.
1	15	WC	14.90	26.226	10.00	25	0.291	42618		91%	
2	15	WC	14.90	26.234	10.00	25	0.291	42631	47	85%	89 ± 3%
3	15	WC	14.90	26.257	10.00	25	0.292	42668		90%	
4	15	Fe-Cr	7.70	13.553	10.00	25	0.150	22024		62%	
5	15	Fe-Cr	7.70	13.586	10.00	25	0.151	22077	32	64%	62 ± 2%
6	15	Fe-Cr	7.70	13.590	10.00	25	0.151	22084		61%	
7	15	ZrO_2	6.10	11.333	10.00	25	0.126	18416		50%	
8	15	ZrO_2	6.10	11.337	10.00	25	0.126	18423	31	55%	54 ± 3%
9	15	ZrO_2	6.10	11.348	10.00	25	0.126	18428		56%	
10	15	Si_3N_4	3.20	5.723	10.00	25	0.064	9300		42%	
11	15	Si_3N_4	3.20	5.741	10.00	25	0.064	9329	34	38%	42 ± 4%
12	15	Si_3N_4	3.20	5.762	10.00	25	0.064	9363		47%	

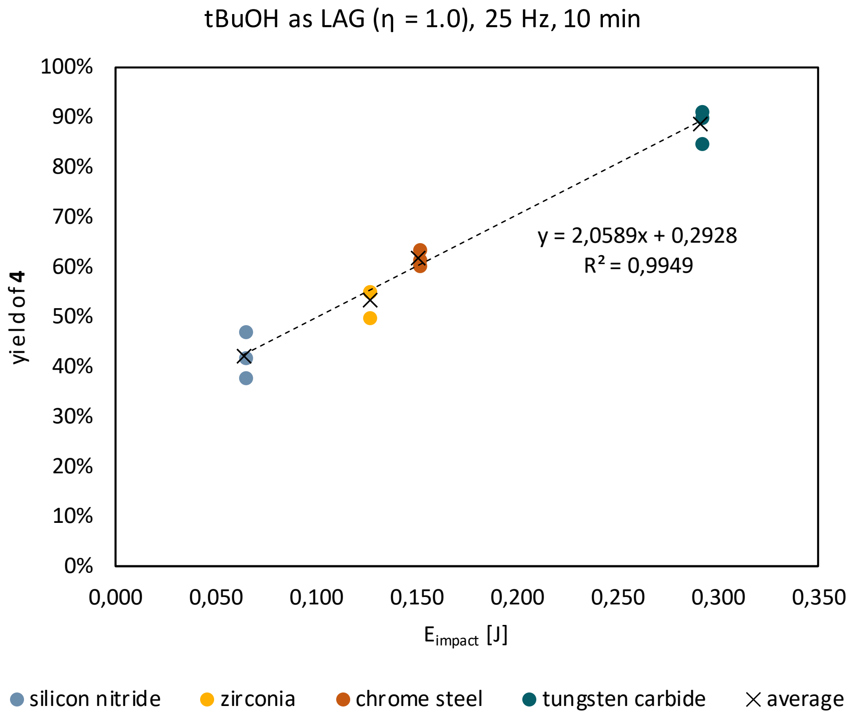

Chart S-27. Yields of **4** as a function of E_{impact} [J] for reactions performed by ball milling for a fixed time of 10 min at 25 Hz using 15 mm milling balls made from different materials, with 213 µL ^{t}BuOH (η = 1.0) as LAG additive. Black crosses indicate the average yield for each set of reactions conducted at identical E_{impact}, and the trendline represents the linear correlation of these averages.

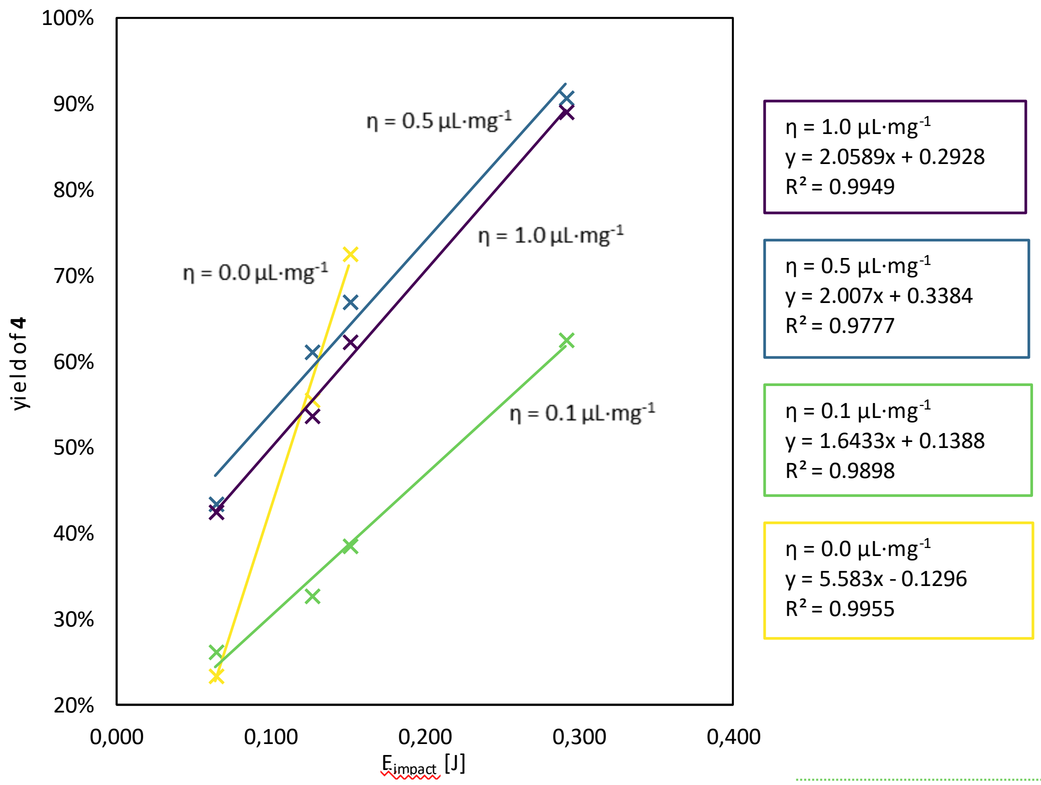

Chart S-28. Combined results of LAG studies of the Wittig olefination using different amounts of ^{t}BuOH as LAG agent. The datapoints (crosses) correspond to the average yield of triplicate reactions. Yield of **4** is shown as a function of E_{impact} [J].

Interestingly, these results indicate that even under LAG conditions - using a solvent not typically applied in Wittig olefinations and thus assumed not to accelerate the reaction, but still capable of partially solubilizing the reaction components - the energy of the single impact (E_{impact}), rather than the number of net impacts, appears to be the primary factor driving the olefination reaction.

4.4.4 Yields at constant impact energy (E_{impact}) via ball mass and frequency variation at constant milling times

Next, we aimed to investigate the effect of the single impact energy on the olefination reaction, in analogy to previous studies (see **Sections 2.4.4** and **3.4.2**). A set of experiments was performed with a constant milling time of 10 min, while the milling frequency was adjusted according to the milling ball weight to achieve a comparable theoretical impact energy (E_{impact}, calculated according to Lungerich).[1] Each experiment followed the general procedure C, with ball milling parameters and material specifications provided in the respective tables. The frequencies were selected to maintain E_{impact} within a narrow range of 0.116–0.119 J.

Table S-31. Reaction parameters and yields of **4** for reactions ball milled for a fixed reaction time of 10 min, with milling frequencies adjusted according to the milling ball mass to maintain the single impact energy (E_{impact}) within a narrow range of 0.116–0.119 J.

entry	ball size [mm]	ball material	material density [g/cm³]	ball mass [g]	milling time [min]	frequency [Hz]	calc. E_{impact} [J]	calc. E_{total} [J]	temperature [°C]	yield (4)	average yield (4) ± std. dev.
1	15	WC	14.90	26.226	10.00	16	**0.119**	11172		26%	
2	15	WC	14.90	26.234	10.00	16	**0.119**	11175	31	22%	24 ± 2%
3	15	WC	14.90	26.257	10.00	16	**0.119**	11185		25%	
4	15	Fe-Cr	7.70	13.553	10.00	22	**0.117**	15009		34%	
5	15	Fe-Cr	7.70	13.586	10.00	22	**0.117**	15045	39	38%	35 ± 2%
6	15	Fe-Cr	7.70	13.590	10.00	22	**0.117**	15050		34%	
7	15	ZrO_2	6.10	11.333	10.00	24	**0.116**	16294		43%	
8	15	ZrO_2	6.10	11.337	10.00	24	**0.116**	16299	34	44%	43 ± 1%
9	15	ZrO_2	6.10	11.348	10.00	24	**0.116**	16315		41%	
10	15	Si_3N_4	3.20	5.723	10.00	34	**0.118**	23394		73%	
11	15	Si_3N_4	3.20	5.741	10.00	34	**0.118**	23467	41	74%	69 ± 8%
12	15	Si_3N_4	3.20	5.762	10.00	34	**0.118**	23553		59%	

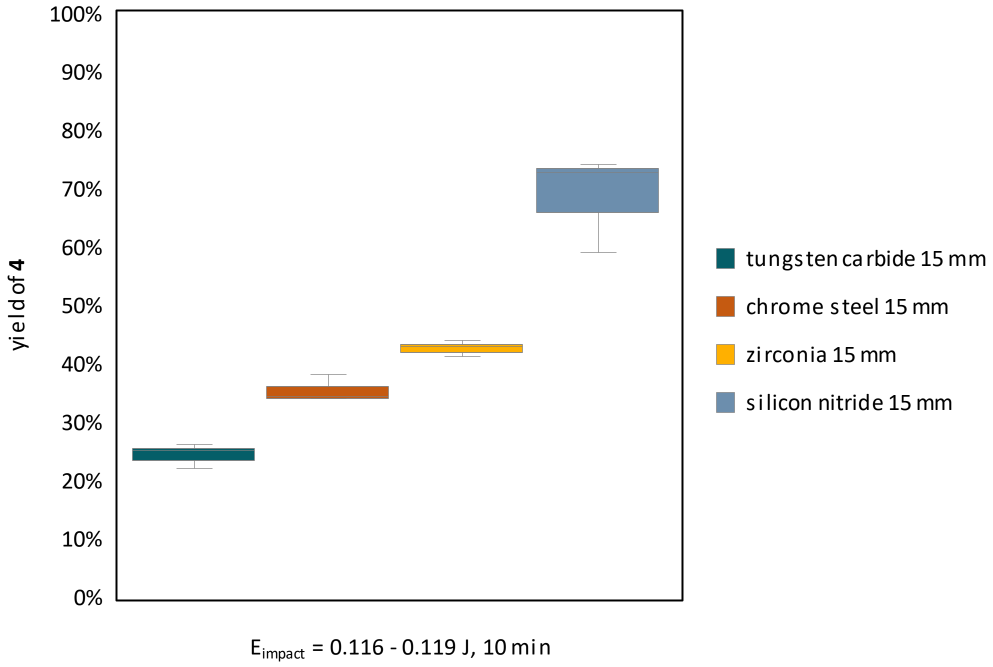

Chart S-29. Yields of **4** for reactions ball milled for a fixed reaction time of 10 min, with milling frequencies adjusted according to the milling ball mass to maintain the single impact energy (E_{impact}) within a narrow range of 0.116–0.119 J.

4.4.5 Yields at Comparable E_{impact} via Frequency Adjustment for Ball Milling Masses with Total Energy (E_{total}) Equalized by Adjusting Milling Times to Standardize the Number of Theoretical Impacts

Analogous to the procedures described in **Sections 2.4.5** and **3.4.3**, the reaction times of the target experiments were adjusted ($t_{adjusted}$) relative to a reference experiment conducted at a reference frequency ($f_{reference}$) for a reference milling time ($t_{reference}$) using a frequency ratio (FR), in order to equalize the number of impacts between the target and reference experiments according to Eq. 2 and 3.

In the tables and corresponding charts below, the reference experiment is color-coded in green. The times reported correspond to the adjusted times ($t_{adjusted}$) calculated using the equations provided in **Section 2.4.5**. All reactions were conducted following General Procedure C. Milling parameters and other experimental details are provided in the respective tables.

It should be noted that at 16 Hz, using a relatively heavy tungsten carbide milling ball, the ball movement is somewhat reluctant and inert at this low frequency, likely resulting in a significantly reduced number of effective impact events.

Table S-32. Reaction parameters and yields of **4** for reactions with comparable E_{impact} and E_{total}. Reference experiments (entries 10–12, green); for all other entries, milling times were adjusted to match the number of impacts to the reference experiment.

entry	ball size [mm]	ball material	material density [g/cm³]	ball mass [g]	milling time [min]	frequency [Hz]	calc. E_{impact} [J]	calc. E_{total} [J]	temperature [°C]	yield (4)	average yield (4) ± std. dev.
1	15	WC	14.90	26.226	**21.25**	16	**0.119**	23740		53%	
2	15	WC	14.90	26.234	**21.25**	16	**0.119**	23748	40	62%	56 ± 5%
3	15	WC	14.90	26.257	**21.25**	16	**0.119**	23768		54%	
4	15	Fe-Cr	7.70	13.553	**15.45**	22	**0.117**	23188		63%	
5	15	Fe-Cr	7.70	13.586	**15.45**	22	**0.117**	23245	40	64%	65 ± 2%
6	15	Fe-Cr	7.70	13.590	**15.45**	22	**0.117**	23252		67%	
7	15	ZrO_2	6.10	11.333	**14.17**	24	**0.116**	23088		67%	
8	15	ZrO_2	6.10	11.337	**14.17**	24	**0.116**	23096	45	67%	68 ± 3%
9	15	ZrO_2	6.10	11.348	**14.17**	24	**0.116**	23119		72%	
10	15	Si_3N_4	3.20	5.723	10.00	34	**0.118**	23394		73%	
11	15	Si_3N_4	3.20	5.741	10.00	34	**0.118**	23467	41	74%	69 ± 8%
12	15	Si_3N_4	3.20	5.762	10.00	34	**0.118**	23553		59%	

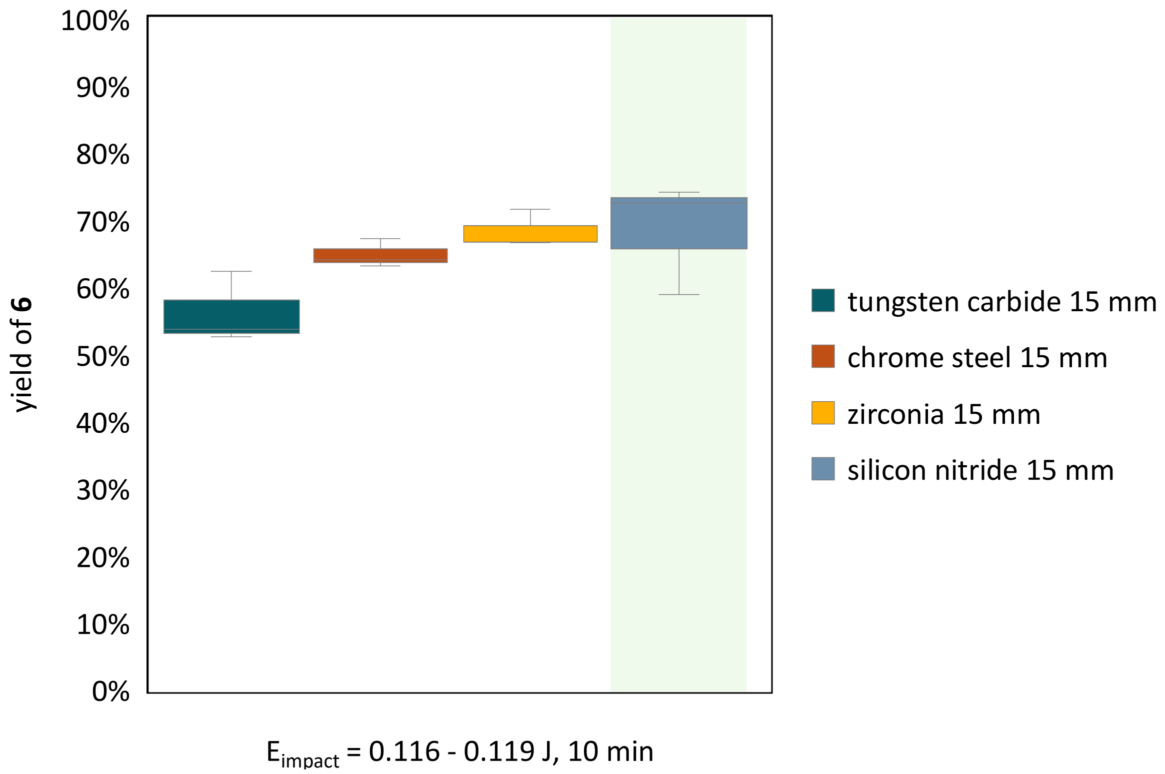

Chart S-30. Yields of **4** for reactions with comparable E_{impact} and E_{total}. Reference experiments (silicon nitride, 15 mm) are displayed in the rightmost column (4th box); for all other entries, milling times were adjusted to match the number of impacts to the reference experiment.

5. References

[1] O. F. Jafter, S. Lee, J. Park, C. Cabanetos, D. Lungerich, "Navigating Ball Mill Specifications for Theory-to-Practice Reproducibility in Mechanochemistry" *Angewandte Chemie International Edition* **2024**, *63*, e202409731.

[2] A. Shaabani, P. Mirzaei, S. Naderi, D. G. Lee, "Green oxidations. The use of potassium permanganate supported on manganese dioxide" *Tetrahedron* **2004**, *60*, 11415-11420.

[3] F. Mele, N. Biedermann, C. Suster, J. Templ, C. Stanetty, M. Schnürch, "Mechanochemistry Enables Rapid and Solvent-Free Wittig Reactions on Sugars" *ChemRxiv* **2025**, DOI: 10.26434/chemrxiv-22025-b26437bdr-v26432.

[4] J. Templ, M. Schnürch, "High-Energy Ball Milling Enables an Ultra-Fast Wittig Olefination Under Ambient and Solvent-free Conditions" *Angewandte Chemie International Edition* **2024**, *63*, e202411536.

[5] D. Tan, T. Friščić, "Mechanochemistry for Organic Chemists: An Update" *European Journal of Organic Chemistry* **2018**, *2018*, 18-33.
